# Dynamic interplay between the co-opted Fis1 mitochondrial fission protein and membrane contact site proteins in supporting tombusvirus replication

**DOI:** 10.1371/journal.ppat.1009423

**Published:** 2021-03-16

**Authors:** Wenwu Lin, Zhike Feng, K. Reddisiva Prasanth, Yuyan Liu, Peter D. Nagy

**Affiliations:** 1 Department of Plant Pathology, University of Kentucky, Lexington, United States of America; 2 State Key Laboratory of Ecological Pest Control for Fujian and Taiwan Crops, Fujian Agriculture and Forestry University, Fuzhou, China; Institute of Microbiology, CHINA

## Abstract

Plus-stranded RNA viruses have limited coding capacity and have to co-opt numerous pro-viral host factors to support their replication. Many of the co-opted host factors support the biogenesis of the viral replication compartments and the formation of viral replicase complexes on subverted subcellular membrane surfaces. Tomato bushy stunt virus (TBSV) exploits peroxisomal membranes, whereas the closely-related carnation Italian ringspot virus (CIRV) hijacks the outer membranes of mitochondria. How these organellar membranes can be recruited into pro-viral roles is not completely understood. Here, we show that the highly conserved Fis1 mitochondrial fission protein is co-opted by both TBSV and CIRV via direct interactions with the p33/p36 replication proteins. Deletion of *FIS1* in yeast or knockdown of the homologous Fis1 in plants inhibits tombusvirus replication. Instead of the canonical function in mitochondrial fission and peroxisome division, the tethering function of Fis1 is exploited by tombusviruses to facilitate the subversion of membrane contact site (MCS) proteins and peroxisomal/mitochondrial membranes for the biogenesis of the replication compartment. We propose that the dynamic interactions of Fis1 with MCS proteins, such as the ER resident VAP tethering proteins, Sac1 PI4P phosphatase and the cytosolic OSBP-like oxysterol-binding proteins, promote the formation and facilitate the stabilization of virus-induced vMCSs, which enrich sterols within the replication compartment. We show that this novel function of Fis1 is exploited by tombusviruses to build nuclease-insensitive viral replication compartment.

## Author summary

Positive-strand RNA viruses, which include many dangerous pathogens of animals and plants, require the biogenesis of viral replication compartments. Utilizing TBSV and the model host yeast, we found a critical and novel role for the Fis1 mitochondrial fission protein. The pro-viral function of Fis1 is manifested through binding to the TBSV p33 replication protein that results in recruitment into the replication compartment formed from aggregated peroxisomes. Fis1 also binds to the related carnation Italian ringspot virus (CIRV) p36 replication protein in the replication compartment formed from aggregated mitochondria. Instead of the canonical function in mitochondria fission or peroxisome division, the tethering function of Fis1 is exploited by TBSV in facilitating the recruitment of cellular membrane contact site proteins into the viral replication compartment. Altogether, co-opting Fis1 is critical for tombusviruses to build their replication compartments in infected cells. Targeting these organelle-tethering proteins by inhibitors or other ways could open up new antiviral strategies.

## Introduction

Due to the rather limited coding capacity of their small RNA genomes, positive-strand RNA viruses co-opt many pro-viral host factors to support their replication. (+)RNA virus replication requires the biogenesis of viral replication compartments, also called viral replication organelles (VROs). The VROs harbor numerous viral replicase complexes (VRCs) on hijacked subcellular membrane surfaces, which replicate the viral RNAs [1–7]. In addition to co-opting cellular proteins, viruses also bind to phospholipids and sterols, deform membranes, and modulate lipid biosynthesis and lipid transfer and vesicular trafficking [8–12]. All these cellular changes, including the hijacked cellular membranes, serve the purpose to protect the viral RNA, including the dsRNA replication intermediate, from recognition and elimination by the cellular innate immune system [13,14]. The membranous VROs also help concentrate viral and co-opted host proteins together with the viral (+)RNA. All these processes are needed for optimal VRCs formation [9,11,15–21]. This is a rapidly expanding research field with possible applications and new therapies to control RNA virus replication.

Tombusviruses, such as tomato bushy stunt virus (TBSV), are small (+)RNA viruses of plants, which can replicate in the surrogate host yeast (*Saccharomyces cerevisiae*) [22–24]. Intensive research with TBSV–yeast system identified numerous host factors co-opted for viral RNA replication [4,24,25]. In addition, a major role for global phospholipid and sterol biosynthesis has been revealed [26–28]. Also critical roles of phospatidylethanolamine (PE), phospatidylserine, phosphoinositides and sterols in formation of VRCs and activation of the viral-coded p92 RdRp has been shown [29–38]. Sterols and PE bind directly to the p33 replication protein, which is the master regulator of VRC assembly and viral (+)RNA recruitment into VRCs [29,39].

Among the most characteristic alterations caused by TBSV in the host cells include the induction of subcellular membrane proliferation, peroxisome aggregation and the formation of membrane contact sites (MCSs) to support efficient virus replication [24,40–42]. In spite of these major changes, previous genetic and proteomic screens with TBSV have not identified many peroxisome biogenesis proteins that might be involved in TBSV replication in yeast [43–49]. The notable exception is the critical pro-viral role of Rpn11 proteasomal deubiqutinase in TBSV replication [50], which is also involved in peroxisomal division [51]. Pex19 is a peroxisomal transporter involved in the localization of p33 replication protein to the host peroxisomal membranes [52]. However, *PEX19* is not essential for TBSV replication, and TBSV replication takes place in the ER membrane in the absence of *PEX19* [53]. Therefore, to test the putative role of peroxisome division on TBSV replication, here we extended our studies on the possible involvement of the major peroxisomal division proteins in TBSV replication in yeast. To this end, we have found a critical role only for the Fis1p mitochondrial fission protein, but not for the other fission complex proteins. Fis1p is a tail-anchored membrane protein with a cytosolic domain, which is targeted to both the mitochondrial and peroxisomal membranes by Pex19 [54,55]. Fis1p is known to recruit the other fission proteins to the site of the fission in peroxisomal or mitochondrial membranes [56,57]. In addition, Fis1p initiates interaction between mitochondria and ER through binding to the ER resident Bap31 protein, which then serves as a platform to assemble the apoptotic complex (called ARCosome, a caspase-activation complex) during apoptosis in mammalian cells [58–60]. Fis1p is involved in tethering protein aggregates to mitochondria for degradation and prevention of damage caused by the aggregates [61]. Fis1p can capture and cluster lipid vesicles in vitro [62]. Fis1 is conserved in plants and animals and it might play similar roles although Fis1 function is less characterized than in yeast [63,64]. *Arabidopsis thaliana* contains two homologs, Fis1A and Fis1B, which show 58% amino acid identity. Both AtFis1A and AtFis1B have similar protein structures to the yeast Fis1p and they might participate in mithochondrial fission and peroxisomal division [64,65].

Therefore, we decided to characterize the molecular function of Fis1p during TBSV replication. We find that Fis1p binds to the TBSV p33 replication protein that results in recruitment into the replication compartment formed from aggregated peroxisomes. Fis1p also binds to the closely-related carnation Italian ringspot virus (CIRV) p36 replication protein in the replication compartment formed from aggregated mitochondria. Instead of the canonical function in mitochondria fission or peroxisome division, Fis1p is found to play a pro-viral role in facilitating the recruitment of cellular MCS proteins, such as the ER-resident Scs2p/VAP27 VAP tethering proteins, Sac1p PI4P phosphatase and the oxysterol-binding proteins, into the viral replication compartment. We propose that Fis1p serves as a co-opted tethering protein in the peroxisomes/mitochondria to facilitate the stable formation of vMCSs involving peroxisomes and the ER membrane for TBSV and mitochondria and the ER membrane in case of CIRV. Altogether, co-opting Fis1p is critical for tombusviruses to build their replication compartments in infected cells.

## Results

### Fis1 mitochondrial fission protein is required for tombusvirus replication

**Fig 1:**
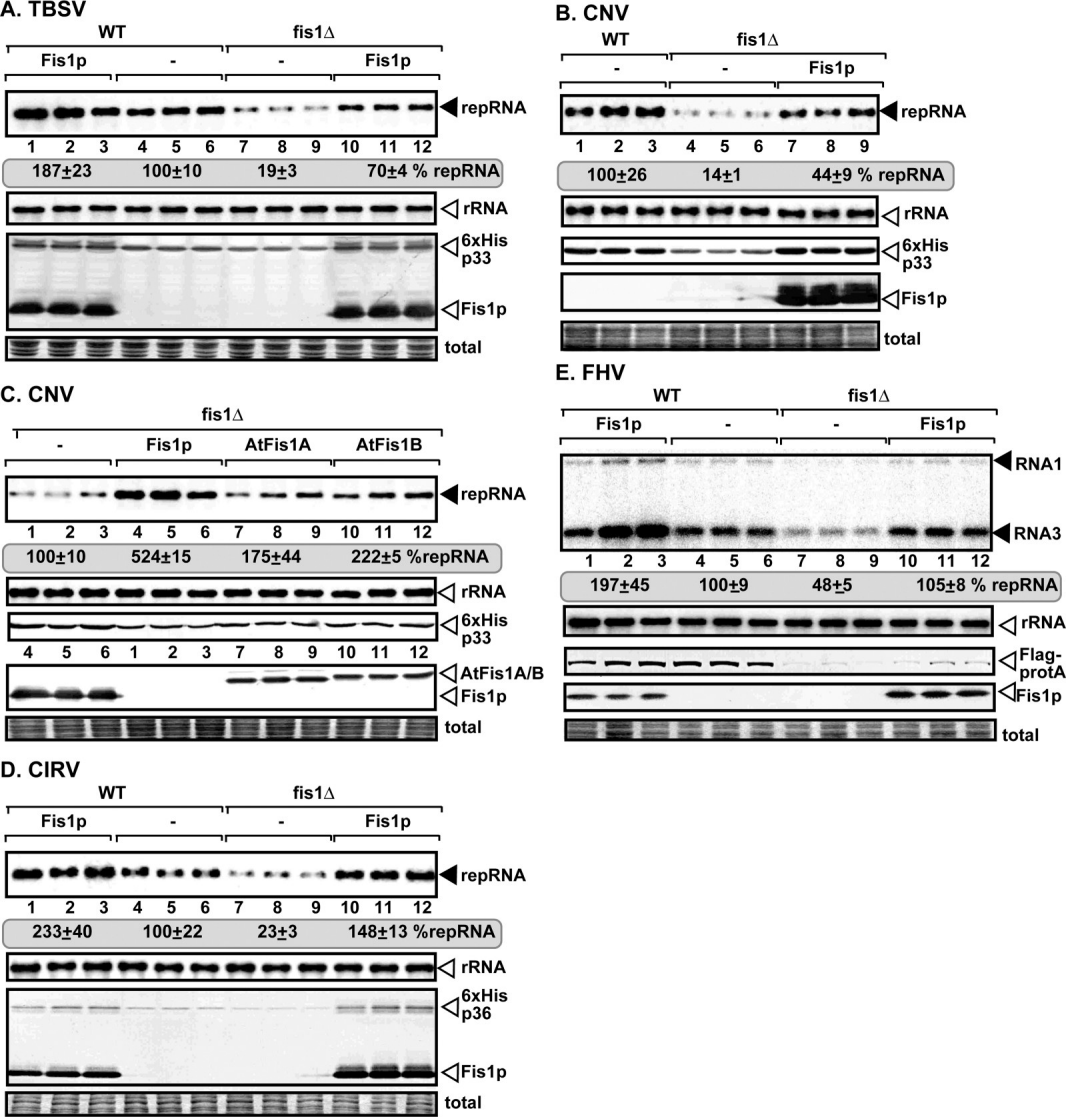
Fis1p mitochondrial fission 1 protein is an essential host factor for tombusvirus replication in yeast. (A-D) Deletion of *FIS1* inhibits TBSV, CNV, and CIRV replication in yeast. Top panels: northern blot analyses of repRNA using a 3’ end specific probe demonstrate reduced accumulation of repRNA in fis1Δ yeast strain in comparison with the WT yeast strain. Viral proteins His_6_-p33 and His_6_-p92 of TBSV were expressed from plasmids from the galactose-inducible *GAL1* promoter, whereas the Flag-p33 and Flag-p92 of CNV and the CIRV His_6_-p36 and His_6_-p95 were expressed from plasmids from the copper-inducible *CUP1* promoter. The DI-72(+) replicon (rep)RNA was expressed from the *GAL10* promoter. His_6_-Fis1 was expressed from the *GAL1* promoter from a plasmid. Second panel: northern blot with 18S ribosomal RNA specific probe was used as a loading control. Bottom images: western blot analysis of the level of His_6_-tagged proteins and Flag-tagged proteins with anti-His or anti-Flag-antibodies, respectively. Coomassie blue-stained SDS-PAGE was used as protein loading control. (E) Deletion of *FIS1* inhibits the unrelated FHV replication in yeast. FHV RNA1 and Flag-tagged protein A replication protein were expressed from plasmids. See further details in panel A. Each experiment was repeated three times.

To identify which major peroxisomal division proteins are involved in TBSV replication, we expressed p33 and p92^pol^ replication proteins and the replicon repRNA in yeast with a single gene deletion of one of the five genes coding for major peroxisomal division proteins. Our screen was based on northern blot analysis, which revealed that the absence of only Fis1p mitochondrial fission protein reduced TBSV repRNA accumulation significantly (by ~4-to-5-fold, S1A Fig and Fig 1A, lanes 7–9 versus 4–6), whereas the absence of dynamin-like GTPase Dnm1p and Caf4p and Mdv1p WD40 repeat proteins [63,66] did not inhibit TBSV repRNA replication (S1A Fig). Moreover, the absence of the dynamin-like Vps1 protein, involved in peroxisome biogenesis, had no inhibitory effect on TBSV replication in yeast (S1A Fig). These data suggest that Fis1p is critical for TBSV replication, but it is unlikely that the pro-viral role of Fis1p is in connection with its role in the canonical peroxisomal division, which depends on the above five host proteins. Because of major effect of Fis1p on the replication of TBSV repRNA in yeast, we decided to conduct more detailed experiments with Fis1p to identify its pro-viral functions.

To test if Fis1p is required for replication of other viruses targeting peroxisomal or mitochondrial membranes to build replication compartments, we used the closely-related cucumber necrosis virus (CNV, peroxisomal), which showed a ~5-fold decrease in replication in fis1Δ yeast (Fig 1B, lanes 4–6 versus 1–3). Comparable experiments with the closely-related carnation Italian ringspot virus (CIRV), which replicates on the cytosolic side of the outer mitochondrial membrane, revealed a ~4-fold decrease in replication in fis1Δ yeast (Fig 1D, lanes 7–9 versus 4–6). Interestingly, we also found that the unrelated Flock House virus (FHV) and Nodamura virus (NoV), two insect viruses replicating on the outer mitochondrial membranes [67], were also dependent on Fis1p (Fig 1E, lanes 7–9 versus 4–6 and S1C Fig), and to a lesser extent on Dnm1 yeast proteins (S1B and S1C Fig). Fis1 deletion apparently affected the stability of the viral replication proteins, which likely contributes to reduced replication of the above viruses in yeast.

Over-expression of the yeast Fis1p enhanced TBSV and CIRV replication by ~2-fold in WT yeast (Fig 1A–1D). Similarly, over-expression of the yeast Fis1p increased FHV and NoV replication by ~2 and ~3-fold, respectively, in WT yeast (Figs 1E and S1D). Based on these observations, Fis1p mitochondrial fission protein emerges as a new pro-viral host factor.

### Fis1 has pro-viral functions in plants

**Fig 2:**
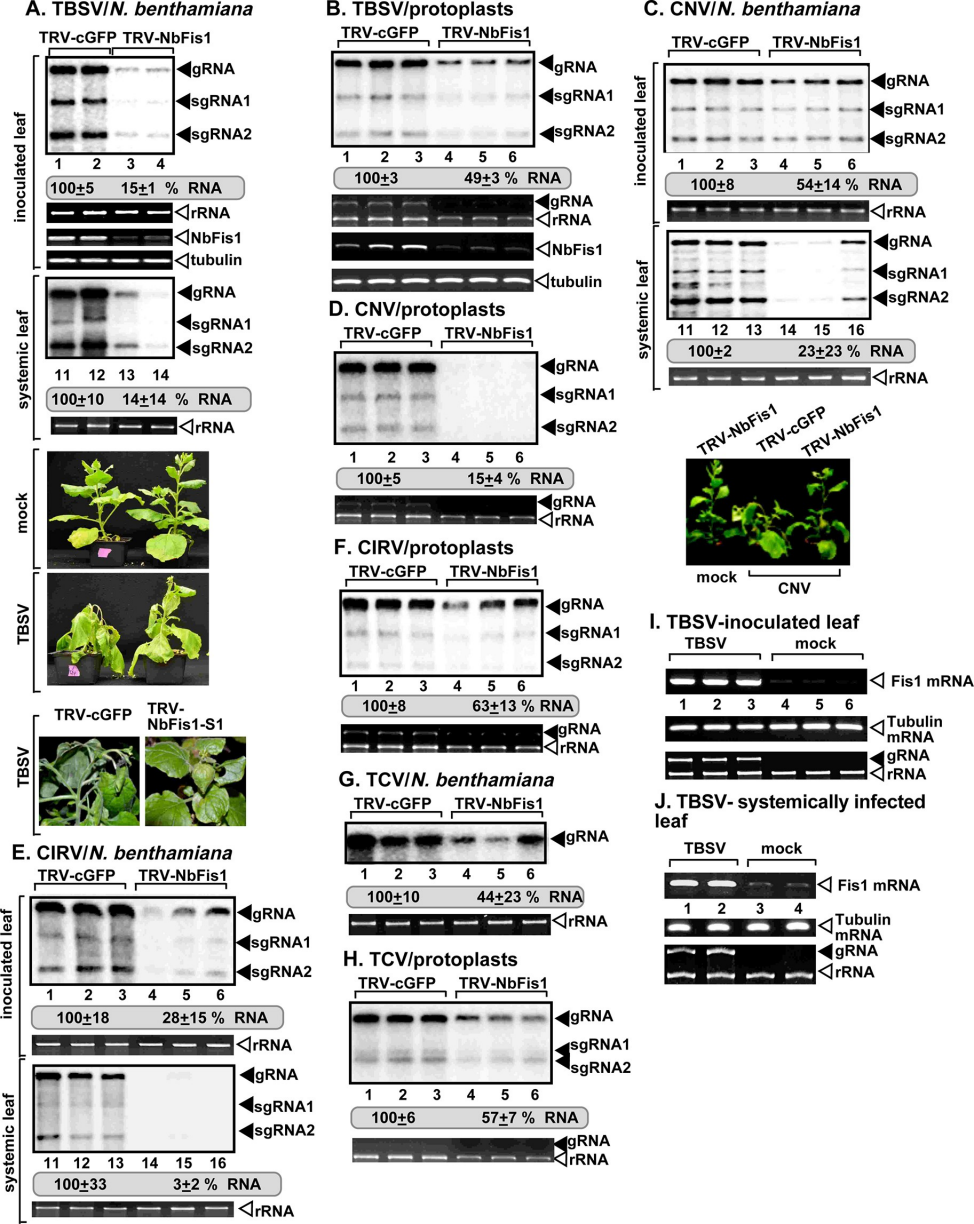
Knockdown of Fis1 mRNA level inhibits tombusvirus replication in *N*. *benthamiana* plants. (A) Top panel: The accumulation of the TBSV genomic (g)RNA in Fis1-silenced *N*. *benthamiana* plants 2 days post-inoculation (dpi) in the inoculated leaves and at 4 dpi in the systemically-infected leaves was measured by northern blot analysis. Inoculation of TBSV gRNA was done 12 days after silencing of Fis1 expression. Agroinfiltration of tobacco rattle virus (TRV) vector carrying NbFis1 or 3’-terminal GFP (as a control) sequences was used to induce VIGS. Second panel: Ribosomal RNA is shown as a loading control in an ethidium-bromide stained agarose gel. Third panel: RT-PCR analysis of NbFis1 mRNA level in the silenced and control plants. Fourth panel: RT-PCR analysis of tubulin mRNA level in the silenced and control plants. Each experiment was repeated three times. Bottom panels: Delayed development of TBSV-induced symptoms is observed in Fis1-silenced *N*. *benthamiana* plants as compared with the control plants. Note the lack of phenotype in Fis1-silenced and mock-inoculated *N*. *benthamiana* plants. Also note the wilting and beginning stage of necrosis in the control TBSV-infected plant versus the lack of those symptoms in the Fis1-silenced *N*. *benthamiana* plants. The pictures were taken at 9 dpi. (B) Top panel: The accumulation of the TBSV gRNA in protoplasts isolated from Fis1-silenced *N*. *benthamiana* was measured by northern blot analysis. Protoplasts were isolated 12 days after silencing of Fis1 expression. Agroinfiltration of TRV-NbFis1 or TRV-cGFP (as a control) was used to induce VIGS. Second panel: Ribosomal RNA is shown as a loading control in an ethidium-bromide stained agarose gel. Third panel: RT-PCR analysis of NbFis1 mRNA level in the silenced and control protoplasts. Fourth panel: RT-PCR analysis of tubulin mRNA level in the silenced and control protoplasts. Each experiment was repeated three times. (C) The accumulation of the CNV gRNA in Fis1-silenced *N*. *benthamiana* plants 3 dpi in the inoculated leaves and at 7 dpi in the systemically-infected leaves was measured by northern blot analysis. See further details in panel A. (D) Accumulation of the CNV gRNA in protoplasts isolated from Fis1-silenced *N*. *benthamiana* was measured by northern blot analysis. See further details in panel B. (E) The accumulation of the CIRV gRNA in Fis1-silenced *N*. *benthamiana* plants 3 dpi in the inoculated leaves and at 5 dpi in the systemically-infected leaves was measured by northern blot analysis. See further details in panel A. (F) The accumulation of the CIRV gRNA in protoplasts isolated from Fis1-silenced *N*. *benthamiana* was measured by northern blot analysis. See further details in panel B. (G) The accumulation of the TCV gRNA in Fis1-silenced *N*. *benthamiana* plants 6 dpi in the inoculated leaves was measured by northern blot analysis. See further details in panel A. (H) The accumulation of the TCV gRNA in protoplasts isolated from Fis1-silenced *N*. *benthamiana* was measured by northern blot analysis. See further details in panel B. (I-J) Top panel: Induction of Fis1 mRNA expression in the inoculated leaves or systemically-infected leaves of *N*. *benthamiana* plants infected with TBSV was detected by semi-quantitative RT-PCR. Middle panel: RT-PCR of tubulin mRNA was used as a control. Bottom panel: Ribosomal RNA is shown as a loading control in an ethidium-bromide stained agarose gel.

To study if tombusviruses depend on Fis1 functions in plants, first we silenced Fis1 expression based on virus-induced gene-silencing (VIGS) in *Nicotiana benthamiana* plants. Knockdown of Fis1 in *N*. *benthamiana* led to ~6-fold reduction of TBSV RNAs in both the inoculated and the systemically-infected leaves (Fig 2A). Knockdown of Fis1 level did not cause obvious phenotype in *N*. *benthamiana* (Fig 2A). The TBSV-infected leaves of Fis1 knockdown plants showed mild symptoms on the 7^th^ day after inoculation, whereas the control VIGS plants started to wilt and showed typical TBSV-induced symptoms, such as necrosis in young leaves (Fig 2A). Knockdown of Fis1 level delayed the symptom formation and necrosis in young leaves infected with TBSV even at a latter time point (10 dpi, Fig 2A). We also tested TBSV replication in Fis1 knockdown protoplasts, which supported a ~2-fold reduced TBSV RNA level in comparison with control protoplasts (Fig 2B). Similar experiments with CNV and CIRV in Fis1 knockdown plants and protoplasts also revealed a reduced level of tombusvirus accumulation (Fig 2C–2F). These data confirmed the pro-viral role of Fis1 in supporting tombusvirus replication in plants.

To test if the pro-viral function of Fis1 is needed by a more distantly-related carmovirus, we measured the accumulation of turnip crinkle virus (TCV) in *N*. *benthamiana* with knockdown of Fis1 level. The accumulation of TCV RNAs decreased by ~2-fold in plants and protoplasts with knockdown of Fis1 (Fig 2G and 2H). Thus, in addition to tombusviruses, a carmovirus also utilizes Fis1 functions to support viral replication.

RT-PCR analysis of Fis1 mRNA levels in TBSV-infected versus mock-treated *N*. *benthamiana* leaves revealed up-regulation of Fis1 mRNA level in the TBSV inoculated leaves (Fig 2I) as well as the systemically-infected leaves (Fig 2J), suggesting that TBSV replication induces high level of Fis1 expression.

### Fis1 protein interacts with the tombusvirus replication proteins

**Fig 3:**
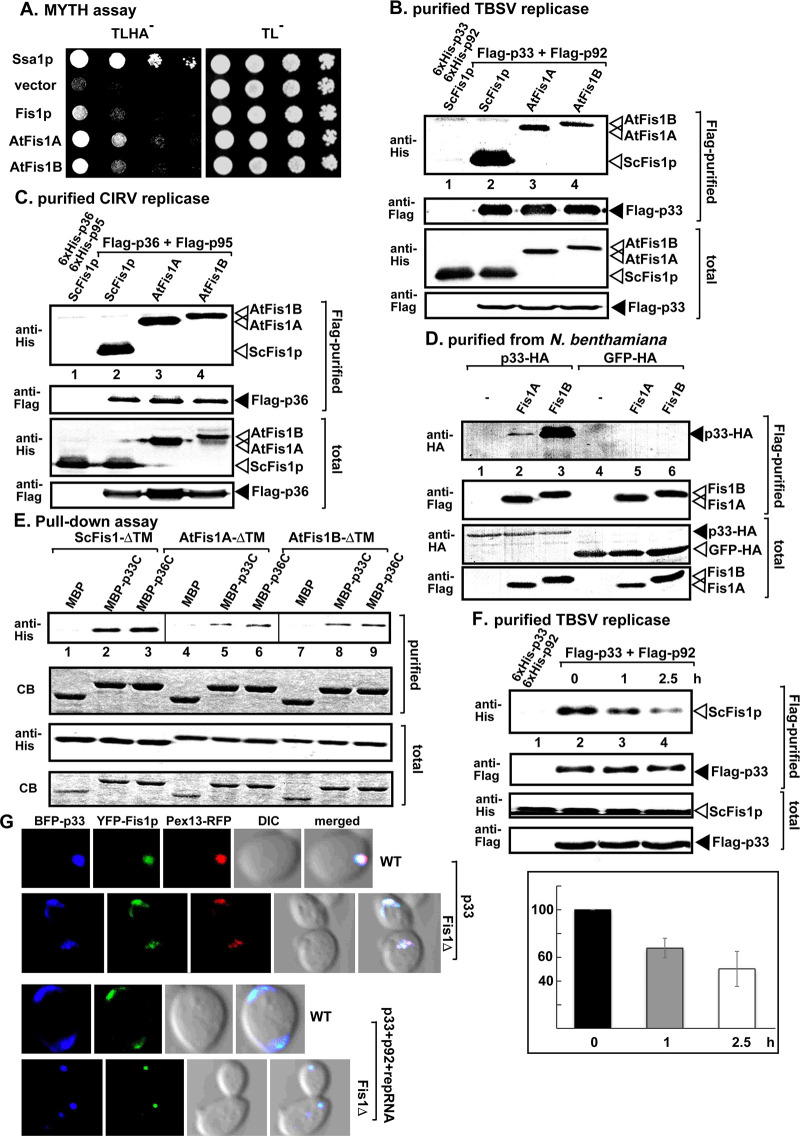
Interaction between tombusvirus replication proteins and Fis1. (A) The split ubiquitin-based MYTH assay was used to test binding between TBSV p33 and the yeast Fis1p and *Arabidopsis* Fis1 proteins in yeast. The bait p33 was co-expressed with the shown prey proteins. Ssa1p Hsp70 and the empty prey vector (NubG) were used as a positive and a negative control, respectively. The left panel shows p33: Fis1 interactions, the right panel demonstrates that comparable amounts of yeasts were used for these experiments. (B) Co-purification of the yeast His_6_-Fis1p and His_6_-AtFis1A/B with TBSV Flag-p33 and Flag-p92^pol^ replication proteins from subcellular membranes. Top two panels: western blot analysis of co-purified His_6_-Fis1p and His_6_-AtFis1A/B (lanes 2–4) with Flag-affinity purified Flag-p33 and Flag-p92^pol^. His_6_-tagged proteins were detected with anti-His antibody, while Flag-p33 was detected with anti-Flag antibody. The negative control was from yeast expressing His_6_-p33 and His_6_-p92^pol^ purified in a Flag-affinity column (lane 1). Samples were cross-linked with formaldehyde. Bottom two panels: western blot of total His_6_-Fis1p and His_6_-AtFis1A/B and Flag-p33 in the total yeast extracts. (C) Co-purification of the yeast His_6_-Fis1p and His_6_-AtFis1A/B with CIRV Flag-p36 and Flag-p95^pol^ replication proteins from subcellular membranes. See further details in panel B. (D) Co-purification of TBSV p33 replication protein with Flag-AtFis1A/B from *N*. *benthamiana* infected with TBSV. Top two panels: western blot analysis of co-purified HA-tagged p33 (lanes 2–3) with Flag-affinity purified Flag-AtFis1A or Flag-AtFis1B. P33-HA was detected with anti-HA antibody, while Flag-AtFis1A/B were detected with anti-Flag antibody as shown. Bottom two panels: western blot of total p33-HA and Flag-AtFis1A/B in the total plant extracts. (E) Pull-down assay including His_6_-Fis1-ΔTM (missing the C-terminal 25 aa), AtFis1A-ΔTM or AtFis1B-ΔTM and either the MBP-tagged TBSV p33 or CIRV p36 replication proteins. Note that we used the soluble C-terminal region of TBSV p33 or CIRV p36 replication proteins, which lacked the N-terminal TM domain. Top panel: western blot analysis of the captured Fis1-ΔTM, AtFis1A-ΔTM or AtFis1B-ΔTM with the MBP-affinity purified p33/p36 was performed with anti-His antibody. The negative control was the MBP (lanes 1, 4 and 7). Middle panel: Coomassie-blue stained SDS-PAGE of the captured MBP-p33, MBP-p36 and MBP. Bottom panels: western blot analysis of Fis1-ΔTM, AtFis1A-ΔTM or AtFis1B-ΔTM in total extracts. Coomassie-blue stained SDS-PAGE of the MBP-p33, MBP-p36 and MBP in total extracts. Each experiment was repeated three times. (F) Decreasing level of co-purification of Fis1p with the viral replicase after blocking new VRC assembly. The yeast samples were collected at the shown time points after the addition of cycloheximide (blocks cellular translation, thus new VRC formation) to the yeast culture. Note that samples were from yeasts replicating TBSV repRNA. Top panel: western blot analysis of co-purified His_6_-Fis1p with Flag-affinity purified Flag-p33 and Flag-p92^pol^ from membrane fraction of yeast. His_6_-Fis1p was detected with anti-His antibody. The negative control was His_6_-p33 and His_6_-p92^pol^ purified from yeast extracts using a Flag-affinity column. Middle panel: western blot of purified Flag-p33 detected with anti-Flag antibody. Bottom panels: western blots of His_6_-Fis1p, His_6_-p33 (lane 1) and Flag-p33 proteins in the total yeast extracts using anti-His and anti-Flag antibodies. The graph shows the % of co-purified His_6_-Fis1p with the tombusviral replication proteins with standard deviation. Each experiment was repeated three times. (G) Confocal laser microscopy images show the co-localization of TBSV BFP-tagged p33 replication protein with the YFP-tagged Fis1p protein and Pex13-RFP (peroxisomal marker) in WT and fis1Δ yeast cells. DIC (differential interference contrast) images are shown on the right.

To test if Fis1p could interact with the tombusvirus replication proteins, we used the split-ubiquitin-based membrane yeast two-hybrid assay (MYTH) [68]. We found that the yeast Fis1p and the homologous *Arabidopsis* Fis1A and Fis1B proteins interacted with the TBSV p33 replication protein (Fig 3A). We observed that separate expression of AtFis1A and AtFis1B proteins in fis1Δ yeast enhanced TBSV repRNA replication by ~2-fold (Fig 1C), indicating that the plant Fis1 proteins could provide pro-TBSV function in yeast lacking the yeast Fis1p. The interaction between these Fis1 proteins and p33 takes place in the membranous compartment based on purification of the Flag-tagged p33 and p92 replication proteins from detergent-solubilized membranous fraction of yeast that results in co-purification of Fis1p, AtFis1A and AtFis1B proteins (Fig 3B). Similar co-purification experiments with the mitochondrial Flag-tagged p36 and p95 replication proteins of CIRV from detergent-solubilized membranous fraction of yeast also confirmed the interaction of the replication proteins with Fis1p, AtFis1A and AtFis1B proteins (Fig 3C). We also found that the purified Flag-tagged AtFis1B protein, and to a lesser extent, Flag-AtFis1A preparations obtained from *N*. *benthamiana* plants expressing HA-tagged TBSV p33 contained the co-purified p33 replication protein (Fig 3D), suggesting that the interaction between AtFis1A and AtFis1B proteins and p33 replication protein also occurs in plant cells.

To confirm direct interactions between TBSV p33 and Fis1p, AtFis1A and AtFis1B proteins, we used a pull-down assay with MBP-tagged p33 and GST-6xHis-tagged Fis1p, AtFis1A and AtFis1B proteins from *E*. *coli* (Fig 3E). Similar interaction results were obtained with the pull-down assay based on the CIRV p36 replication protein (Fig 3E). For the pull-down assay, we used truncated TBSV p33 and CIRV p36 replication proteins and Fis1p, AtFis1A and AtFis1B proteins missing their corresponding membrane-binding regions to aid their solubility in *E*. *coli* (Fig 3E). Altogether, these data suggest that the interactions between the replication proteins of TBSV and CIRV and Fis1p, AtFis1A and AtFis1B host proteins occur within their domains facing the cytosolic compartment.

We were also curious if Fis1p was co-opted as a permanent component of the tombusvirus replicase. We halted the formation of new tombusvirus replicase complexes by stopping ribosomal translation through adding cycloheximide to the yeast growth media [69]. Then, we performed Flag-affinity-purification of the tombusvirus replicase from the membrane fraction of yeast at various time-points, which showed continuously decreasing amounts of the co-purified Fis1p in the purified replicase preparations (Fig 3F, lanes 3–4 versus 2). These observations suggest that Fis1p is released from the replicase, likely before the final assembly of the viral replicase. We suggest that the function of Fis1p with the replication proteins is temporal during the early steps of tombusvirus replication.

**Fig 4:**
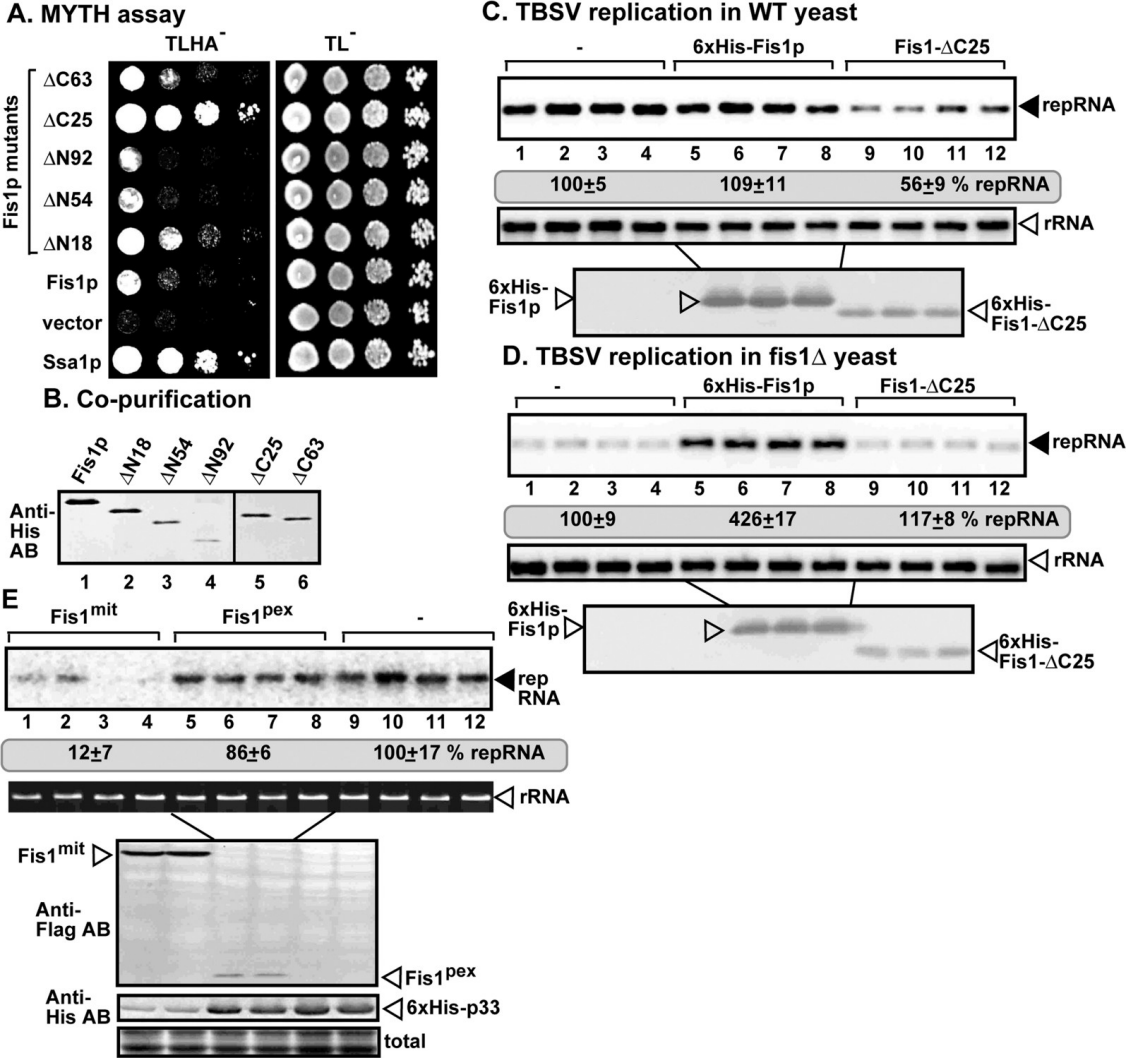
Fis1p lacking the TM domain is a dominant-negative inhibitor of TBSV replication in yeast. (A) The split ubiquitin-based MYTH assay was used to test binding between TBSV p33 and various N-terminal or C-terminal deletion mutants of the yeast Fis1p in yeast. The bait p33 was co-expressed with the shown prey proteins. Ssa1p and the empty prey vector (NubG) were used as positive and negative controls, respectively. The left panel shows p33: Fis1 interactions, the right panel demonstrates that comparable amounts of yeasts were used for these experiments. (B) Co-purification of various N-terminal or C-terminal deletion mutants of the yeast His_6_-Fis1p with Flag-p33 and Flag-p92^pol^ replication proteins from subcellular membranes. Western blot analysis of co-purified His_6_-Fis1p using anti-His antibody. (C-D) Expression of Fis1-ΔC25 inhibits TBSV replication in WT yeast. Note that Fis1 and Fis1-ΔC25 were expressed from the *CUP1* promoter. Top panel: northern blot analyses of TBSV repRNA using a 3’ end specific probe. Second panel: northern blot with 18S ribosomal RNA specific probe was used as a loading control. Bottom image: western blot analysis of the level of His_6_-tagged proteins with anti-His antibody. (E) Expression of Fis1^mit^ inhibits TBSV replication in WT yeast. Note that Fis1^mit^ carried a strong nitochondrial-membrane targeting signal, whereas Fis1^pex^ contained the peroxisomal membrane-targeting signal from Pex15. These Fis1 mutants were expressed from the *TEF1* promoter. Top panel: northern blot analyses of TBSV repRNA using a 3’ end specific probe. Second panel: Ethidium-bromide-stained agarose gel showing 18S ribosomal RNA as a loading control. Bottom image: western blot analysis of the level of Flag- or His_6_-tagged proteins with anti-Flag or anti-His antibodies.

To test if membrane-association of Fis1p or interaction of Fis1p with the TBSV p33 is important for TBSV replication, we used truncation mutants of Fis1p. The MYTH assay revealed that deletion of 54 or 92 N-terminal amino acids from Fis1p greatly reduced the interaction with p33 replication protein (Fig 4A). The weaker interaction between these Fis1p mutants and p33 replication protein was confirmed via co-purification experiments based on affinity-purification of Flag-p33 from detergent-solubilized membrane fraction of yeast (Fig 4B, lanes 3–4). This N-terminal region of Fis1p contains a TPR-region [57]. We have shown previously that p33 replication protein interacts with several TPR-domain containing host proteins [45,70].

Surprisingly, the mutant Fis1-ΔC25 with deletion of the C-terminal 25 amino acids, representing the transmembrane (TM) region [57], showed strong interaction with p33 replication protein in the MYTH assay (Fig 4A). The Flag-p33 affinity-purification experiments also showed efficient co-purification of Fis1-ΔC25 (Fig 4B), in spite of the low abundance of this truncated protein in yeast (Fig 4C and 4D, see the panels for western blot). Because deletion of the membrane-binding domain of Fis1p might result in a dominant-negative characteristic, we expressed Fis1-ΔC25 in WT yeast replicating TBSV repRNA. Indeed, we observed a ~2-fold reduced level of TBSV repRNA accumulation (Fig 4C lanes 9–12 versus 1–4), suggesting that Fis1-ΔC25 behaves as a dominant-negative mutant. Expression of Fis1-ΔC25 in fis1Δ yeast did not complement TBSV repRNA accumulation (Fig 4D lanes 9–12 versus 1–4). Because the cytosolic domain of Fis1p is known to form dimers and oligomers [56,57], these data are consistent with the model that Fis1-ΔC25 mutant might show a dominant-negative effect by inhibiting the wt Fis1p function. In addition, targeting of Fis1p to the mitochondrial membranes using a strong mitochondrium-membrane targeting signal fused to Fis1 [52] to reduce the presence of Fis1 in the peroxisomal membranes resulted in a dominant-negative effect on TBSV replication (Fis1^mit^ mutant, Fig 4E). Thus, all these data suggest that the subcellular location of Fis1 is important for TBSV replication. Expression of the N-terminally-truncated Fis1p mutant lacking the complete TPR-domain, which interacts with the p33 replication protein (Fig 4A), in fis1Δ yeast did not complement TBSV repRNA accumulation (Fis1-ΔN54, S2 Fig), suggesting that this mutant is not a functional mutant for pro-viral activities.

### Fis1 protein is recruited into the tombusvirus replication compartment in yeast and plants

To further analyze if Fis1p is co-opted by TBSV for supporting its replication, we co-expressed YFP-tagged Fis1p with BFP-tagged TBSV p33 replication protein and RFP-tagged Pex13 peroxisomal membrane protein in wt yeast cells, followed by confocal imaging. We found a high level of co-localization of TBSV p33 replication protein and Fis1p within the replication compartments consisting of aggregated peroxisomes (Fig 3G). The expression of p33 replication protein alone was enough to recruit Fis1p to a comparable extent as the actively replicating TBSV (Fig 3G). In the absence of viral components Fis1p only partially localized to peroxisomes in yeast (S3A Fig). Based on these experiments, we conclude that Fis1p is efficiently recruited by tombusvirus replication proteins to the tombusvirus replication compartments in yeast.

**Fig 5:**
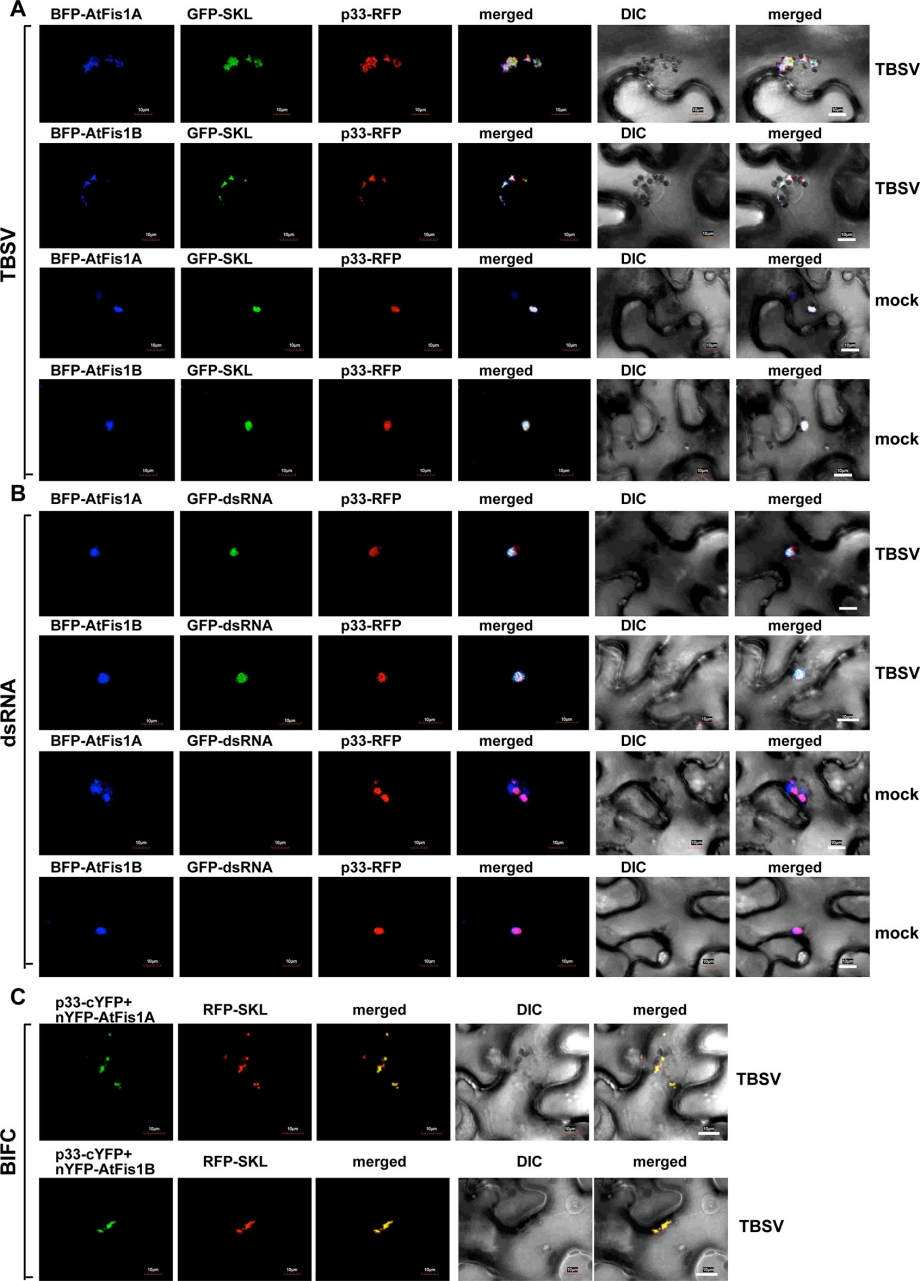
Recruitment of Fis1 by the TBSV p33 replication protein into the viral replication compartment in *N*. *benthamiana*. (A) Confocal microscopy images show efficient co-localization of TBSV p33-RFP replication protein and the BFP-AtFis1A/B, two plant orthologs of the yeast Fis1p within the viral replication compartment, marked by GFP-SKL peroxisomal marker *in N*. *benthamiana* leaves. Expression of these proteins from the 35S promoter was done after co-agroinfiltration into *N*. *benthamiana* leaves. The plant leaves were either TBSV-infected or mock-inoculated as shown. Scale bars represent 10 μm. (B) Co-localization of the viral double-stranded RNA replication intermediate with AtFis1A/B in *N*. *benthamiana* leaves infected with TBSV. The TBSV dsRNA was detected via a dsRNA detector assay based on dsRNA binding-dependent fluorescence complementation. The assay is based on expression of two dsRNA binding proteins, which are fused separately to N- and C-terminal halves of the yellow fluorescence protein (YFP). Simultaneous binding of the two fusion proteins to the same TBSV dsRNA leads to the restoration of YFP fluorescence, which could be visualized by confocal microscopy. Top panel: viral dsRNA is co-localized with BFP-AtFis1A within the replication compartment, which is marked by p33-RFP. No dsRNA signal was observed in mock-inoculated leaves. Expression of the above proteins from 35S promoter was done after co-agroinfiltration into *N*. *benthamiana* leaves. Scale bars represent 10 μm. Each experiment was repeated. (C) Interactions between TBSV p33-cYFP replication protein and the nYFP-AtFis1A/B proteins were detected by BiFC. The merged images show the efficient co-localization of RFP-SKL with the BiFC signal, indicating that the interactions between p33 replication protein and AtFis1A/B occur in the large viral replication compartments.

To confirm that Fis1 is also recruited by tombusviruses in the native plant cells, we co-expressed TBSV p33-RFP with the *Arabidopsis* BFP-tagged AtFis1A and GFP-SKL (peroxisomal luminar marker protein) in *N*. *benthamiana* leaves infected with TBSV. Confocal microscopy imaging revealed the extensive co-localization of AtFis1A with p33-RFP and GFP-SKL (Fig 5A), suggesting that AtFis1A is recruited into the large TBSV replication compartment in plant cells. Similar to yeast, we found that expression of the TBSV p33 replication protein alone (in the absence of viral replication) was enough to recruit AtFis1A (Fig 5A). The highly homologous AtFis1B is also co-localized with TBSV p33 during TBSV infection (Fig 5A). In the absence of TBSV replication, AtFis1B or AtFis1A only partially localized to peroxisomes when expressed in *N*. *benthamiana* (S4A and S4B Fig).

**Fig 6:**
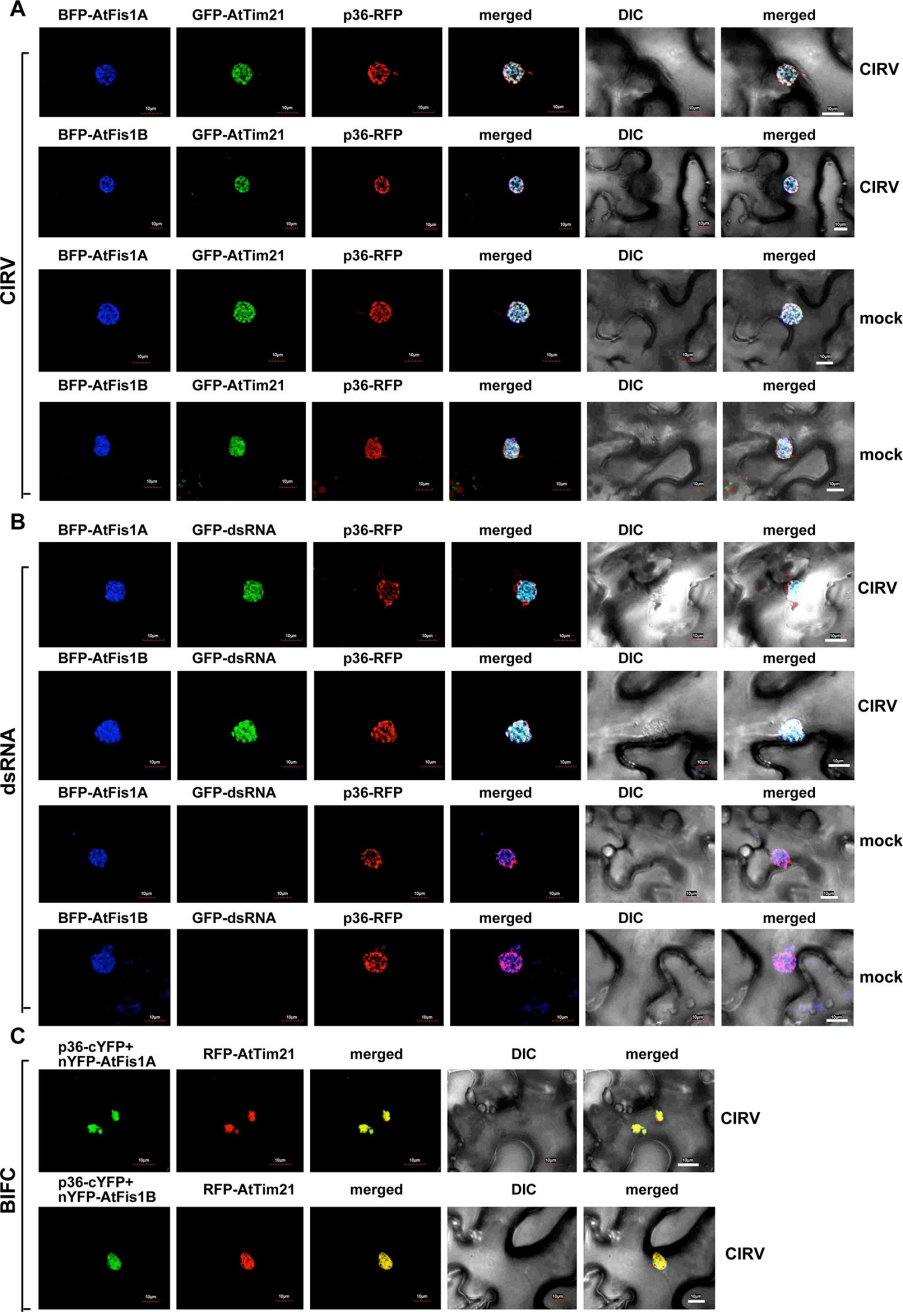
Recruitment of Fis1 by the CIRV p36 replication protein into the mitochondria-derived viral replication compartment in *N*. *benthamiana*. (A) Confocal microscopy images show efficient co-localization of CIRV p36-RFP replication protein and the BFP-AtFis1A/B within the viral replication compartment, marked by GFP-AtTim21 mitochondrial marker *in N*. *benthamiana* leaves. See further details in Fig 5A. (B) Co-localization of the CIRV dsRNA replication intermediate with AtFis1A/B in *N*. *benthamiana* leaves infected with CIRV. The CIRV dsRNA was detected via a dsRNA detector assay as described in Fig 5B. See further details in Fig 5B. (C) Interactions between CIRV p36-cYFP replication protein and the nYFP-AtFis1A/B protein was detected by BiFC. The merged images show the efficient co-localization of RFP-AtTim21 with the BiFC signal, indicating that the interactions between p33 replication protein and AtFis1A/B occur in the large viral replication compartments consisting of aggregated mitochondria.

Similar confocal microscopy-based experiments with the closely-related CIRV, which, unlike TBSV, builds the replication compartment from aggregated mitochondria, revealed the high extent of co-localization of AtFis1A/B with CIRV p36-RFP and GFP-AtTim21 mitochondrial membrane protein (Fig 6A). In the absence of CIRV replication, AtFis1B or AtFis1A only partially localized to mitochondria when expressed in *N*. *benthamiana* (S5 Fig). These data suggest that AtFis1A/B are recruited into the large CIRV replication compartment in plant cells.

To show that the recruitment of AtFis1A/B takes place in the active TBSV replication compartment (consisting of aggregated peroxisomes) or in the CIRV replication compartment (consisting of aggregated mitochondria) [71], we utilized a dsRNA sensor, which can detect the dsRNA replication intermediates during TBSV and CIRV replication [72]. Co-expression of the TBSV p33-RFP (or the CIRV p36-RFP) with the BFP-tagged AtFis1A/B and the dsRNA sensor (detected via GFP channel, see M&M) revealed the localization of AtFis1A and AtFis1B to the active TBSV and CIRV replication compartments containing the dsRNA replication intermediates (Figs 5B and 6B). We also found that the replicating (+)repRNA was mostly co-localized with AtFis1B in the replication compartment decorated with p33-BFP in *N*. *benthamiana* replicating CNV (S6 Fig). Therefore, Fis1 likely plays a role in the formation of the tombusvirus replication compartments.

To provide additional evidence that the plant Fis1A/B are recruited into the viral replication compartments through the interactions with the TBSV p33 or CIRV p36 replication proteins, we have conducted BiFC experiments with p33/p36 and AtFis1A/B in *N*. *benthamiana* leaves. The BiFC signals revealed specific interactions between AtFis1A/B and p33/p36 replication proteins within the replication compartment (Figs 5C and 6C, see also S7 Fig for the negative control experiments).

### Over-expression of the dynamin-related GTPase Dnm1 protein inhibits tombusvirus replication in yeast

**Fig 7:**
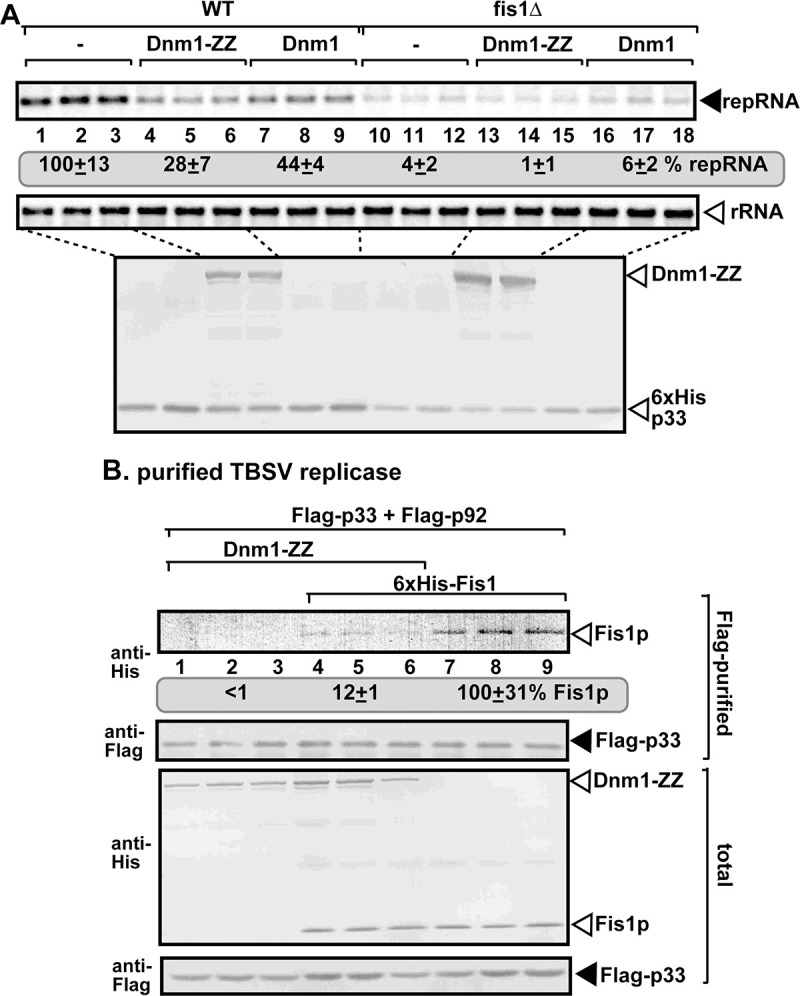
Over-expression of Dynamin-like GTPase Dnm1p inhibits tombusvirus replication in yeasts. (A) Expression of yeast Dnm1p, which interacts with Fis1p during fission events, inhibits TBSV replication in yeast. Top panel: northern blot analysis of TBSV repRNA using a 3’ end specific probe shows reduced accumulation of repRNA in WT yeast strain over-expressing Dnm1p (either ZZ-tagged or native), whereas Dnm1p over-expression has no effect on repRNA accumulation in fis1Δ yeast. Viral proteins His_6_-p33 and His_6_-p92^pol^ were expressed from plasmids from the *GAL1* promoter, while DI-72(+) repRNA was expressed from the *GAL10* promoter. Middle panel: northern blot with 18S ribosomal RNA specific probe was used as a loading control. Bottom images: western blot analysis of the level of His_6_-p33 and His_6_-p92^pol^ and ZZ-tagged Dnm1p with anti-His antibody. (B) Over-expression of Dnm1-ZZ reduces the amount of co-purified Fis1p in the tombusvirus replicase complex in WT yeast. Top panel: western blot analysis of co-purified His_6_-Fis1p with Flag-affinity purified Flag-p33 and Flag-p92^pol^ from membrane fraction of yeast. His_6_-Fis1p and Dnm1-ZZ were detected with anti-His antibody. Middle panel: western blot of purified Flag-p33 detected with anti-Flag antibody. Bottom panels: western blots of His_6_-Fis1p and Dnm1-ZZ proteins in the total yeast extracts using anti-His antibody. Each experiment was repeated three times.

To understand the functions provided by Fis1p for tombusvirus replication, we looked further into the canonical role of Fis1p in cells, which is to help assembling the mitochondrial and peroxisomal fission complexes [56]. We over-expressed the Fis1-interacting Dnm1p in yeast, which led to a reduced level of TBSV repRNA accumulation by ~2-to-4-fold (Fig 7A). Moreover, affinity-purification of Flag-p33 from detergent-solubilized membrane fraction of yeast revealed an ~8-fold reduction in co-purification of Fis1p when Dnm1p was also co-expressed (Fig 7B). Altogether, these results support the concept that the canonical function of Fis1p within the mitochondrial and peroxisomal fission complexes likely competes with the pro-viral function of Fis1p.

### Interaction between the host Fis1 protein and VAP protein facilitates tombusvirus replication

Since the canonical function of Fis1p likely competes with the pro-tombusviral function, we hypothesised that tombusviruses might be able to exploit the tethering function of Fis1p in peroxisomal and mithochondrial membranes [60,61]. One major intracellular structure exploited by tombusviruses, which requires extensive membrane tethering, is virus-induced membrane contact sites (vMCSs). The virus-induced and stabilized vMCSs are the places, where the ER membrane and the peroxisomal membrane come together in close proximity. vMCSs facilitates transfer of lipids, including sterols and PI(4)P phosphoinositide, and possibly host proteins to enrich the viral replication compartment and is needed for the formation of VRCs [35,40,73].

**Fig 8:**
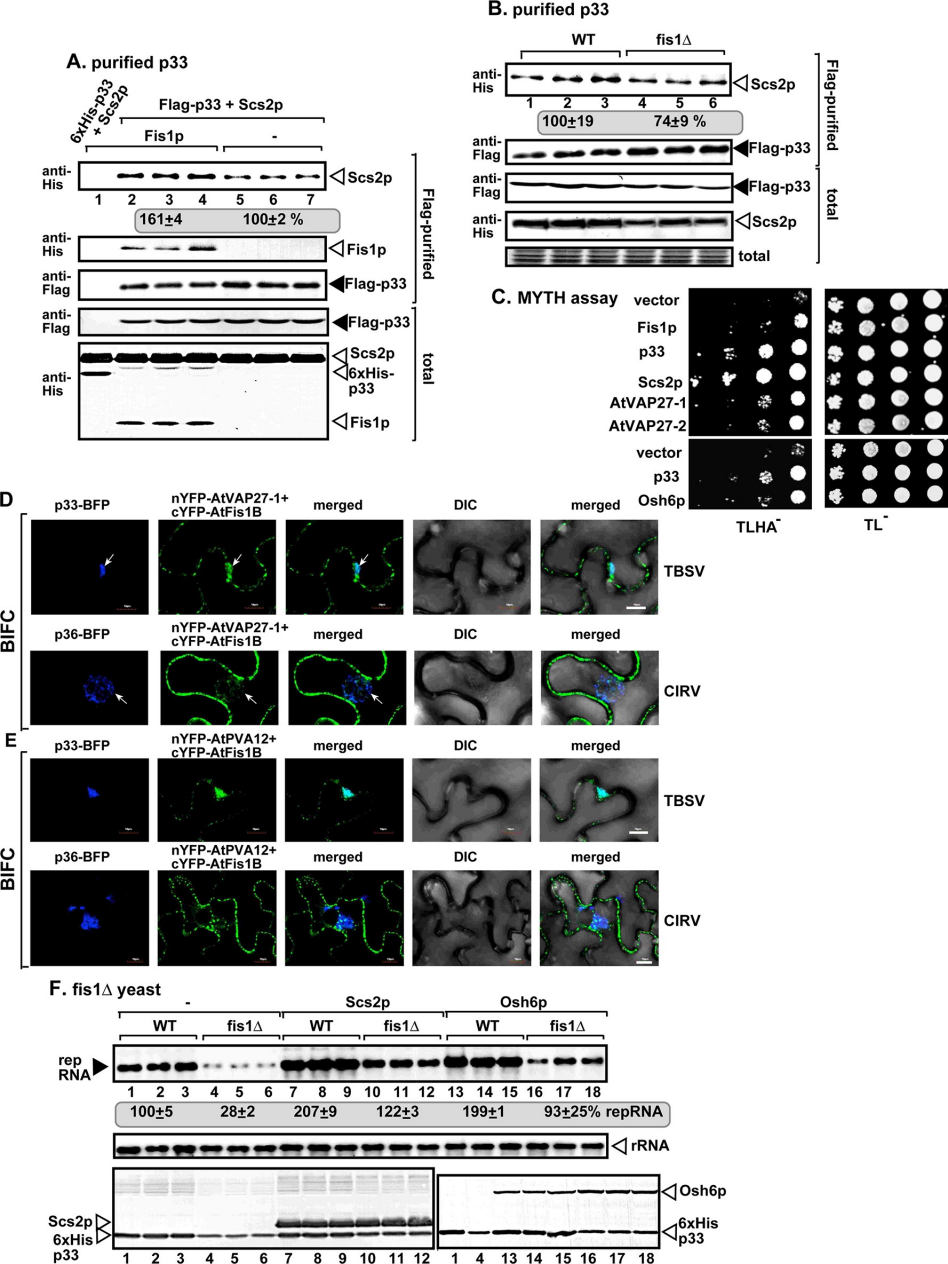
Fis1 facilitates the recruitment of the cellular ER-resident VAP protein into tombusvirus replication compartment. (A) Co-purification of the yeast His_6_-Scs2p VAP protein with TBSV Flag-p33 and Flag-p92^pol^ replication proteins from subcellular membranes of yeast over-expressing Fis1p. Top two panels: western blot analysis of co-purified His_6_-Scs2p detected with anti-His antibody, while Flag-p33 was detected with anti-Flag antibody. The negative control was from yeast expressing His_6_-p33 purified in a Flag-affinity column (lane 1). Samples were cross-linked with formaldehyde. Bottom two panels: western blot of total His_6_-Scs2p and Flag-p33 in the total yeast extracts. (B) Co-purification of the yeast His_6_-Scs2p with TBSV Flag-p33 and Flag-p92^pol^ replication proteins from subcellular membranes of WT or fis1Δ yeasts. See further details in panel A. (C) The split ubiquitin-based MYTH assay was used to test binding between the yeast Fis1p and Scs2p VAP or Osh6p or *Arabidopsis* AtVAP27-1/2 proteins in yeast. The bait Fis1p was co-expressed with the shown prey proteins. TBSV p33 and the empty prey vector (NubG) were used as positive and negative controls, respectively. The left panel shows the interactions, whereas the right panel demonstrates that comparable amounts of yeasts were used for these experiments. (D) Interactions between cYFP-AtFis1B and nYFP-AtVAP27-1 proteins within the TBSV p33-BFP or CIRV p36-BFP-decorated tombusvirus replication compartment were detected by BiFC. Expression of the above proteins from 35S promoter was done after co-agroinfiltration into *N*. *benthamiana* leaves infected with either TBSV or CIRV. Scale bars represent 10 μm. Each experiment was repeated. The arrows in the images show the large viral replication compartments consisting of aggregated peroxisomes/mitochondria. (E) Similar BiFC-based experiments with cYFP-AtFis1B and nYFP-AtPVA12 proteins within the TBSV p33-BFP or CIRV p36-BFP decorated replication compartment. See panel D for further details. (F) Complementation of *FIS1* deletion by over-expression of either Scs2p VAP protein or Osh6p oxysterol-binding protein in fis1Δ yeast. Northern and western blots with anti-His antibody were done as described in Fig 1A.

To test the putative role of Fis1p in vMCS formation, first we measured the amount of co-purified VAP protein, namely the ER-resident Scs2p VAP protein, which is a tethering protein required for vMCS formation [42]. We purified the tombusvirus replicase from WT yeast, followed by measuring the co-purified His_6_-Scs2p by western blotting. In comparison with the replicase preparations from wt yeast, the replicase preparations from WT yeast over-expressing Fis1p contained 60% more Scs2p (Fig 8A, compare lanes 2–4 and 5–7). On the contrary, the affinity-purified replicase preparations from fis1Δ yeast contained 25% less Scs2p than those obtained from WT yeast (Fig 8B). These observations suggest that the recruitment of Scs2p VAP protein to the viral replication compartment, likely to vMCSs, is affected by Fis1p.

To test if Fis1p interacts with Scs2p, we used the MYTH assay, which showed interaction even in the absence of viral p33 replication protein (Fig 8C). This assay also revealed interaction between Fis1p and the plant AtVAP27-1 and AtVAP27-2 proteins, which are the orthologs of the yeast Scs2p (Fig 8C). Based on these data, we suggest that the interaction between peroxisomal Fis1p and the ER-resident VAP proteins might help anchor vMCSs between the ER and peroxisomes.

To learn if Fis1-VAP interaction takes place within the viral replication compartment, we performed BIFC approach with AtVAP27-1 and AtFis1B and marked the replication compartment with TBSV p33-BFP or CIRV p36-BFP. Confocal microscopy revealed that AtVAP27-1 and AtFis1B interacts during TBSV and CIRV infection within the viral replication compartments (Fig 8D). Similar observations were made using AtPVA12 VAP protein, which is another plant ortholog of the yeast Scs2p, and AtFis1B with TBSV p33-BFP or CIRV p36-BFP (Fig 8D; see also S8 Fig for the negative control experiments). Therefore, we conclude that the plant VAP proteins, which are MCS tethering proteins [74], interact with Fis1 within the TBSV-induced peroxisomal and the CIRV-induced mitochondrial replication compartments. We also noted that similar to yeast, AtVAP27-1 and PVA12 VAP proteins interact with Fis1A/B (BiFC assay, S9 Fig) and partially co-localize with Fis1A and Fis1B in noninfected plant cells (S10A–S10D Fig).

Over-expression of the Scs2 VAP protein in fis1Δ yeast led to the complementation of Fis1p function based on restoration of TBSV repRNA accumulation to the level supported by WT yeast (Fig 8F). These data suggest that Fis1p putative tethering function (and/or scaffold function) can be complemented by providing abundant VAP proteins that could be hijacked by tombusviruses.

### Interaction between the host Fis1 protein and Sac1 PI4P phosphatase facilitates tombusvirus replication

Another critical host protein in vMCS formation/function is Sac1p PI4P phosphatase, which allows the directional transfer of sterols from ER to the acceptor membranes through converting PI(4)P phosphoinositide to PI phosphatidylinositol [35]. PI(4)P is used by oxysterol binding proteins (OSBP in mammals, Osh proteins in yeast, and ORP proteins in plants) to exchange for sterol/oxysterols/ergosterols to allow transfer of these lipids at the MCS [75–79].

**Fig 9:**
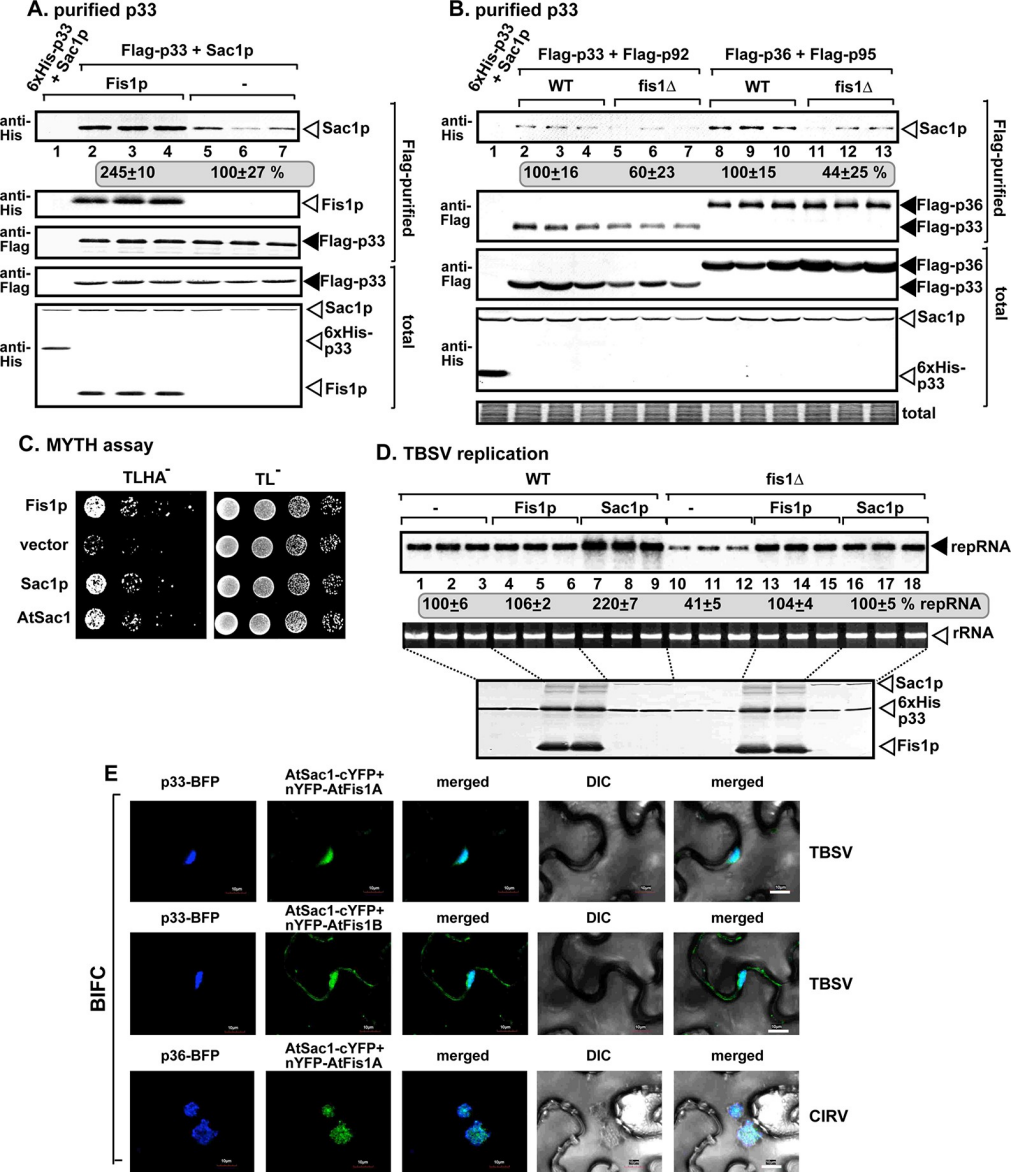
Fis1 facilitates the recruitment of the cellular Sac1 PI4P phosphatase protein into tombusvirus replication compartment. (A) Co-purification of the yeast His_6_-Sac1p with TBSV Flag-p33 and Flag-p92^pol^ replication proteins from subcellular membranes of yeast over-expressing Fis1p. Top two panels: western blot analysis of co-purified His_6_-Sac1p detected with anti-His antibody, while Flag-p33 was detected with anti-Flag antibody. The negative control was from yeast expressing His_6_-p33 purified in a Flag-affinity column (lane 1). Samples were cross-linked with formaldehyde. Bottom two panels: western blot of total His_6_-Sac1p and Flag-p33 in the total yeast extracts. (B) Co-purification of the yeast His_6_-Sac1p with either TBSV Flag-p33/Flag-p92^pol^ or CIRV Flag-p36/Flag-p95^pol^ replication proteins from subcellular membranes of WT or fis1Δ yeasts. See further details in panel A. (C) The split ubiquitin-based MYTH assay was used to test binding between the yeast Fis1p and Sac1p or *Arabidopsis* Sac1 protein in yeast. The bait Fis1p was co-expressed with the shown prey proteins. Fis1p and the empty prey vector (NubG) were used as positive and negative controls, respectively. The left panel shows the interactions, whereas the right panel demonstrates that comparable amounts of yeasts were used for these experiments. (D) Complementation of *FIS1* deletion by over-expression of Sac1p protein in fis1Δ yeast. Northern and western blots with anti-His antibody were done as described in Fig 1A. (E) Interactions between AtSac1-cYFP-and nYFP-AtFis1A/B proteins within the TBSV p33-BFP or CIRV p36-BFP-decorated tombusvirus replication compartment were detected by BiFC. Expression of the above proteins from 35S promoter was done after co-agroinfiltration into *N*. *benthamiana* leaves infected with either TBSV or CIRV. Scale bars represent 10 μm. Each experiment was repeated.

To test if Fis1p affects the recruitment of Sac1p to the viral replication compartment, we purified the tombusvirus replicase from WT yeast over-expressing Fis1p, followed by measuring the co-purified His_6_-Sac1p by western blotting. In comparison with the replicase preparations from WT yeast, the replicase preparations from WT yeast over-expressing Fis1p contained more than twice as much Sac1p than the preparations from WT yeast (Fig 9A, compare lanes 2–4 and 5–7). On the contrary, the affinity-purified replicase preparations from fis1Δ yeast contained ~50% less Sac1p than those obtained from WT yeast (Fig 9B). Based on these data, we suggest that the recruitment of the cellular Sac1p PI4P phosphatase to the viral vMCSs is greatly affected by Fis1p.

To test if Fis1p interacts with the yeast Sac1p, we used the MYTH assay, which showed interaction even in the absence of viral p33 replication protein (Fig 9C). This assay also revealed interaction between Fis1p and the plant AtSac1p protein, which is the ortholog of the yeast Sac1p (Fig 9C). These data suggest that the interaction between peroxisomal Fis1p and the ER-resident Sac1 PI4P phosphatase protein might help anchor vMCSs between the ER and peroxisomes.

To test if AtFis1A and AtFis1B interact with AtSac1, we used the BIFC assay in *N*. *benthamiana*, which showed interaction between these MCS proteins (Fig 9E). Interestingly, the interaction took place mostly in the VROs in TBSV or CIRV-infected plants (Fig 9E). These data support that the interaction between peroxisomal/mitochondrial Fis1 with Sac1 might help the formation of vMCSs. We also observed that Sac1 protein partially co-localized with Fis1A and Fis1B in noninfected plant cells (supplement S10E and S10F Fig).

Over-expression of the Sac1p in fis1Δ yeast led to the complementation of Fis1p function based on recovery of TBSV repRNA accumulation to the level supported by WT yeast (Fig 9D). Therefore, we suggest that when Sac1p is abundant in cells, then Sac1p could be easily recruited by the tombusvirus replication proteins even in the absence of Fis1p. However, when Sac1p is present at a normal level in WT yeast, then Fis1p is required for the efficient recruitment of Sac1p into the viral replication compartment for pro-viral functions.

### Interaction between the host Fis1 protein and Osh6 oxysterol-binding protein promotes tombusvirus replication

Previously we found that a group of oxystrerol-binding proteins (Osh proteins in yeast) are critical for vMCS formation/function and the enrichment of sterols within the viral replication compartment [42].

**Fig 10:**
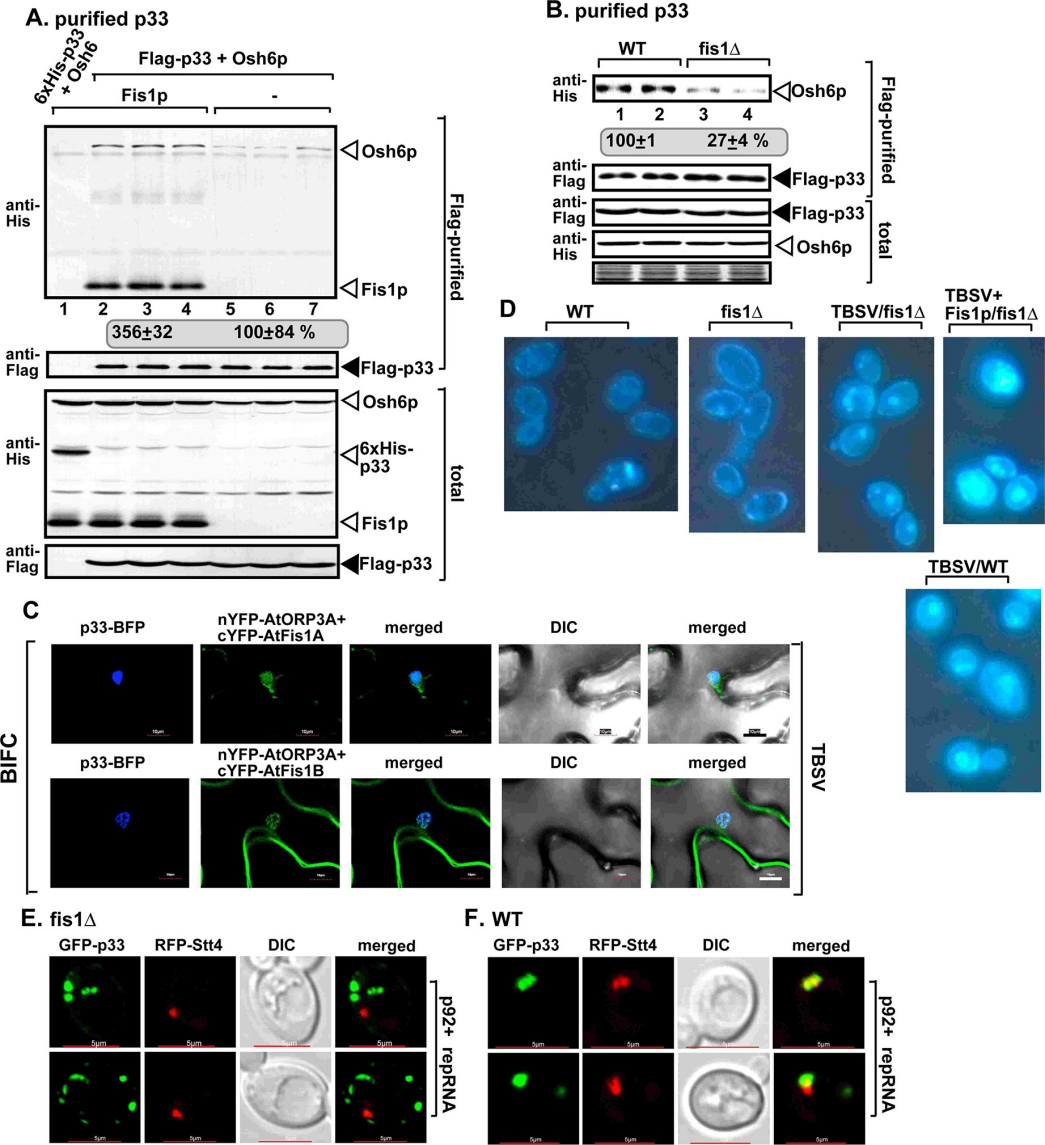
Recruitment of the cellular Osh/ORP proteins is facilitated by Fis1 into tombusvirus replication compartment. (A) Co-purification of the yeast His_6_-Osh6p with TBSV Flag-p33 and Flag-p92^pol^ replication proteins from subcellular membranes of yeast over-expressing Fis1p. Top two panels: western blot analysis of co-purified His_6_-Osh6p detected with anti-His antibody, while Flag-p33 was detected with anti-Flag antibody. The negative control was from yeast expressing His_6_-p33 purified in a Flag-affinity column (lane 1). Samples were cross-linked with formaldehyde. Bottom two panels: western blot of total His_6_-Osh6p and Flag-p33 in the total yeast extracts. (B) Co-purification of the yeast His_6_-Osh6p with TBSV Flag-p33 and Flag-p92^pol^ replication proteins from subcellular membranes of WT or fis1Δ yeasts. See further details in panel A. (C) Interactions between cYFP-AtFis1A/B and nYFP-AtORP3A proteins within the TBSV p33-BFP-decorated tombusvirus replication compartment were detected by BiFC. Expression of the above proteins from 35S promoter was done after co-agroinfiltration into *N*. *benthamiana* leaves infected with TBSV. Scale bars represent 10 μm. Each experiment was repeated. (D) Decreased level of re-localization of ergosterols to internal punctate structures in fis1Δ yeast replicating TBSV (third panel from left) in comparison with WT yeast (bottom panel) or fis1Δ yeast expressing Fis1p from a plasmid (right panel). Fluorescent microscopic images of yeast cells stained with the filipin dye. Note that filipin stains ergosterols present mostly at the plasma membrane in virus-free cells, as shown. (E-F) Confocal laser microscopy images show the lack of co-localization of TBSV GFP-tagged p33 replication protein with the RFP-tagged Stt4p PI4K kinase protein in fis1Δ and their co-localization in WT yeast cells, respectively. DIC and merged images are shown on the right. Note that yeasts co-expressed p92 and the (+)repRNA to support TBSV replication. Scale bars represent 5 μm. Each experiment was repeated.

The cytosolic Osh proteins must be recruited to vMCSs via protein-protein interactions [42]. To test the putative role of Fis1p in recruitment of Osh6p to the viral replication compartment, we purified the tombusvirus replicase from WT yeast over-expressing Fis1p, followed by measuring the co-purified His_6_-Osh6p by western blotting. The replicase preparations from WT yeast over-expressing Fis1p contained 3.5-fold more Osh6p than the similar preparations from WT yeast (Fig 10A, compare lanes 2–4 and 5–7). Deletion of *FIS1* gene in yeast resulted in affinity-purified replicase preparations, which contained ~4-fold less Osh6p than those obtained from WT yeast (Fig 10B). Thus, the recruitment of the cytosolic Osh6p to the viral vMCSs is greatly affected by Fis1p.

To test if Fis1p interacts with Osh6p, we used the MYTH assay. Interestingly, we observed interaction between Fis1p and Osh6p even in the absence of viral p33 replication protein (Fig 8C). The interaction between the homologous AtFis1A/B and AtORP3A (a plant orthologue of the yeast Osh proteins) was further confirmed with a BIFC assay in *N*. *benthamiana* (Fig 10C). The interaction between AtFis1A/B and AtORP3A took place mostly in the viral replication compartment in TBSV-infected plants (Fig 10C, see also S8 Fig for the negative control experiments). Altogether, these data strongly support that the interaction between peroxisomal/mitochondrial Fis1 with the cytosolic Osh/ORP proteins might help the recruitment of these lipid-transfer proteins to the virus-induced vMCSs.

Over-expression of the Osh6p in fis1Δ yeast resulted in the recovery of TBSV repRNA accumulation to the level supported by WT yeast (Fig 8E). Therefore, similar to the above MCS proteins, the abundant Osh6p could be easily recruited by the tombusvirus replication proteins in the absence of Fis1p. Yet, Fis1p facilitates the hijacking of Osh6p into pro-viral functions when Osh6p is present at a normal level in WT yeast where tombusviruses have to compete with normal cellular processes to subvert the host factors for pro-viral functions.

One of the major functions of the virus-induced vMCSs is to help the enrichment of sterols within the replication compartments [42]. Therefore, we tested the distribution of ergosterols (the sterol component in yeast) with fluorescent microscopy after staining the yeast cells with the filipin dye [80]. As expected [42], we found that TBSV replication resulted in redistribution of ergosterol mostly from the plasma membrane to internal locations (Fig 10D). *FIS1* deletion in yeast greatly reduced the internal ergosterols in the presence of the TBSV components, based on the much smaller-sized and dimmer lipid puncta (Fig 10D). Expression of Fis1p from a plasmid in fis1Δ yeast replicating TBSV restored the efficient redistribution of ergosterol to internal locations (Fig 10D). In comparison with WT yeast, *FIS1* deletion in yeast in the absence of the viral components did not result in major changes with most ergosterol accumulating in the plasma membrane (Fig 10D). These data strongly suggest that the expressed p33/p92 replication proteins in combination with the co-opted Fis1p stabilize the complexes containing Scs2p VAP, ORP and Sac1 proteins likely within vMCS. The stable vMCSs facilitate the efficient redistribution of sterols from the plasma membrane to internal locations, where it is incorporated into VRCs, which contain the sterol-binding p33 replication protein [29,42].

Another proposed important function of vMCS is to provide PI(4)P phosphoinositide for the OSBP-like oxysterol transfer proteins to drive the directional sterol exchange between the opposing membranes [75–79]. We have previously shown that TBSV co-opt the Stt4p PI4K kinase to VROs to produce PI(4)P, which is critical for VRO biogenesis [35]. To test if Fis1p affects the subcellular distribution of Stt4p PI4K during TBSV replication, we used fis1Δ yeast expressing the viral replication proteins. Interestingly, we observed the lack of Stt4p recruitment into p33-decorated VROs in fis1Δ yeast, in contrast with the situation in WT yeast that showed the partial relocalization of the PI4K into the VROs (Fig 10E and 10F). These observations suggest that Fis1p plays a role in the assembly or stabilization of vMCSs during TBSV replication.

**Fig 11:**
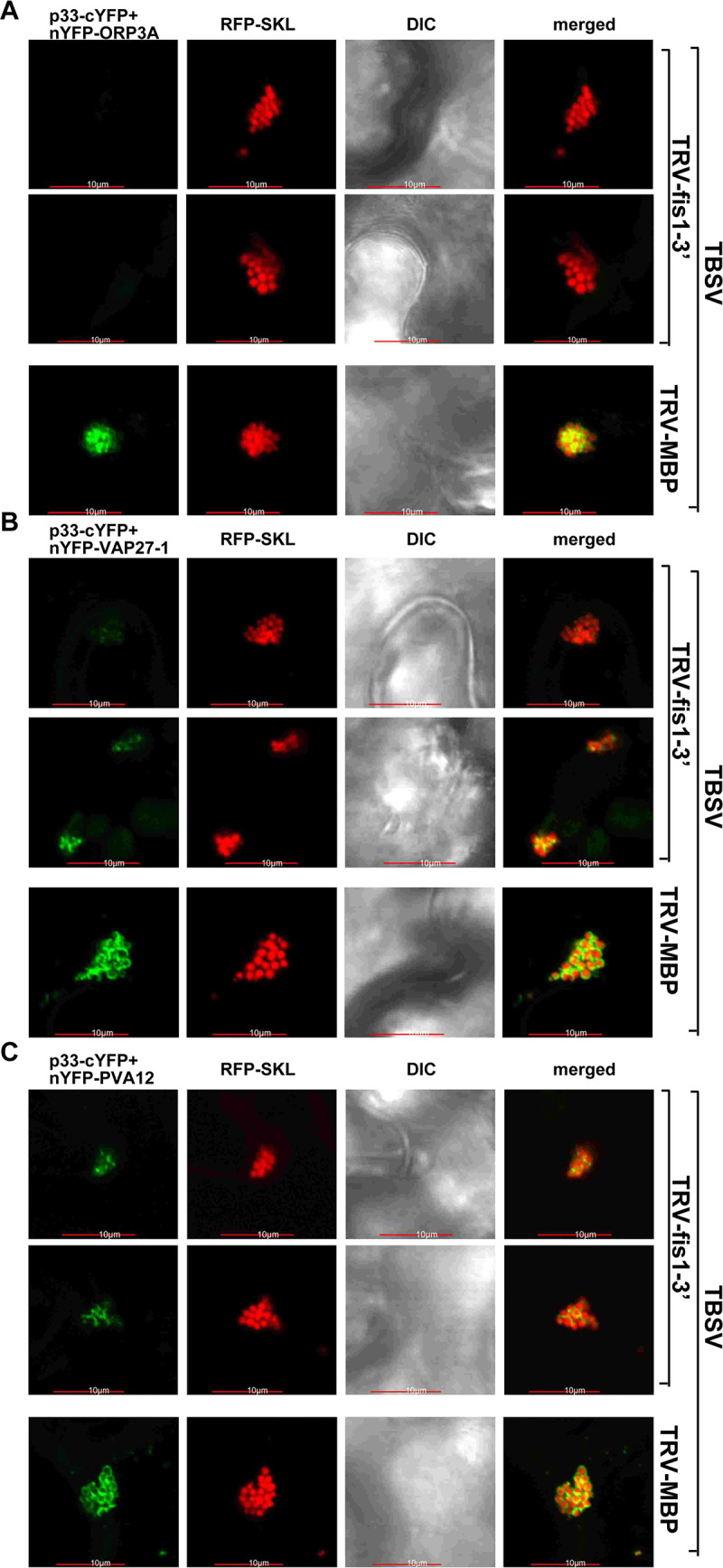
Silencing of Fis1 in *N*. *benthamiana* reduces the interaction between p33 replication protein and vMCS proteins within VROs. (A) Interactions between p33-cYFP and nYFP-AtORP3A proteins within the RFP-SKL-marked tombusvirus VROs representing the aggregated peroxisomes were detected by BiFC. Expression of the above proteins from 35S promoter was done after co-agroinfiltration into *N*. *benthamiana* leaves infected with TBSV. Top two panels represent cells with VIGS-based silencing of Fis1 (TRV-NbFis1 vector, see Fig 2), whereas the bottom panel is from control cell treated with TRV-MBP. Scale bars represent 10 μm. (B) Interactions between p33-cYFP and nYFP-AtVAP27-1 proteins within the RFP-SKL-marked tombusvirus VROs were detected by BiFC. See further details in panel A. (C) Interactions between p33-cYFP and nYFP-AtPVA12 VAP proteins within the RFP-SKL-marked tombusvirus VROs were detected by BiFC. See further details in panel A. Each experiment was repeated.

To test if Fis1 affects the vMCS formation via stabilizing protein-protein interactions among p33 and the vMCS proteins, we performed BiFC assays in *N*. *benthamiana* plants with VIGS-based Fis1 knockdown. Interestingly, p33 interaction with ORP3A oxysterol transfer protein was undetectable within the VROs in Fis1-silenced plant cells (Fig 11A). Moreover, the p33 interaction with the ER resident VAP27-1 and PVA12 VAP proteins was greatly diminished within the replication compartment in Fis1-silenced plant cells (Fig 11B and 11C, see also S11 Fig). This is in contrast with the strong interaction of p33 with ORP3A, VAP27-1 and PVA12 proteins within VROs in the control plants (Fig 11). Altogether, these data support the model that Fis1 protein, likely as a tethering factor, promotes the interaction between p33 and the vMCS proteins, which is proposed to lead to stabilization of vMCS structures within the VROs.

### Co-opting Fis1 protein is critical to build RNAi-insensitive tombusvirus replication compartment

**Fig 12:**
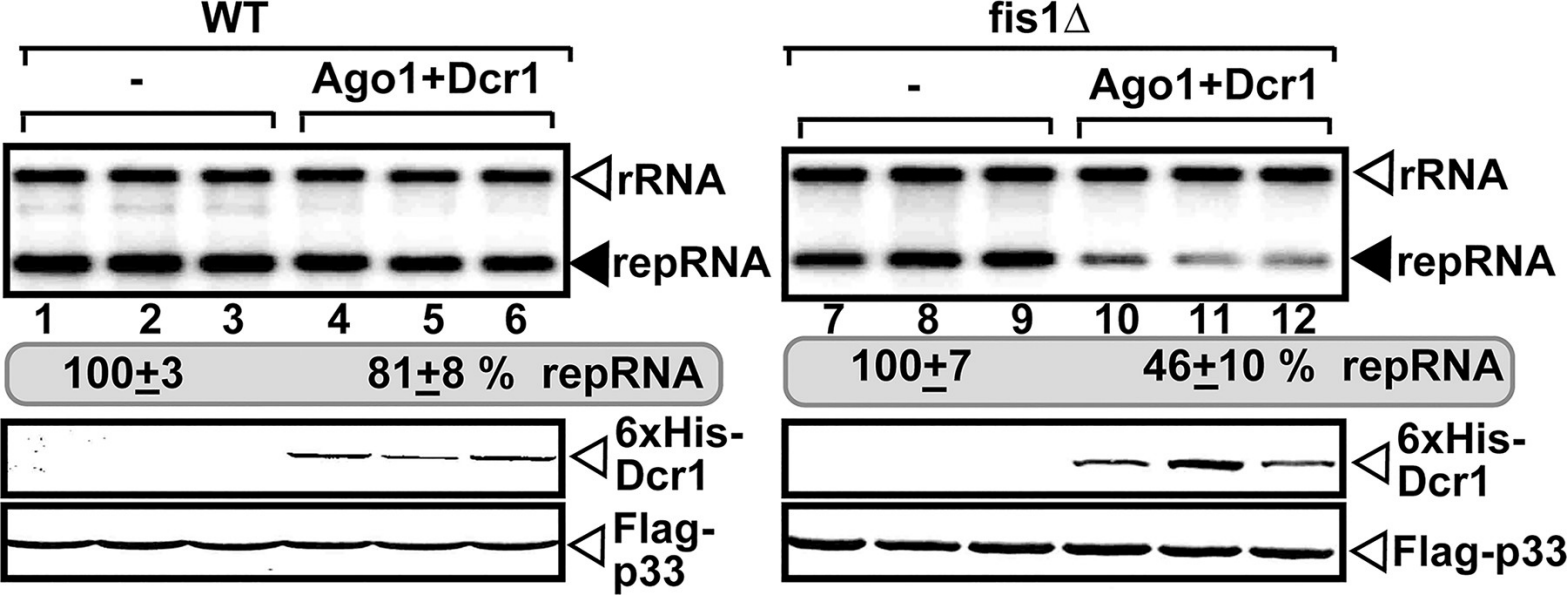
Deletion of *FIS1* sensitizes tombusvirus RNA to RNAi-based degradation in yeast. Co-expression of *S*. *castellii* AGO1 and DCR1 in fis1Δ yeast reduces TBSV repRNA accumulation more than in WT yeast (BY4741). Top panel: Replication of the TBSV repRNA was measured by northern blotting 24 h after initiation of TBSV replication. The accumulation level of repRNA was normalized based on the ribosomal (r)RNA. Note that the TBSV repRNA, p33 and p92 replication proteins were expressed from plasmids. Each sample is obtained from different yeast colonies. Yeast strain not expressing RNAi components is taken as 100% in each experiment. Average value and standard deviation is calculated from all the biological repeats. Middle and bottom panels: The accumulation levels of His_6_-DCR1 and Flag-p33 replication protein were tested by western blotting. Each experiment was repeated twice. Ribosomal RNA is shown as a loading control.

Previously, we have used the reconstituted RNAi machinery from *S*. *castellii* with the two-component *DCR1* and *AGO1* genes [81] to probe the contribution of co-opted host factors to the protection of the viral RNA [14]. Based on the reconstituted RNAi machinery in yeast, we measured TBSV accumulation when RNAi activity was induced in WT as well as fis1Δ yeasts. Deletion of *FIS1* gene in yeast led to poor protection of the TBSV RNA, resulting in ~50% reduction of repRNA in comparison with the yeast strain not expressing the RNAi components (Fig 12, lanes 10–12 versus lanes 7–9). As previously found, induction of the RNAi machinery in WT yeast had only minor effect on TBSV accumulation (Fig 12). Based on these data, we suggest that Fis1p is a major co-opted host factor needed for the biogenesis of the protective viral replication compartment.

We also tested Fis1-silenced plants for the accumulation of vsiRNAs. Unfortunately, the accumulation of TBSV (+) and (-)RNAs were so low in Fis1-silenced *N*. *benthamiana* plants that the generation of vsiRNAs was barely detectable in this setting (S12 Fig). This is in contrast with the control plants, which generated abundant vsiRNAs (S12 Fig). These vsiRNAs, however, are likely the combined results of the host RDR6/DCL2-4 activities, which efficiently amplify the secondary vsiRNAs. The primary vsiRNAs, which are generated on the dsRNA replication intermediates, are less abundant in plants [82–85].

## Discussion

### A novel tethering role of Fis1 mitochondrial fission protein in the formation of the dynamic vMCS to support tombusvirus replication

**Fig 13:**
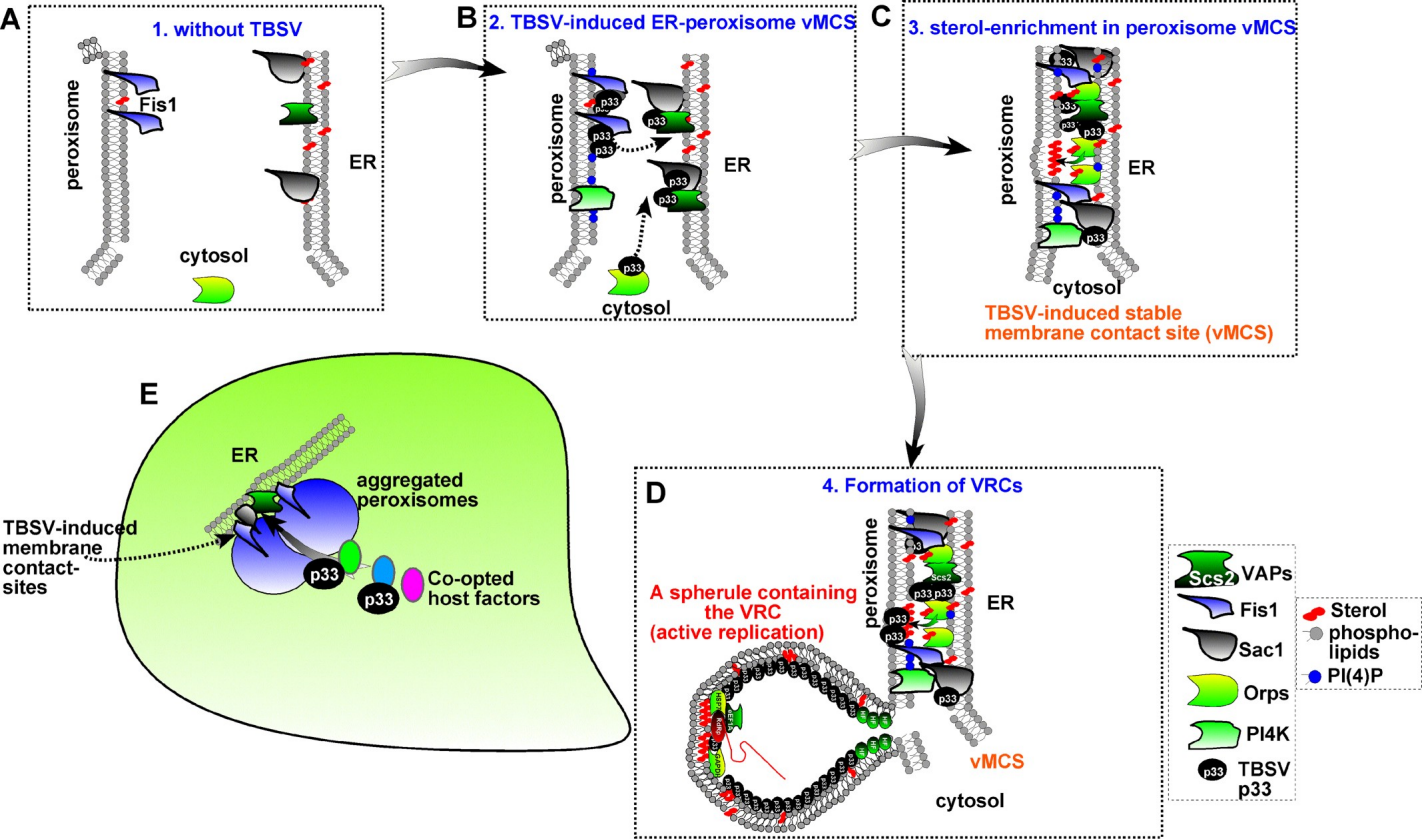
A model on the functional role of the Fis1 mitochondrial fission protein 1 in the formation of virus-induced membrane contact sites. (A) In the absence of tombusvirus replication, the role of Fis1 in MCS formation is undefined and needs to be investigated. (B) Expression of the abundant p33 replication protein of TBSV leads to p33 molecules-driven efficient recruitment of Fis1 to peroxisome membranes, likely with the assistance of the cellular Pex19 peroxisomal transport protein. P33 also binds to the ER-resident VAP tethering protein and Sac1 PI4P phosphatase and the cytosolic oxysterol-binding proteins (ORP). The cellular PI4K kinase is also recruited into vMCS. All these processes lead to the tethering of the peroxisomal membranes to the syntaxin 18-like Ufe1 and Use1 SNARE proteins-marked subdomains of the ER membrane, resulting in the formation and stabilization of vMCSs. (C) The interplay between the co-opted ORP proteins, PI(4)P phosphoinositide and Sac1 facilitates the enrichment of sterols and other lipids within the peroxisomal membranes. (D) These processes render the peroxisomal membranes highly suitable for the formation of the virus-induced spherules, which harbor the VRCs needed for virus replication. Overall, by hijacking these cellular components for pro-viral functions makes tombusviruses capable of building nuclease and protease-insensitive virus replication compartment. (E) A scheme of the TBSV-infected cell with the formation of vMCS and the replication compartment. Please note the related CIRV builds similar virus-induced structures utilizing the mitochondria, instead of peroxisomes, and the ER membranes.

Biogenesis of the large viral replication compartments and inside these entities, constructions of the individual spherules/VRCs are elaborate processes, which are orchestrated by the viral replication proteins [24,41,86]. In addition to co-opting numerous cellular proteins and various subcellular membranes, tombusviruses also induce major changes in lipid composition of the hijacked cellular membranes [30,35,36,42]. The emerging picture with tombusviruses and other unrelated human viruses, such as enteroviruses, that these viruses induce and stabilize vMCSs in order to manipulate the lipid composition of the membranes within the viral replication compartment [40,87]. While previously we have identified the critical roles of the ER-resident VAP tethering proteins, Sac1 PI4P phosphatase and the cytosolic OSBP/Osh/ORP oxysterol-binding proteins in the formation of vMCSs [35,40,42], it was not known how the peroxisomes in case of TBSV infection or the mitochondria in case of CIRV infection are recruited to form vMCSs with the ER membrane. In this work we provide several pieces of evidence that the highly conserved Fis1 mitochondrial fission protein, which is present on the outer surface of peroxisomal and mitochondrial membranes, seems to play the tethering function, likely in a complex with the p33 replication protein, for the peroxisomes and mitochondria during vMCSs formation. (i) The yeast Fis1p and the homologous plant AtFis1A/B proteins interact with the TBSV p33 and the CIRV p36 replication proteins and the interaction takes place within the viral replication compartment. (ii) No known enzymatic function has been found for Fis1 [56,88,89]. However, membrane localization of Fis1 is required for pro-viral function, because the deletion mutant lacking the tail-anchored region in Fis1 acted as a dominant-negative mutant by inhibiting TBSV replication in WT yeast (Fig 4C). (iii) Fis1 is temporally associated with the VRCs, thus likely affecting an early step during VRC formation (Fig 3F). (iv) Fis1 expression level affected the recruitment of other core vMCSs-localized proteins, such as the ER-resident VAP protein (Scs2 in yeast, VAP27-1/2 and PVA12 in plants), the ER-localized Sac1 PI4P phosphatase and the cytosolic Osh/ORP proteins (Figs 8–10). (v) Deletion of Fis1 in yeast inhibited the TBSV-induced accumulation of ergosterol in internal locations, instead of the plasma membrane localization in a TBSV-free yeast (Fig 10D). (vi) Deletion of Fis1 prevented the re-localization of PI4K into the VROs, which produces PI4P required for Osh and Sac1 functions in vMCS [35]. (vii) Fis1 is required to build tombusvirus replication compartment resilient to RNAi (Fig 12), which depends on the enrichment of sterols within VRC membranes [14]. (viii) The complementation experiments with over-expression of VAP proteins, Sac1p and Osh6p in fis1Δ yeasts suggest unexpectedly that the various co-opted vMCS tethering proteins provide redundant functions during stabilization of vMCS. This can be interpreted that the recruitment of these host factors by the p33 replication protein for vMCS formation is a strong rate-limiting step under normal conditions. In other words, providing abundant vMCS tethering proteins via over-expression enhances tombusvirus replication likely by assisting the subversion of these tethering proteins for viral functions by the p33 replication protein, due to less competition with normal cellular processes. Altogether, all these data strongly support the model that tombusviruses induce vMCSs by co-opting the tethering function of Fis1, which in turn helps TBSV to recruit the peroxisomal and CIRV to recruit the mitochondrial membranes to participate in vMCSs formation. Based on subcellular localization, it seems that Fis1 acts as a tether from the peroxisome and mitochondria sides, whereas the subverted Sac1 and VAP tethering proteins act from the ER side during vMCS formation/function (Fig 13). However, the entire process is orchestrated by the TBSV p33 or CIRV p36 replication proteins through binding to these co-opted host factors, including Osh/ORP proteins, too. These dynamic interactions within the vMCSs make it possible for tombusviruses to efficiently construct the individual VRCs within the large VROs in infected cells.

Why do tombusviruses need the help of these co-opted tethering proteins? The tombusvirus replication proteins interact with numerous cellular proteins with pro-viral as well as antiviral functions [45,90–92]. Therefore, tombusvirus replication proteins might be too promiscuous and not be suitable to build vMCSs with targeted organelles without the contribution of the specialized tethering host factors. We propose that by targeting and co-opting selected tethering proteins, such as the ER-resident Sac1 and VAP proteins and the peroxisomal and mitochondrial Fis1 protein, tombusviruses could efficiently build these dynamic and specialized vMCS structures. Moreover, by recruiting them away from their canonical cellular functions, the replication proteins could stabilize vMCSs to make the biogenesis of the replication compartment more robust and efficient. Altogether, this work unraveled how tombusviruses could exploit dynamic protein-protein interactions to build vMCSs and the viral replication compartments.

### Wide-spread roles of co-opted MCS tethering proteins in (+)RNA virus replication?

In this work, we obtained evidence for the role of Fis1 in replication of tombusviruses, a related carmovirus and an unrelated alfanodavirus of insects. Also, works on enteroviruses indicate the important role of vMCS and co-opted cellular Sac1, OSBP and VAP tethering proteins in building the viral replication compartment [87]. Therefore, it will be interesting to learn if other (+)RNA viruses exploit vMCS and tethering proteins for their replication. Targeting these organelle-tethering proteins by inhibitors or other ways could open up new antiviral strategies.

## Materials and methods

### Plant materials, yeast strain and plasmids

Wild type *N*. *benthamiana* plants were potted in soil and placed in a growth room at 25°C under a 16-h-light/8-h-dark cycle. *S*. *cerevisiae* strain BY4741 (MATa his3Δ1 leu2Δ0 met15Δ0 ura3Δ0) was purchased from Open Biosystems and stored in a -80°C refrigerator. Yeast strain fis1Δ was kindly provided by Dr. Agnes Delahodde (University of Paris-Sud) [51]. Yeast strains dnm1Δ, mdv1Δ, caf4Δ and vps1Δ were from YKO library (Openbiosystems). Yeast strain BY4741-ADH-Hisp92 was generated and described previously [42]. Yeast strain NMY51 was obtained from Dualsystems. Plasmids and their constructions are listed in S1 Table.

### Analysis of virus replication in yeast

To determine the effect of Fis1p on replication of different viruses in yeast, BY4741 and fis1Δ yeast strains were transformed with different combination of plasmids. For TBSV replication, yeast strains were transformed with HpGBK-Gal-HisT33/Gal-DI-72, LpGAD-Gal-HisT92 and UpYES-NT empty vector or UpYES-NT-HisScFis1. TBSV replication was induced by growing cells at 23°C in SC-ULH^−^ (synthetic complete medium without uracil, leucine and histidine) medium supplemented with 2% galactose for 24 h. For CNV and CIRV replication, plasmids HpGBK-CUP1-Hisp33/Gal-DI-72 and LpGAD-CUP1-Hisp92 or HpESC-CUP1-Hisp36/Gal-DI-72 and LpESC-CUP1-Hisp95, respectively, were co-transformed with UpYES-NT empty vector or UpYES-NT-HisScFis1 into yeast strains. Transformed yeast cells were pre-grown in 2 ml SC-ULH^−^ medium supplemented with 2% galactose and 100 μM BCS for 16 h at 23°C. Then yeast cultures were resuspended in SC-ULH^−^ medium supplemented with 2% galactose and 50 μM CuSO_4_ and grown for 24 h at 23°C. For FHV replication, yeast strains were transformed with pESC-His/Gal/FHV/RNA1/Frameshift/TRSVR_RZ_, pGAD-Leu/Cup/FHV/Protein-A/C-term/HA/Flag and UpYES-NT empty vector or UpYES-NT-HisScFis1. After pre-growing in 2 ml SC-ULH^−^ medium supplemented with 2% glucose and 100 μM BCS for 16 h at 29°C, yeast cells were resuspended in SC-ULH^−^ medium supplemented with 2% galactose and 50 μM CuSO_4_ and grown for 48 h at 29°C.

To study the effect of different deletion mutants of Fis1p on tombusvirus replication, BY4741 and fis1Δ yeast strains were transformed with HpGBK-CUP1-Hisp33/Gal-DI-72 and LpGAD-CUP1-His92 and one of the following: UpYES-NT, UpYES-HisScFis1, UpYES-HisScFis1ΔC25, UpYES-HisScFis1ΔN18 or UpYES-HisScFis1ΔN54. Transformed yeast cells were pre-grown in 2 ml SC-ULH^−^ medium supplemented with 2% galactose and 100 μM BCS for 16 h at 23°C. Then yeast cultures were resuspended in SC-ULH^−^ medium supplemented with 2% galactose and 50 μM CuSO_4_ and cultured for 24 h at 23°C.

To test TBSV replication upon expression of re-targeted Fis1 in WT yeast, we introduced pYes-6xHis-p92, pEsc-6xHis-p33-Gal-DI72 with pRS315-EV or pRS315-Flag-Fis1-pex15 or pRS315-Flag-p36mts-Fis1 into wt yeast. The transformed yeast cells were pre-grown in synthetic complete medium (ULH^-^) supplemented with 2% glucose at 29°C for overnight, then repRNA replication was induced by providing the yeast to synthetic complete medium ULH^-^ supplemented with 2% galactose at 23°C for 24 h. Yeast cells were collected for protein and RNA analysis.

To overexpress other membrane contact sites-associated proteins in the presence or absence of Fis1 during tombusvirus replication, yeast strains BY4741 and fis1Δ were transformed with HpGBK-CUP1-Hisp33/Gal-DI-72, LpGAD-CUP1-His92 and one of the following plasmids UpYC-ScOsh6, UpYC-ScScs2 or UpYC-ScSac1. Transformed yeast cells were pre-grown in 2 ml SC-ULH^−^ medium supplemented with 2% galactose and 100 μM BCS for 16 h at 23°C. Then yeast cultures were resuspended in SC-ULH^−^ medium supplemented with 2% galactose and 50 μM CuSO_4_ and grown for 24 h at 23°C.

To study the critical function of Fis1p in sterol enrichment for building protective subcellular environment, induction of RNAi through co-expression of DCR1 and AGO1 in yeast replicating TBSV repRNA were performed as described [14]. Briefly, HpGBK-CUP1-Hisp33/Gal-DI-72 and LpGAD-CUP1-His92 were co-transformed with pESC-Ura or pESC-Ura-Gal10-HisDcr1 or pESC-Ura-Gal1-HisAgo1-Gal10-HisDcr1 into BY4741 and fis1Δ yeast strain. After pre-growing in 2 ml SC-ULH^−^ medium supplemented with 2% glucose and 100 μM BCS for 16 h at 29°C, yeast cultures were resuspended in SC-ULH^−^ medium supplemented with 2% galactose and 100 μM BCS and grown for 24 h at 23°C. Then, yeast cells were shifted to SC-ULH^−^ medium supplemented with 2% galactose and 50 μM CuSO_4_ and grown for 16h at 23°C.

Yeast stains BY4741 and fis1Δ transformed with HpGBK-CUP1-Hisp33/Gal-DI-72, LpGAD-CUP1-Hisp92 and UpBG1805-ScDnm1-ZZ or UpESC-ScDnm1 or UpESC-empty were applied to study the effect of Dnm1p on tombusvirus replication. Transformed yeast cells were pre-grown in 2 ml SC-ULH^−^ medium supplemented with 2% galactose and 100 μM BCS for 16 h at 23°C. Then yeast cultures were resuspended in SC-ULH^−^ medium supplemented with 2% galactose and 50 μM CuSO_4_ and grown for 24 h at 23°C. Meanwhile, plasmids HpGBK-CUP1-Hisp33/Gal-DI-72, LpGAD-HisScFis1 or LpESC-empty, and UpBG1805-ScDnm1-ZZ or UpESC-empty were transformed into BY4741-ADH-Hisp92 yeast strain, followed by same growing method mentioned above. All the replication assays in yeast were performed with a standard RNA and protein extraction protocol described in previous publications [93,94].

Additional materials and methods can be found in S1 Text and S1–S3 Tables in supporting information.

## Supporting information

S1 Fig Fis1p mitochondrial fission 1 protein is an essential host factor for tombusvirus and alfanodavirus replication in yeast. (A) Deletion of *FIS1* of the mitochondrial fission complex inhibits replication of TBSV in yeast. Top panel: northern blot analyses of repRNA using a 3’ end specific probe demonstrate reduced accumulation of repRNA in fis1Δ yeast strain in comparison with deletion of the other four genes that form the mitochondrial fission complex. Viral proteins His_6_-p33 and His_6_-p92 of TBSV were expressed from the galactose-inducible *GAL1* promoter, whereas the repRNA was expressed from the constitutive *ADH1* promoter. Second panel: Ethidium-bromide stained agarose gels show 18S ribosomal RNA as a loading control. Bottom images: western blot analysis of the level of His_6_-p33 and His_6_-p92 proteins with anti-His antibody. (B-C) Deletion of *FIS1* of the mitochondrial fission complex inhibits replication of FHV and NoV insect viruses in yeast. Northern blot analyses of RNA1 and the 3’-nested subgenomic RNA3 using a 3’ end specific probe demonstrate reduced accumulation of viral RNAs in fis1Δ yeast strain in comparison with deletion of the other four genes that form the mitochondrial fission complex. The FHV RNA1 was expressed from the *GAL1* promoter, whereas protein A replication protein was expressed from the *CUP1* promoter. In case of NoV, RNA1 was expressed from the *CUP1* promoter, whereas the NoV protein A replication protein was expressed from the *CUP1* promoter. Additional details can be found in Fig 1. (D) Over-expression of the yeast Fis1p from a plasmid enhances NoV RNA replication in yeast. Additional details can be found in Fig 1.
(TIF)

S2 Fig Over-expression of the N-terminal deletion mutants of Fis1p shows the lack of pro-viral function in fis1Δ yeast. Northern blot analyses of repRNA using a 3’ end specific probe demonstrates reduced accumulation of repRNA in fis1Δ yeast strain expressing the shown N-terminal deletion mutants in comparison with the WT Fis1p. Viral proteins His_6_-p33 and His_6_-p92 of TBSV were expressed from plasmids from the *GAL1* promoter, while DI-72(+) repRNA was expressed from the *GAL10* promoter. His_6_-Fis1p was expressed from the *GAL1* promoter from a plasmid. Second panel: Ethidium-bromide stained agarose gel shows 18S ribosomal RNA as a loading control.
(TIF)

S3 Fig Confocal laser microscopy images show the partial co-localization of the YFP-tagged Fis1p protein and Pex13-RFP (peroxisomal marker) in WT yeast cells in the absence of viral components. DIC images are shown on the right. See further details in Fig 3G.
(TIF)

S4 Fig Confocal laser microscopy images show the partial co-localization of the RFP-tagged AtFis1A and RFP-AtFis1B proteins and GFP-SKL (peroxisomal marker) in *N*. *benthamiana* in the absence or presence of TBSV infection. (A-B) See further details in Fig 5A.
(TIF)

S5 Fig Confocal laser microscopy images show the partial co-localization of the RFP-tagged AtFis1A and RFP-AtFis1B proteins and GFP-Tim21 (mitochondrial marker) in *N*. *benthamiana* in the absence of TBSV infection. See further details in Fig 6A.
(TIF)

S6 Fig Co-localization of the viral (+)repRNA replication products with AtFis1B in *N*. *benthamiana* leaves infected with CNV. The (+)repRNA carries 6 copies of the 19 nt long hairpin sequence from the MS2 phage, which is specifically recognized by the RFP-tagged MS2-CP (coat protein). Confocal microscopy images show the co-localization of the (+)repRNA with GFP-AtFis1B within the replication compartment, which is marked by p33-BFP. Expression of the above proteins and the repRNA was from 35S promoter via co-agroinfiltration into *N*. *benthamiana* leaves also infected with CNV. Scale bars represent 10 μm. Each experiment was repeated.
(TIF)

S7 Fig Control experiments for the BiFC assays. The lack of BiFC signals in these experiments indicates that the interactions between TBSV p33-cYFP and the CIRV p36-cYFP replication proteins and the nYFP-AtFis1A and nYFP-AtFis1B proteins are specific. See further details in Figs 5C and 6C.
(TIF)

S8 Fig Additional control experiments for the BiFC assays. The lack of BiFC signals in these experiments indicates that the interactions between nYFP-AtVAP27-1 and the nYFP-AtPVA12 VAP proteins and the cYFP-AtFis1A and cYFP-AtFis1B proteins are specific. See further details in Fig 8D. Similarly, the lack of BiFC signals in these experiments indicates that the interactions between the OSBP-like nYFP-AtORP3A protein and the cYFP-AtFis1A and cYFP-AtFis1B proteins are specific. See further details in Fig 10C.
(TIF)

S9 Fig Control experiments for the BiFC assays in the absence of TBSV replication. Interactions between cYFP-AtFis1B and nYFP-AtVAP27-1 or nYFP-AtPAV12 or nYFP-AtORP3A proteins were detected by BiFC. Also, interaction between nYFP-AtFis1B and AtSac1-cYFP was detected by BiFC in *N*. *benthamiana* leaves. Expression of the above proteins from 35S promoter was done after co-agroinfiltration into mock-infected *N*. *benthamiana* leaves. Scale bars represent 10 μm. Each experiment was repeated.
(TIF)

S10 Fig Co-localization of AtFis1A/B with MCS proteins in *N*. *benthamiana* leaves. Confocal microscopy images show the partial co-localization of the GFP-AtPVA12; AtVAP27-1- GFP or GFP-AtSac1 with the RFP-AtFis1A/B either in mock-treated or TBSV-infected *N*. *benthamiana* leaves. The VROs in TBSV-infected cells are marked by p33-BFP. Expression of the above proteins was from 35S promoter via co-agroinfiltration into *N*. *benthamiana* leaves. See further details in Fig 5A. Scale bars represent 10 μm. Each experiment was repeated.
(TIF)

S11 Fig BiFC assays in Fis1-silenced plants supporting TBSV replication. Interactions between p33-cYFP and either nYFP-AtVAP27-1 or nYFP-AtPAV12 VAP proteins were detected by BiFC in *N*. *benthamiana* leaves infected with TBSV. Expression of the above proteins from 35S promoter was done after co-agroinfiltration into *N*. *benthamiana* leaves with silenced Fis1 (TRV-Fis1-3’) or control (TRV-MBP). See further experimental details in Fig 11. Scale bars represent 10 μm. Each experiment was repeated.
(TIF)

S12 Fig Analysis of vsiRNA accumulation in Fis1-silenced plants supporting TBSV replication. Top panel: Northern blot analysis of the accumulation of vsiRNAs in *N*. *benthamiana* plants woth knockeddown Fis1 versus control (TRV-MBP) plants. The plants were inoculated with TBSV and samples for RNA extraction were collected 2 d later. The vsiRNAs are encircled. A 21 nt long RNA size-marker is shown on the right. Middle two panels: Accumulation level of TBSV (+)RNAs and (-)RNAs are measured by northern blotting in the samples shown above. Bottom panel: 18S ribosomal RNA level as a loading control. The experiment was repeated.
(TIF)

S1 Text Supplementary material and methods. (DOCX)

S1 Table List of plasmids constructed in this study. (DOCX)

S2 Table List of plasmids described in previous studies. (DOCX)

S3 Table List of primers used in this study. (DOCX)
