# Supplementary figures and images for: Dynamic interplay between the co-opted Fis1 mitochondrial fission protein and membrane contact site proteins in supporting tombusvirus replication
Source: PLoS Pathog. 2021 Mar 16;17(3):e1009423. doi: 10.1371/journal.ppat.1009423 (PMC7997005; doi:10.1371/journal.ppat.1009423)

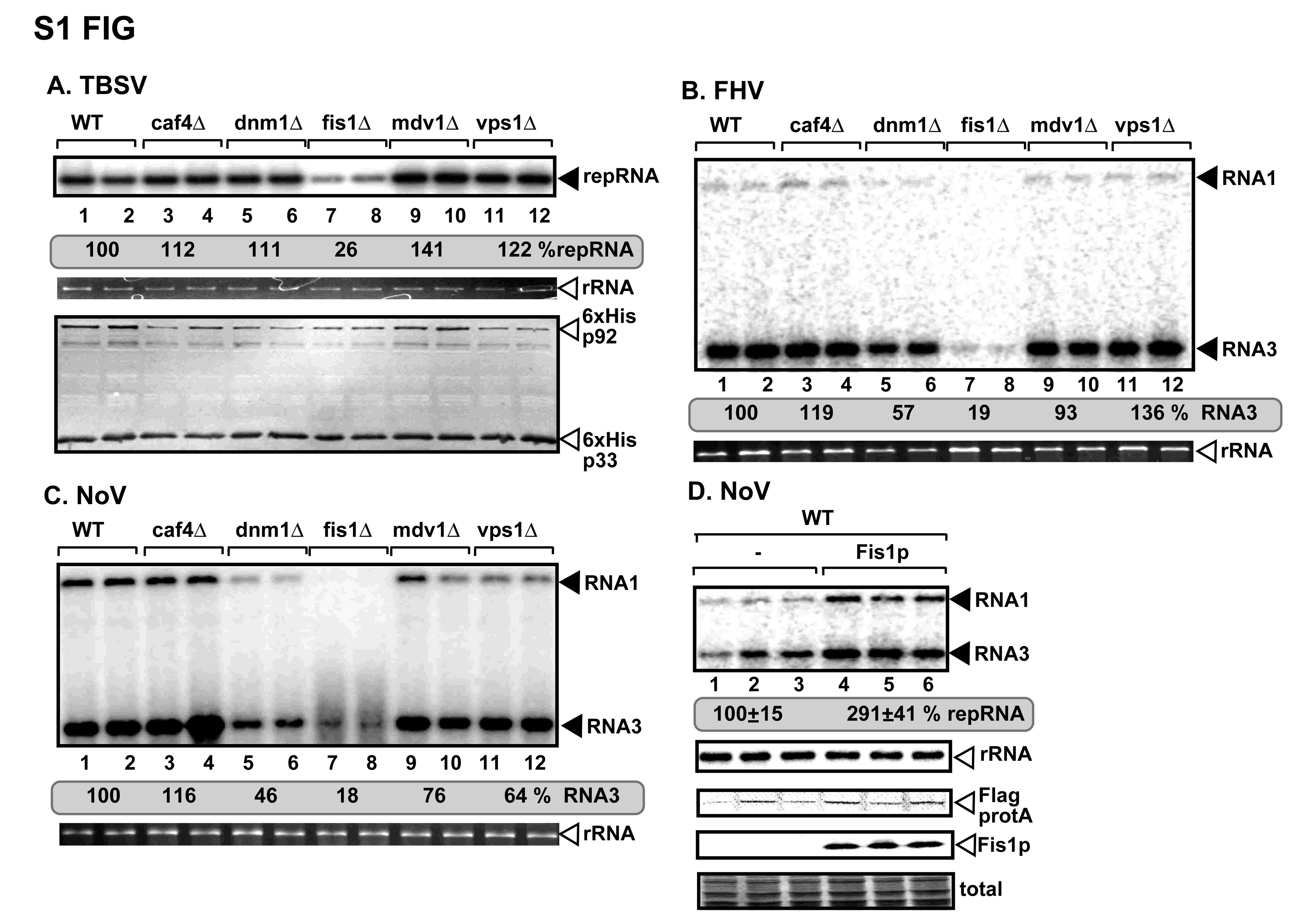

Supplement: S1 Fig — (A) Deletion of FIS1 of the mitochondrial fission complex inhibits replication of TBSV in yeast. Top panel: northern blot analyses of repRNA using a 3’ end specific probe demonstrate reduced accumulation of repRNA in fis1Δ yeast strain in comparison with deletion of the other four genes that form the mitochondrial fission complex. Viral proteins His6-p33 and His6-p92 of TBSV were expressed from the galactose-inducible GAL1 promoter, whereas the repRNA was expressed from the constitutive ADH1 promoter. Second panel: Ethidium-bromide stained agarose gels show 18S ribosomal RNA as a loading control. Bottom images: western blot analysis of the level of His6-p33 and His6-p92 proteins with anti-His antibody. (B-C) Deletion of FIS1 of the mitochondrial fission complex inhibits replication of FHV and NoV insect viruses in yeast. Northern blot analyses of RNA1 and the 3’-nested subgenomic RNA3 using a 3’ end specific probe demonstrate reduced accumulation of viral RNAs in fis1Δ yeast strain in comparison with deletion of the other four genes that form the mitochondrial fission complex. The FHV RNA1 was expressed from the GAL1 promoter, whereas protein A replication protein was expressed from the CUP1 promoter. In case of NoV, RNA1 was expressed from the CUP1 promoter, whereas the NoV protein A replication protein was expressed from the CUP1 promoter. Additional details can be found in Fig 1. (D) Over-expression of the yeast Fis1p from a plasmid enhances NoV RNA replication in yeast. Additional details can be found in Fig 1. (TIF) [file ppat.1009423.s001.tif]

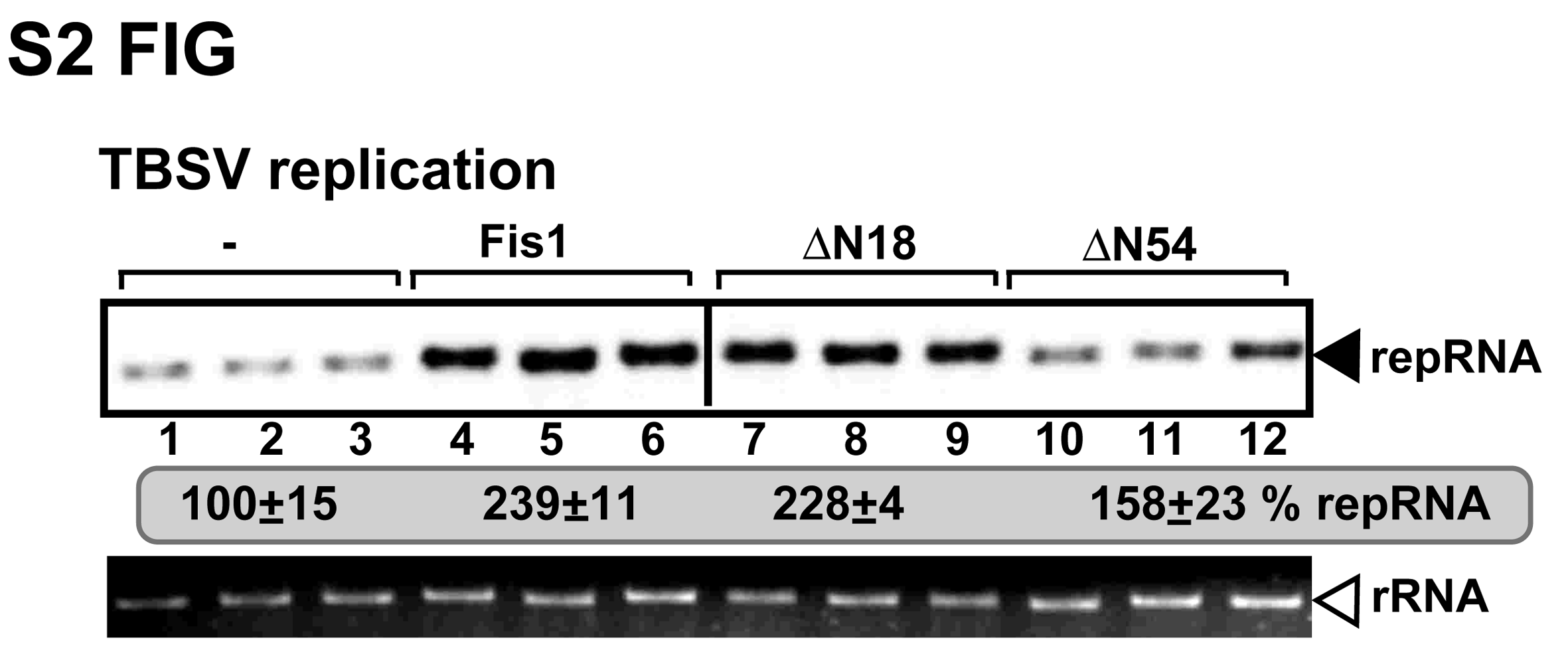

Supplement: S2 Fig — Northern blot analyses of repRNA using a 3’ end specific probe demonstrates reduced accumulation of repRNA in fis1Δ yeast strain expressing the shown N-terminal deletion mutants in comparison with the WT Fis1p. Viral proteins His6-p33 and His6-p92 of TBSV were expressed from plasmids from the GAL1 promoter, while DI-72(+) repRNA was expressed from the GAL10 promoter. His6-Fis1p was expressed from the GAL1 promoter from a plasmid. Second panel: Ethidium-bromide stained agarose gel shows 18S ribosomal RNA as a loading control. (TIF) [file ppat.1009423.s002.tif]

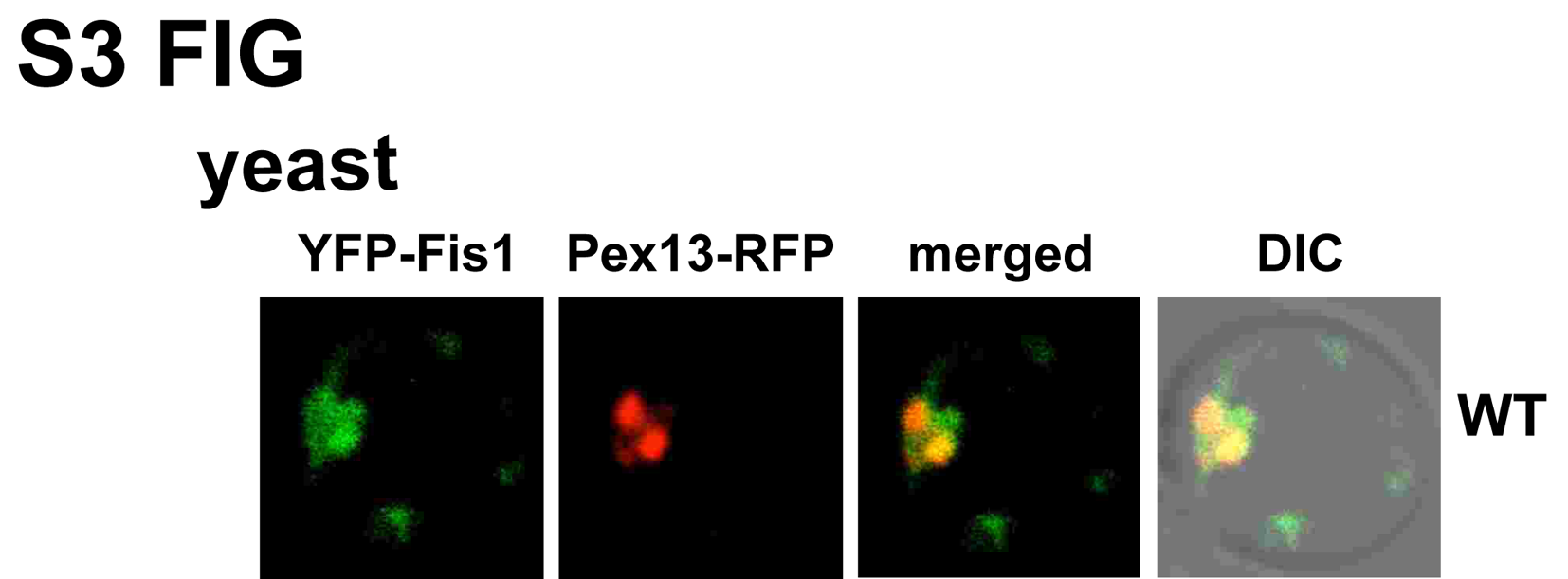

Supplement: S3 Fig — DIC images are shown on the right. See further details in Fig 3G. (TIF) [file ppat.1009423.s003.tif]

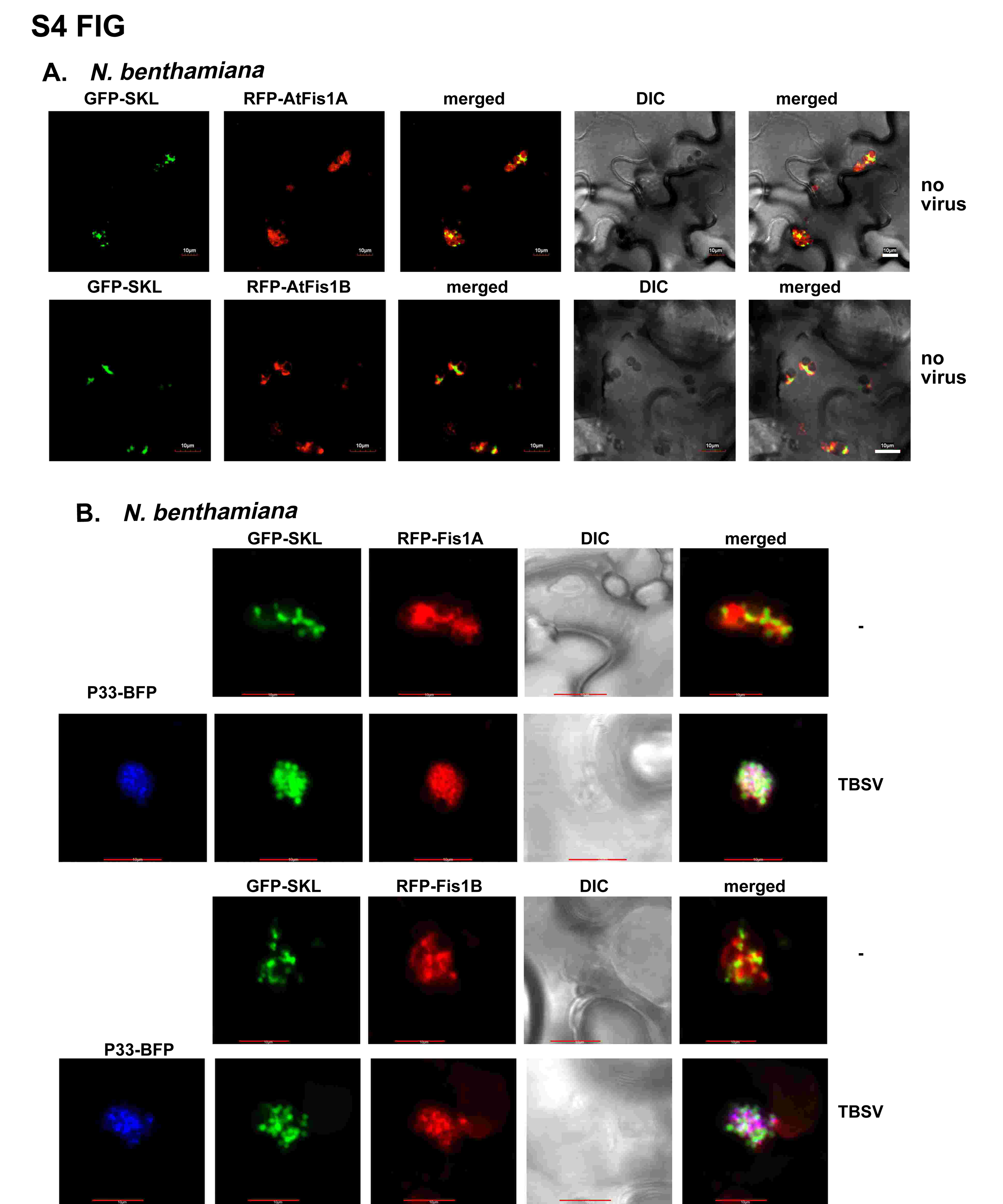

Supplement: S4 Fig — (A-B) See further details in Fig 5A. (TIF) [file ppat.1009423.s004.tif]

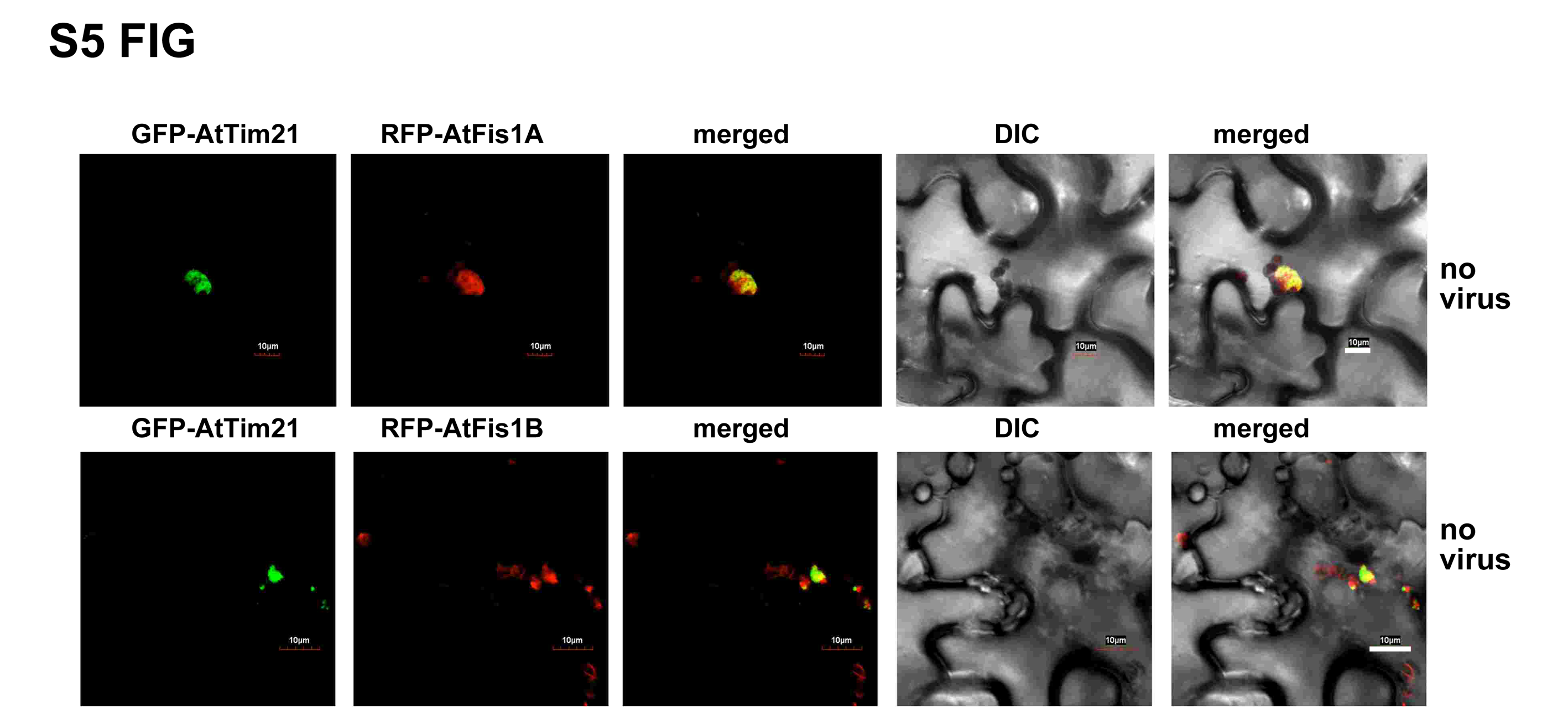

Supplement: S5 Fig — See further details in Fig 6A. (TIF) [file ppat.1009423.s005.tif]

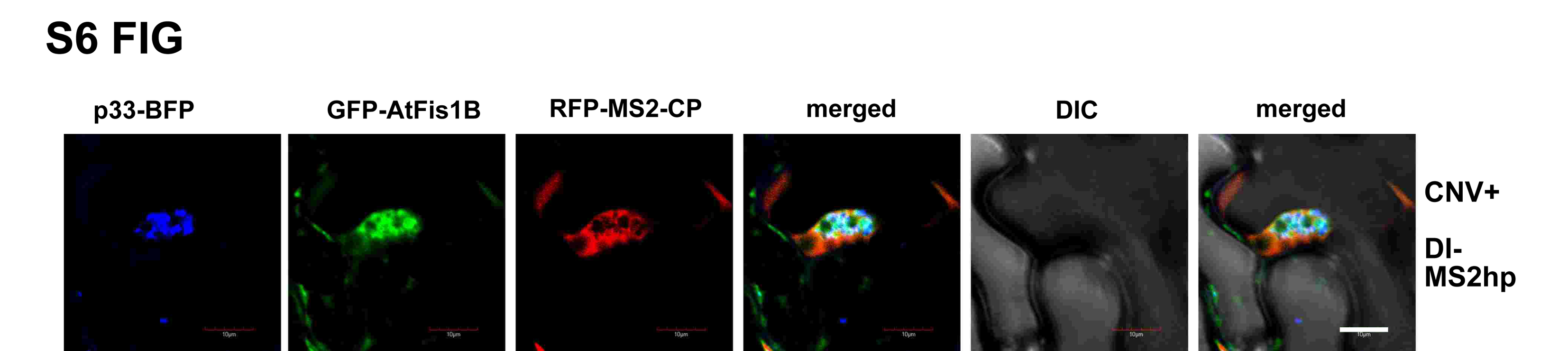

Supplement: S6 Fig — The (+)repRNA carries 6 copies of the 19 nt long hairpin sequence from the MS2 phage, which is specifically recognized by the RFP-tagged MS2-CP (coat protein). Confocal microscopy images show the co-localization of the (+)repRNA with GFP-AtFis1B within the replication compartment, which is marked by p33-BFP. Expression of the above proteins and the repRNA was from 35S promoter via co-agroinfiltration into N. benthamiana leaves also infected with CNV. Scale bars represent 10 μm. Each experiment was repeated. (TIF) [file ppat.1009423.s006.tif]

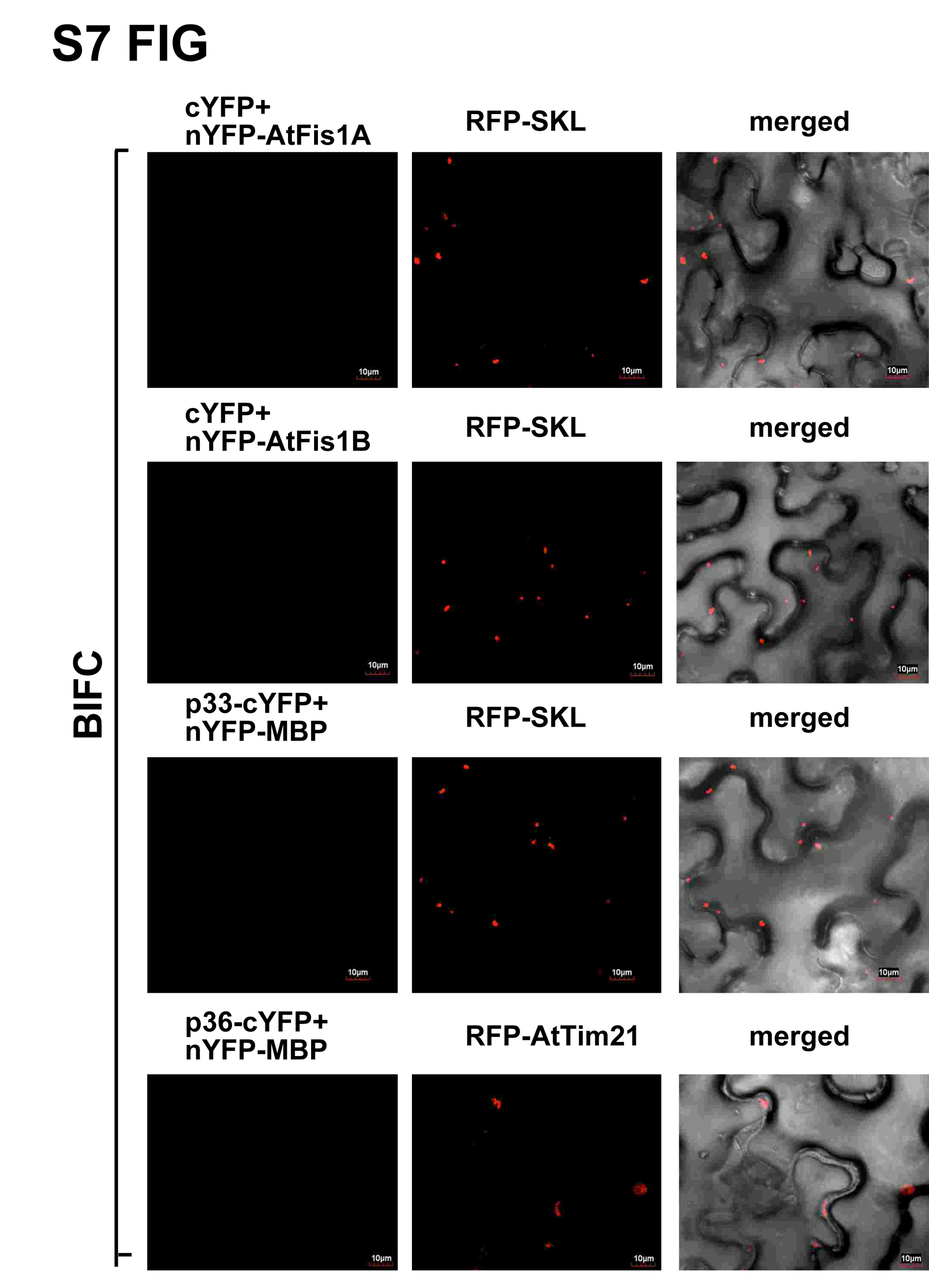

Supplement: S7 Fig — The lack of BiFC signals in these experiments indicates that the interactions between TBSV p33-cYFP and the CIRV p36-cYFP replication proteins and the nYFP-AtFis1A and nYFP-AtFis1B proteins are specific. See further details in Figs 5C and 6C. (TIF) [file ppat.1009423.s007.tif]

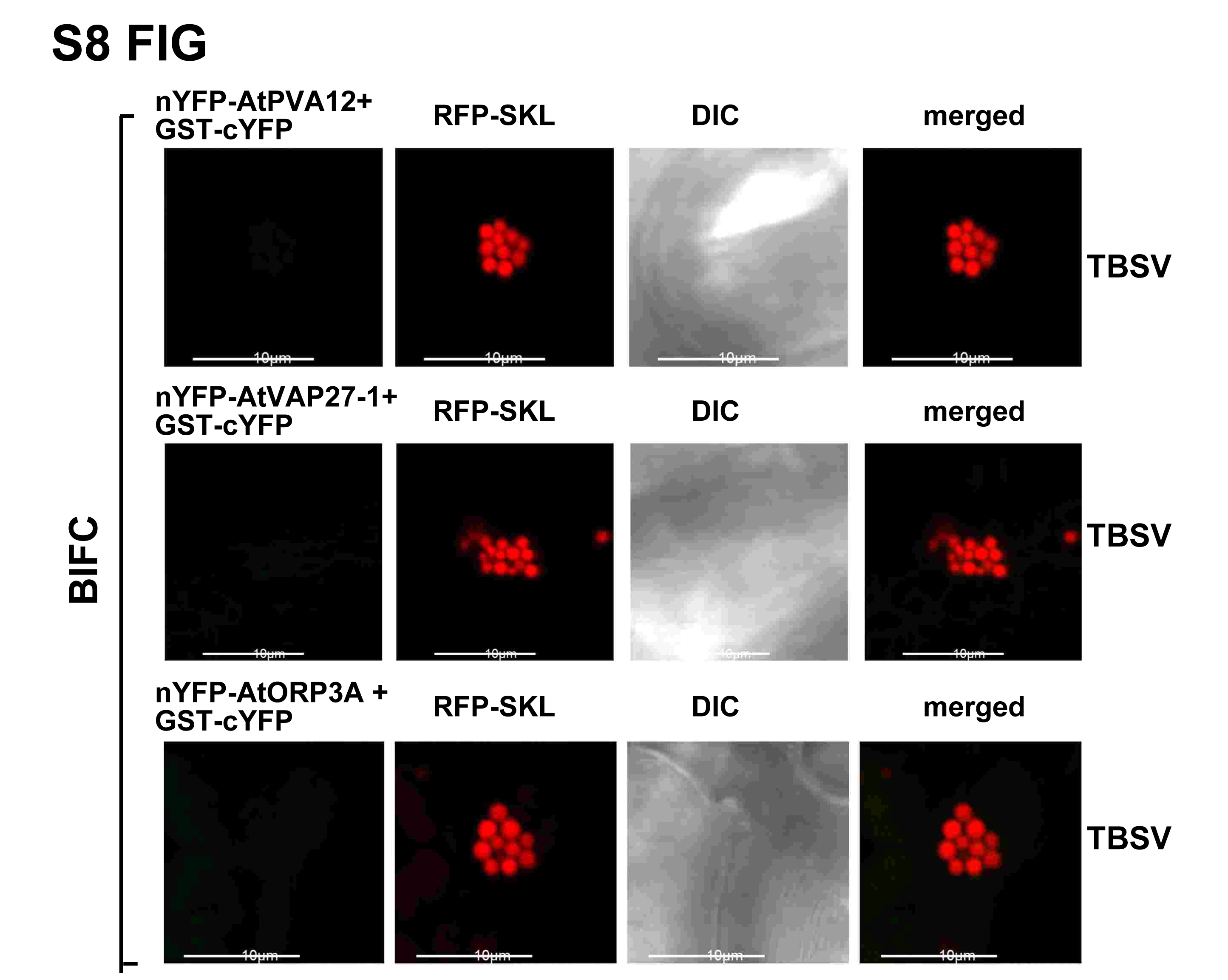

Supplement: S8 Fig — The lack of BiFC signals in these experiments indicates that the interactions between nYFP-AtVAP27-1 and the nYFP-AtPVA12 VAP proteins and the cYFP-AtFis1A and cYFP-AtFis1B proteins are specific. See further details in Fig 8D. Similarly, the lack of BiFC signals in these experiments indicates that the interactions between the OSBP-like nYFP-AtORP3A protein and the cYFP-AtFis1A and cYFP-AtFis1B proteins are specific. See further details in Fig 10C. (TIF) [file ppat.1009423.s008.tif]

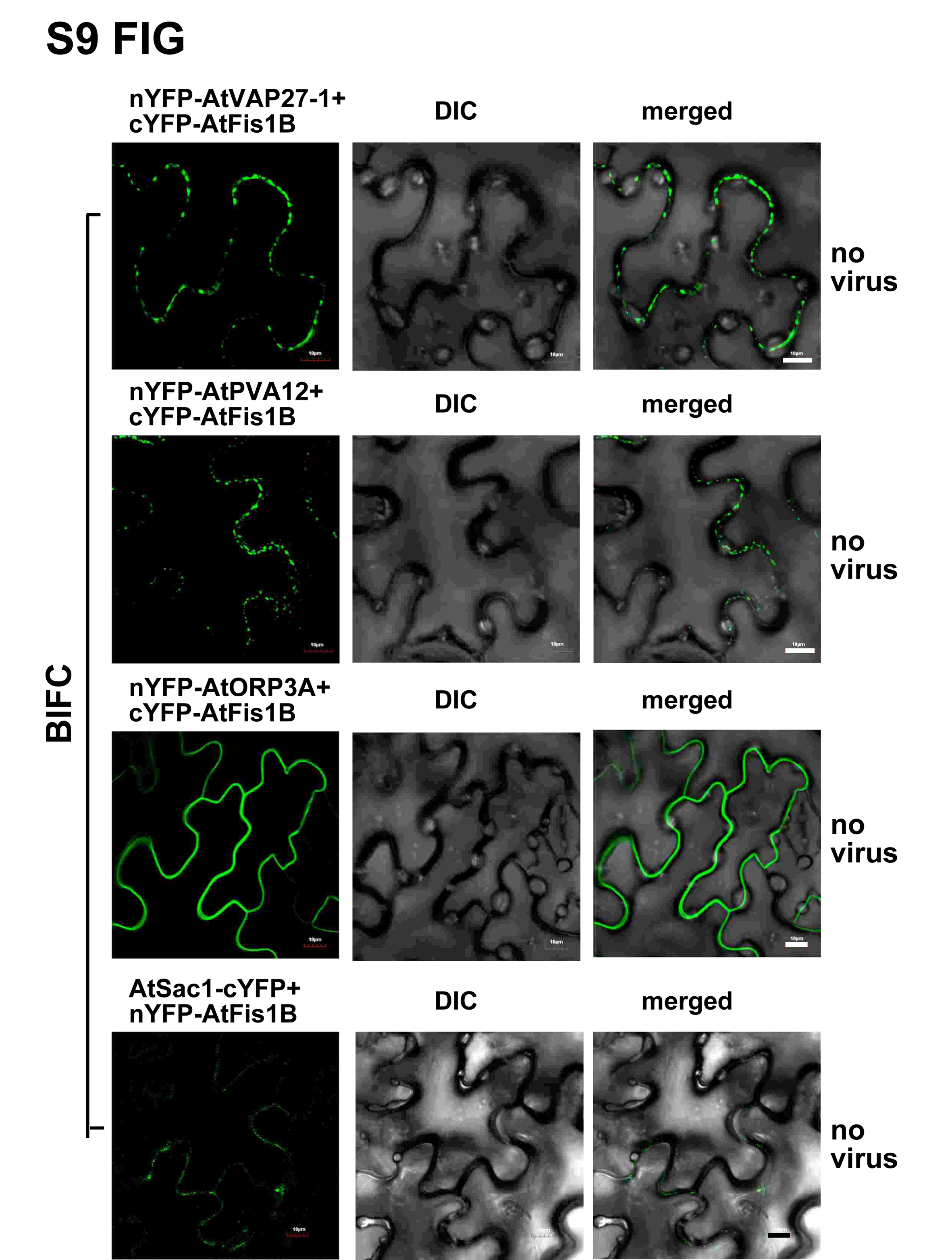

Supplement: S9 Fig — Interactions between cYFP-AtFis1B and nYFP-AtVAP27-1 or nYFP-AtPAV12 or nYFP-AtORP3A proteins were detected by BiFC. Also, interaction between nYFP-AtFis1B and AtSac1-cYFP was detected by BiFC in N. benthamiana leaves. Expression of the above proteins from 35S promoter was done after co-agroinfiltration into mock-infected N. benthamiana leaves. Scale bars represent 10 μm. Each experiment was repeated. (TIF) [file ppat.1009423.s009.tif]

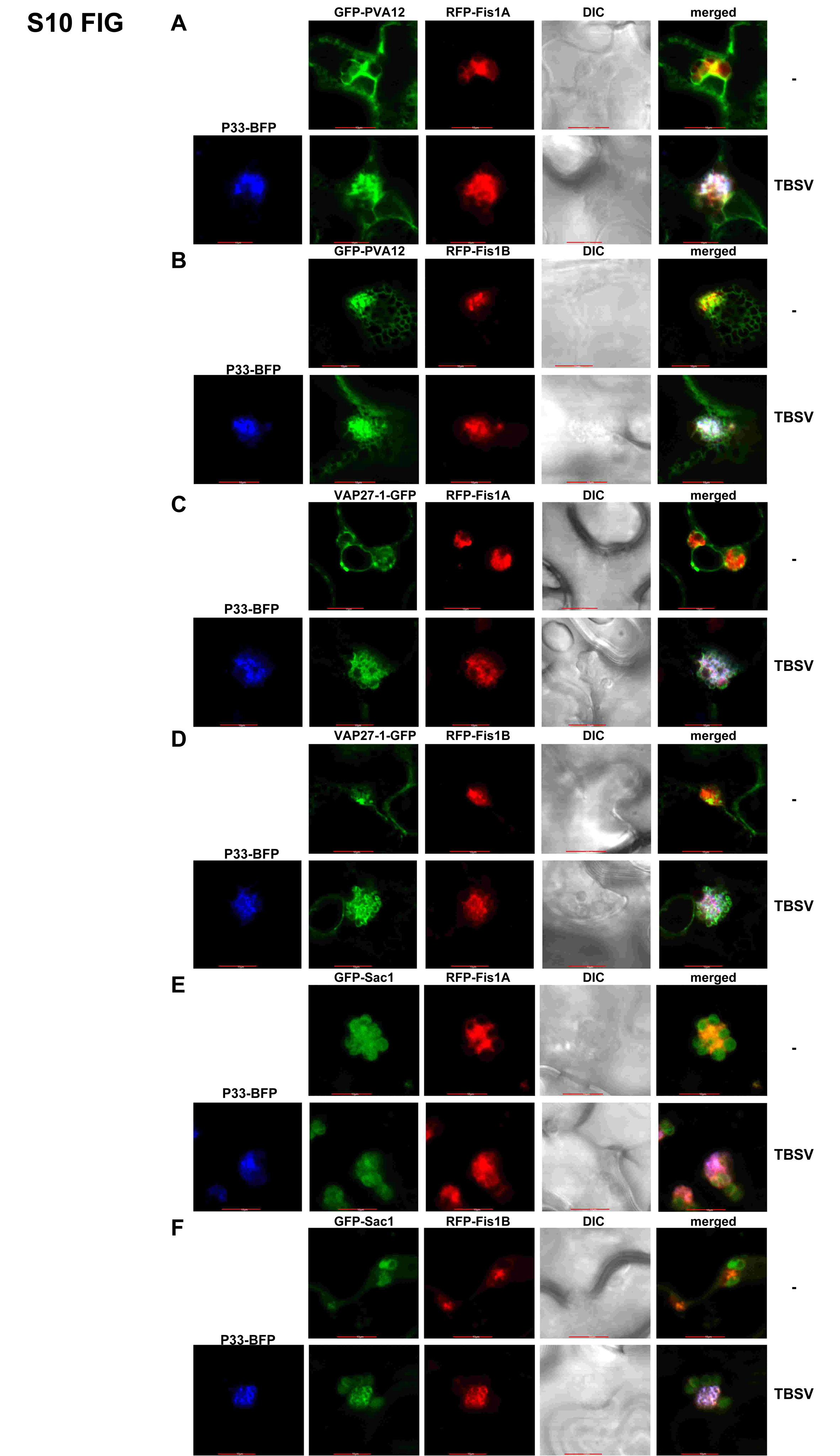

Supplement: S10 Fig — Confocal microscopy images show the partial co-localization of the GFP-AtPVA12; AtVAP27-1- GFP or GFP-AtSac1 with the RFP-AtFis1A/B either in mock-treated or TBSV-infected N. benthamiana leaves. The VROs in TBSV-infected cells are marked by p33-BFP. Expression of the above proteins was from 35S promoter via co-agroinfiltration into N. benthamiana leaves. See further details in Fig 5A. Scale bars represent 10 μm. Each experiment was repeated. (TIF) [file ppat.1009423.s010.tif]

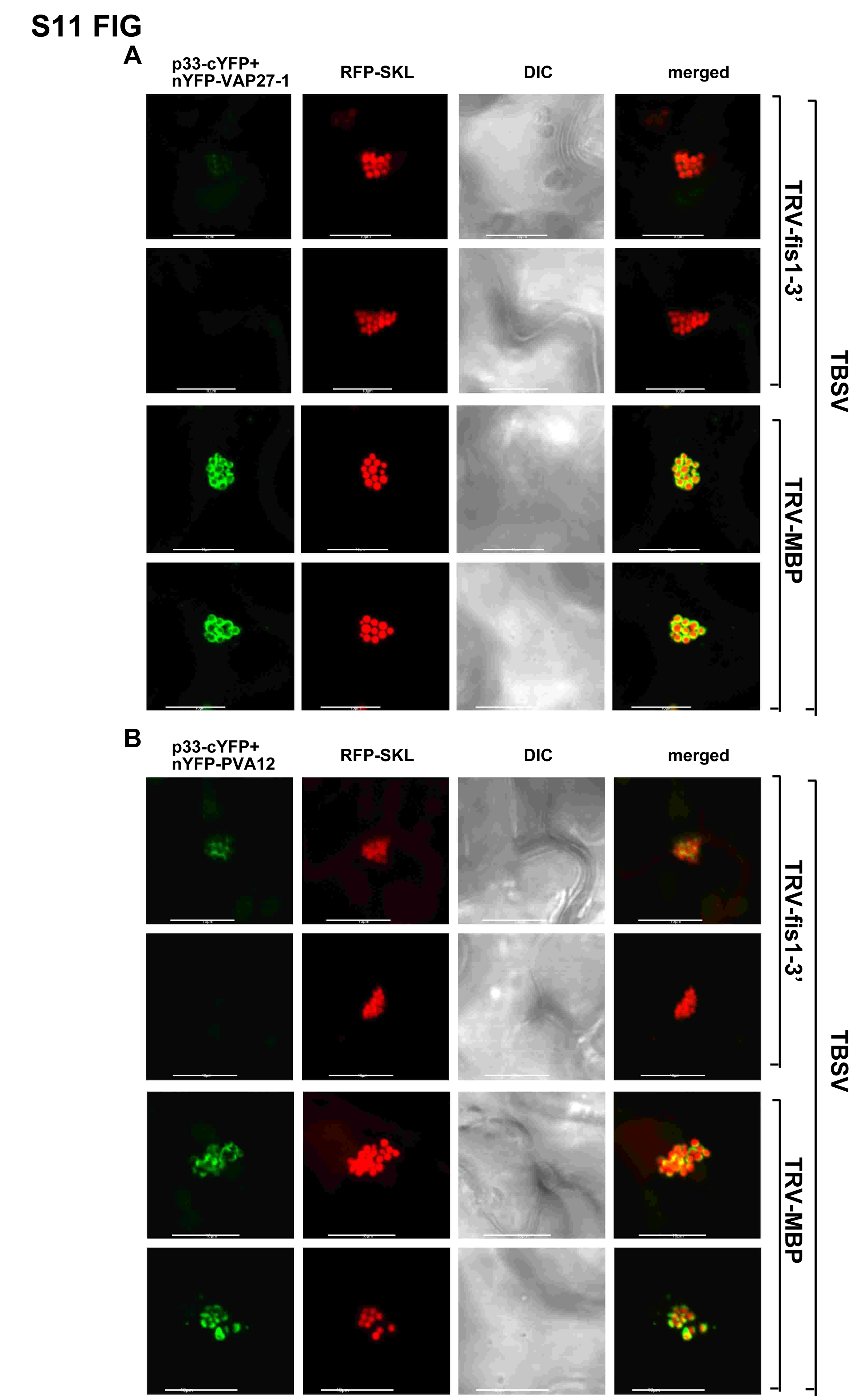

Supplement: S11 Fig — Interactions between p33-cYFP and either nYFP-AtVAP27-1 or nYFP-AtPAV12 VAP proteins were detected by BiFC in N. benthamiana leaves infected with TBSV. Expression of the above proteins from 35S promoter was done after co-agroinfiltration into N. benthamiana leaves with silenced Fis1 (TRV-Fis1-3’) or control (TRV-MBP). See further experimental details in Fig 11. Scale bars represent 10 μm. Each experiment was repeated. (TIF) [file ppat.1009423.s011.tif]

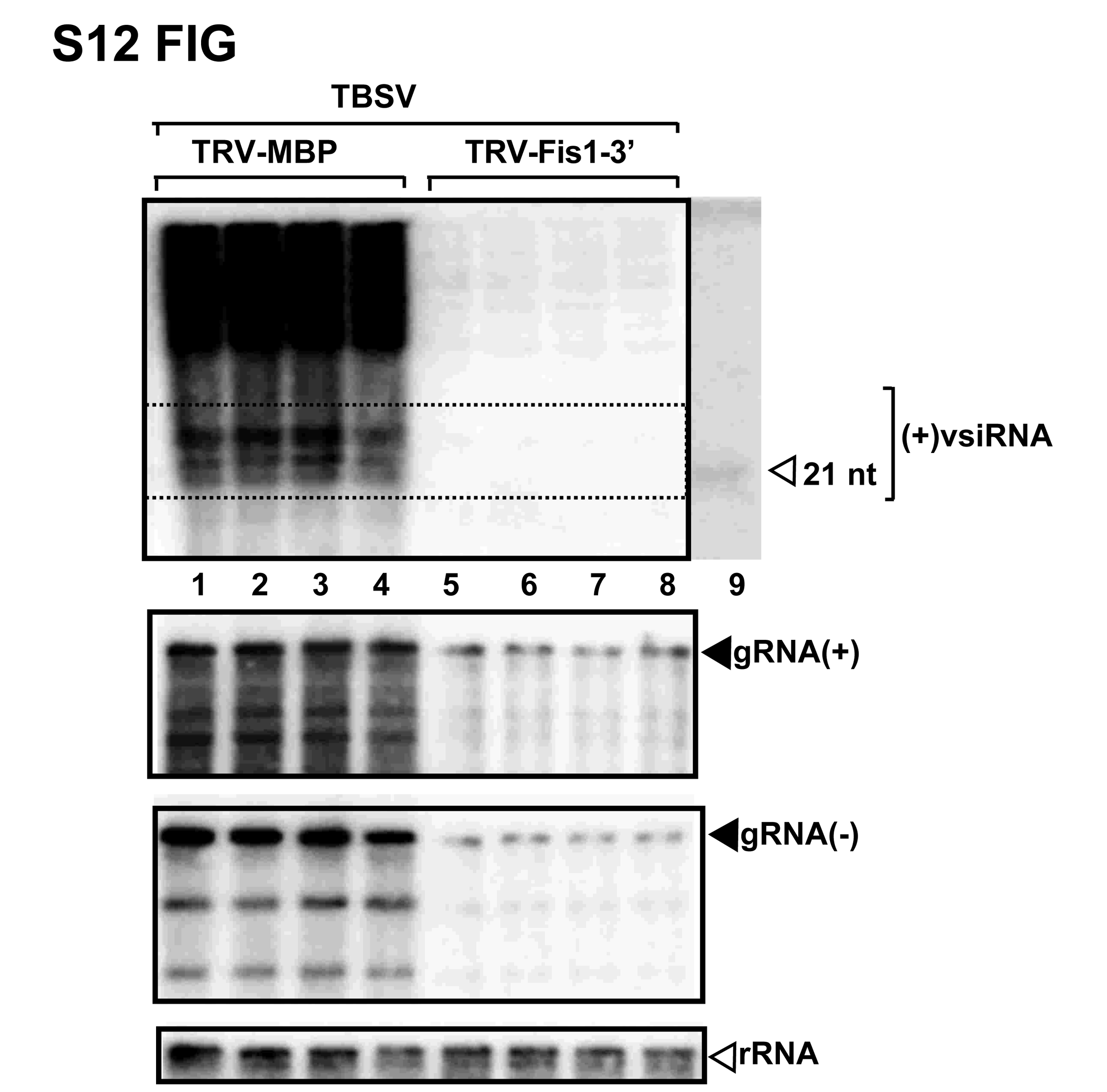

Supplement: S12 Fig — Top panel: Northern blot analysis of the accumulation of vsiRNAs in N. benthamiana plants woth knockeddown Fis1 versus control (TRV-MBP) plants. The plants were inoculated with TBSV and samples for RNA extraction were collected 2 d later. The vsiRNAs are encircled. A 21 nt long RNA size-marker is shown on the right. Middle two panels: Accumulation level of TBSV (+)RNAs and (-)RNAs are measured by northern blotting in the samples shown above. Bottom panel: 18S ribosomal RNA level as a loading control. The experiment was repeated. (TIF) [file ppat.1009423.s012.tif]
