# Supplementary material for: Dynamic interplay between the co-opted Fis1 mitochondrial fission protein and membrane contact site proteins in supporting tombusvirus replication
Source: PLoS Pathog. 2021 Mar 16;17(3):e1009423. doi: 10.1371/journal.ppat.1009423 (PMC7997005; doi:10.1371/journal.ppat.1009423)
Supplement: S1 Text — (DOCX) [file ppat.1009423.s013.docx]

**S1 Text. Supplementary material and methods**

**Yeast membrane two-hybrid assay**

The yeast membrane two-hybrid assay (MYTH) based on the split-ubiquitin system was performed following the previous publication [1]. Briefly, to determine the interaction between Fis1p and the TBSV p33, plasmids pGAD-BT2-N-His33 was transformed with one of the following plasmids: pPR-N-RE, pPR-N-RE-ssa1, pPR-N-RE-AtFis1A, pPR-N-RE-AtFis1B, pPR-N-RE-ScFis1, pPR-N-RE-ScFis1∆N18, pPR-N-RE-ScFis1∆N54, pPR-N-RE-ScFis1∆N92, pPR-N-RE-ScFis1∆C25 or pPR-N-RE-ScFis1∆C63. To determine the interaction between Fis1p and other membrane contact sites-associated proteins, pBT3-N-ScFis1 was transformed with one of the following plasmids: pPR-N-RE, pPR-N-RE-ScFis1p, pPRN-P33, pPRN-Scs2p, pPRN-AtVAP27-1, pPRN-AtVAP27-2, pPRN-Osh6p or pPR-N-RE-ScSac1. Transformed yeast colonies were suspended in 100 μl water, 8 μl of which cultured on TL^-^ plates as loading control or on TLHA^-^ plates to score protein interactions [2].

**Co-purification assay with proteins expressed in yeast**

For co-purification of Fis1 proteins with the TBSV p33/p92 replication proteins from yeasts, plasmids HpGBK-CUP1-Hisp33/Gal-DI-72 and LpGAD-CUP1-His92 (as a control) or HpGBK-CUP1-Flagp33/Gal-DI-72 and LpGAD-CUP1-Flag92 were co-transformed with UpYES-HisScFis1 or UpYES-HisAtFis1A or UpYES-HisAtFis1B into yeast strain BY4741. For co-purification of Fis1 proteins with the CIRV p36/p95 replication proteins from yeasts, plasmids HpGBK-CUP1-Hisp36/Gal-DI-72 and LpGAD-CUP1-His95 (as a control) or HpGBK-CUP1-Flagp36/Gal-DI-72 and LpGAD-CUP1-Flag95 were co-transformed with UpYES-HisScFis1 or UpYES-HisAtFis1A or UpYES-HisAtFis1B into yeast strain BY4741.

For co-purification of different Fis1p deletion mutants with the TBSV p33/p92 replication proteins from yeasts, yeast strain BY4741 was co-transformed with plasmids HpGBK-CUP1-Flagp33/Gal-DI-72 and LpGAD-CUP1-Flag92 and one of the following plasmids: UpYES-HisScFis1, UpYES-HisScFis1∆N18, UpYES-HisScFis1∆N54, UpYES-HisScFis1∆N92, UpYES-HisScFis1∆C25 or UpYES-HisScFis1∆C63.

For co-purification of Sac1p, Scs2p or Osh6p with the TBSV p33/p92 replication proteins from yeasts, plasmids HpGBK-CUP1-Hisp33/Gal-DI-72 and LpGAD-CUP1-His92 (as a control) or HpGBK-CUP1-Flagp33/Gal-DI-72 and LpGAD-CUP1-Flag92 were co-transformed with UpYC-ScSac1 or UpYC-ScScs2 or UpYC-ScOsh6 into yeast strain BY4741 or Fis1∆. Meanwhile, yeast strain BY4741-ADH-Hisp92 was co-transformed with HpGBK-CUP1-Flagp33/Gal-DI-72 and LpGAD-HisScFis1 or LpESC-empty and one of the following plasmids: UpYC-ScSac1 or UpYC-ScScs2 or UpYC-ScOsh6, whereas HpGBK-CUP1-Hisp33/Gal-DI-72 was used as a control.

For co-purification of Fis1p with the TBSV p33/p92 replication proteins from yeasts overexpressing Dnm1p, yeast strain BY4741-ADH-Hisp92 was co-transformed with HpGBK-CUP1-Flagp33/Gal-DI-72, LpGAD-HisScFis1 and UpBG1805-Dnm1-ZZ or UpYES-NT (as a negative control).

All transformed yeast cells were pre-grown in SC-ULH^−^ media supplemented with 2% glucose and 100 μM BCS at 29°C for 16 h. Then yeast cultures were resuspended in SC-ULH^−^ medium supplemented with 2% galactose and 100 μM BCS and grown at 23°C for 24 h, followed by culturing yeast cells in SC-ULH^−^ medium supplemented with 2% galactose and 50 μM CuSO_4_ at 23°C for 6 h. The cultures were resuspended and incubated in 35 ml phosphate-buffered saline (PBS) buffer containing 1% formaldehyde for 1 h on ice to cross-link proteins. Then, glycine (to 0.1M) was added to quench the formaldehyde and the yeasts were incubated on ice for 5 min. Finally, yeast pellets were harvested after washing twice with PBS buffer and proteins were Flag affinity purified as described previously [3].

To understand the time dynamics of Fis1p association with the viral replicase, transformed yeast cells were pre-grown in SC-ULH^−^ medium supplemented with 2% glucose and 100 μM BCS at 29°C for 16 h. Then yeast cultures were resuspended in SC-ULH^−^ medium supplemented with 2% galactose and 100 μM BCS and grown at 23°C for 24 h, followed by culturing yeast cells in SC-ULH^−^ medium supplemented with 2% galactose and 50 μM CuSO_4_ at 23°C for 6 h. Next, the yeast cells were shifted to SC-ULH^−^ medium supplemented with 2% glucose and cycloheximide (100 µg/ml) and samples were taken at 0, 1 h and 2.5 h time points. Yeast cultures were treated with formaldehyde and glycine and performed Flag-affinity purification as described above.

**Co-purification assay with proteins expressed in plants**

Co-purification assay from plants was performed with slight modifications of a previously described method [4]. Various combination of expression vectors were co-infiltrated into *N. benthamiana* leaves and samples were harvested at 2.5 days post agroinfiltration and ground in a cooled mortar in PPEB buffer (10% [v/v] glycerol, 25 mM Tris-HCl, pH 7.5, 1 mM EDTA, 150 mM NaCl, 10 mM DTT, 0.5% [v/v] Triton X-100 and protease inhibitor cocktail). The supernatant was incubated with anti-Flag M2 affinity agarose (Sigma-Aldrich) in Bio-spin chromatography columns (Bio-rad) for 2 h at 4°C on a rotator, followed by washing with CP buffer (10% [v/v] glycerol, 25 mM Tris-HCl, pH 7.5, 1 mM EDTA, 150 mM NaCl, 1mM DTT and 0.1% [v/v] Triton X-100). Elution of purified proteins was as described above [3].

**Pull-down assay**

The pull-down assay has been described in [5]. Briefly, pGEX-His-RE-ScFis1-∆TM, pGEX-His-RE-AtFis1A-∆TM and pGEX-His-RE-AtFis1B-∆TM were transformed into Epicurion Bl21-codon-plus (DE3)-R1L cells (Stratagene) and the expression of those proteins was induced by IPTG as described [5]. The *E. coli* cells were broken by sonication and the supernatants were used for purification with a GST resin [5]. For expression of MBP or MBP-tagged T33C (C-terminal portion of the TBSV p33) and C36C (C-terminal portion of the CIRV p36), pMALc-2X, pMALc-2X-T33C and pMALc-2X-C36C were also transformed into Epicurion Bl21-codon-plus (DE3)-R1L cells, followed by IPTG induction. After sonication, 100 μl lysates were incubated with 15 μl amylose resin (NEB) in Bio-spin chromatography columns for 1 h at 4°C, followed by four times washing with column buffer [5]. The amylose columns containing the MBP or MBP-tagged T33C or C36C were then incubated with 4 μg of the purified GST-His_6_-ScFis1-∆TM, GST-His_6_-AtFis1A-∆TM, GST-His_6_-AtFis1B-∆TM for 2 h at 4°C. Then, the washed beads were incubated in 1× SDS loading buffer for 10 min at 85°C. The MBP-tagged proteins were analyzed by SDS-PAGE electrophoresis followed by coomassie staining and GST- His_6_-tagged proteins were separated by SDS-PAGE for protein gel blot analysis with anti-His antibody [5].

**Confocal microscopic analysis of yeast cells**

To analyze the subcellular localization of Fis1p in the presence or absence of TBSV p33 in yeast, UpYC-YFP-ScFis1, LpRS315-pex13-RFP and HpEsc-BFP-T33/GAL-DI-72 or HpESC-empty were co-transformed into BY4741 and fis1∆ yeast strains. To analyze the subcellular localization of Fis1p in the presence of virus replication in yeast, BY4741 and Fis1∆ yeast strains were co-transformed with LpGAD-CUP1-His92, HpEsc-BFP-T33/GAL-DI-72 and UpYC-YFP-ScFis1. Transformed yeast cells were grown in 2 ml SC-ULH^−^ medium supplemented with 2% galactose and 50 μM CuSO_4_ for 18 h at 23°C. The confocal images were captured sequentially with an Olympus FV1000 microscope (Olympus America) [6].

**Confocal microscopic analysis of plant epidermal cells**

To analyze the subcellular localization of host proteins in the presence or absence of viral components in *N. benthamiana* leaves, plasmids pGD-35S-BFP-AtFis1A, pGD-35S-BFP-AtFis1B, pGD-35S-T33-RFP, pGD-35S-C36-RFP, pGD-35S-GFP-SKL (as a peroxisome marker) and pGD-35S-GFP-AtTim21 (as a mitochondrial marker) were transformed into agrobacterium strain C58C1. Then agrobacterium suspensions in various combinations were co-infiltrated into *N. benthamiana* leaves, followed by virus inoculation with sap at 16 h post agroinfiltration (dpai). At 2.5 dpai, the agroinfiltrated leaves were subjected to confocal laser microscopy.

To detect interaction of AtFis1A/B with TBSV p33 or CIRV p36 replication proteins using bimolecular fluorescence complementation assay (BiFC), plasmids pGD-35S-T33-cYFP, pGD-35S-C36-cYFP, pGD-35S-C-cYFP (as a negative control), pGD-35S-nYFP-AtFis1A, pGD-35S-nYFP-AtFis1B, pGD-35S-nYFP-MBP (as a negative control), pGD-35S-RFP-SKL (as a peroxisome marker) and pGD-35S-RFP-AtTim21 (as a mitochondrial marker) were transformed separately into agrobacterium strain C58C1. The agrobacterium transformants in various combinations were used to co-infiltrate *N. benthamiana* leaves, which were harvested and then subjected to confocal microscopic analysis at 2 dpai.

To test whether AtFis1A or AtFis1B can interact with AtSac1, AtVAP27-1, AtPVA12 and AtORP3A in the presence or absence of virus infection, plasmids pGD-35S-cYFP-AtFis1A, pGD-35S-cYFP-AtFis1B, pGD-35S-nYFP-AtVAP27-1, pGD-35S-nYFP-AtPVA12, pGD-35S-nYFP-AtORP3A, pGD-35S-nYFP-AtFis1A, pGD-35S-nYFP-AtFis1B and pGD-35S-AtSac1-cYFP were transformed into agrobacterium strain C58C1. The agrobacterium transformants in various combinations were used to co-infiltrate *N. benthamiana* leaves, followed by virus inoculation with sap at 16 h post agroinfiltration. At 2.5 dpai, the agroinfiltrated leaves were subjected to confocal laser microscopy.

To provide direct evidence that AtFis1A or AtFis1B was localized within the tombusvirus replication compartment, a dsRNA sensor assay, described in [7], was applied to detect the localization of viral double strand RNA (dsRNA) generated during virus replication. The dsRNA sensor YN-B2 and YC-VP35 plasmids [7] were agro-infiltrated into *N. benthamiana* leaves at OD600 of 0.1, respectively, together with either pGD-35S-BFP-AtFis1A or pGD-35S-BFP-AtFis1B and either pGD-35S-T33-RFP or pGD-35S-C36-RFP, followed by virus inoculation with sap at 16 h post agroinfiltration. At 2.5 dpai, the agroinfiltrated leaves were subjected to confocal laser microscopy.

To examine the co-localization between Fis1 and MCS proteins (Sac1, PVA12, and VAP27-1) in combination with the TBSV p33 replication protein in plant, plasmids pGD-p33-BFP, pGD-RFP-AtFis1A, pGD-RFP-AtFis1B, pGD-GFP-AtSac1, pGD-GFP-AtPVA12 or pGD-AtVAP27-1-GFP were transformed into C58C1 competent cells. Agrobacteria carrying pGD-p33-BFP (0.3 OD_600_), pGD-p33-BFP (0.3 OD_600_) or pGD-RFP-AtFis1B (0.3 OD_600_), pGD-GFP-AtSac1 (0.3 OD_600_) or pGD-GFP-AtSac1 (0.3 OD_600_) or pGD-AtVAP27-1-GFP (0.3 OD_600_) and pGD-p19 (0.3 OD_600_) were co-infiltrated into *N. benthamiana* leaves. TBSV sap was inoculated on the leaves 16 h post agroinfiltration. Infiltrated leaves were subjected to confocal laser microscopy 48 h post sap inoculation.

To test if Fis1 was recruited into viral replication compartments, agrobacteria pGD-p33-BFP (0.3 OD_600_), pGD-RFP-AtFis1A (0.3 OD_600_) or pGD-RFP-AtFis1B (0.3 OD_600_), pGD-GFP-SKL (0.2 OD_600_) and pGD-p19 (0.1 OD_600_) were co-infiltrated into *N. benthamiana* leaves. TBSV sap was inoculated on the leaves 16 h post agroinfiltration. The co-localization of the expressed proteins was visualized by confocal microscopy as above.

To investigate the role of NbFis1 in the recruitment of MCS proteins (VAP27-1, OPR3 and PAV12) into VROs in plants, BIFC assays were conducted in *N. benthamiana* leaves. p33-cYFP (0.3 OD_600_), RFP-SKL (0.2 OD_600_) and nYFP-AtPVA12 (0.3 OD_600_), nYFP-AtVAP27-1 (0.3 OD_600_) or nYFP-AtPVA12 (0.3 OD_600_) were expressed in NbFis1-silenced plants 12 d post agroinfiltration. TBSV sap was inoculated on the leaves 16 h post agroinfiltration. Infiltrated leaves were subjected to confocal laser microscopy 48 h post sap inoculation.

**VIGS of NbFis1 in *N. benthamiana* plants**

The virus-induced gene silencing (VIGS) in *N. benthamiana* was performed as described previously [8,9]. To generate VIGS constructs TRV2-NbFis1-S1 and TRV2-NbFis1-S2, S1 fragment representing the 5’ sequence and S2 representing the 3’ sequence of NbFis1, respectively, were PCR-amplified from *N. benthamiana* cDNA and inserted into the plasmid pTRV2. 12 days after the VIGS treatment of *N. benthamiana* (agroinfiltartion of pTRV1 together with pTRV2-NbFis1-S1 or pTRV2-NbFis1-S2 or pTRV2-cGFP), the accumulation level of *N. benthamiana* NbFis1 mRNA was determined by RT-PCR with primers oligo-d(T) (for RT), #5812 and #5815 for PCR to detect NbFis1 and #2859 and #2860 for PCR to detect tubulin mRNA as an internal reference control. Then, the silenced leaves were either sap inoculated with TBSV or TCV inocula or agroinfiltrated with pGD-CNV-20Kstop or pGD-CIRV, to launch viral replication. At different time points, samples from the inoculated and systemic leaves were collected, followed by total RNA extraction and Northern blot analysis as described previously [9].

**Plant protoplasts preparation and RNA transfection**

Protoplasts preparations from plant leaves were obtained by using a previously described method with some modifications [10]. Briefly, NbFis1-silenced and mock-treated *N. benthamiana* leaves were harvested at 12 days post TRV-induced VIGS. Then these leaves were sliced into 0.5-1 mm strips, digested with an enzyme solution containing 1.2% [w/v] Cellulase, 0.16% [w/v] Macerozyme, 0.12% [w/v] BSA and 0.5 M mannitol. To improve the isolation of protoplasts, leaf strips were vacuum infiltrated for 20 min in the dark using Vacufuge Plus (eppendorf) and further digested in the dark for at least 3 h at room temperature. The obtained protoplasts were passed through a sieve set (Scienceware Mini-Sieve Microsieve Set from Fisher cat# 14-306A) and collected by centrifugation at 900 rpm for 2 min, followed by washing once with the W5 solution (154 mM NaCl, 125 mM CaCl_2_, 5 mM KCl, 2 mM MES pH5.7) and re-suspending in the W5 solution. Then, 0.6 M sucrose was layered under the W5 solution with protoplasts and centrifuged at 900 rpm for 3 min. Protoplasts were transferred from the interface between the W5 solution and 0.6 M sucrose layers into same amount of W5 solution, followed by washing once with the W5 solution and re-suspending at 2 x10^5^ ml^–1^ in MMG solution (4 mM MES pH5.7, 0.4 M mannitol and 15 mM MgCl_2_). For RNA transfection, PEG-calcium transfection solution containing 40% PEG 4000, 0.2 M mannitol and 100 mM CaCl_2_ were incubated with protoplasts and either viral RNA transcript or total RNA extracted from virus-infected plants at room temperature for up to 15 min. The transfection mixture then were diluted with the W5 solution and centrifuged at 100 g for 2 min at room temperature and incubated in WI solution (4 mM MES, pH 5.7, 0.5 M mannitol and 20 mM KCl). The protoplasts were harvested at 16 h or 24 h post transfection and subjected to RNA extraction and Northern blot analysis as described above.

**Filipin-staining of ergoterols in yeast**

BY4741 and fis1∆ yeast strains were co-transformed with plasmids HpGBK-CUP1- Hisp33/Gal-DI-72, LpGAD-CUP1-His92 and UpYES-NT empty vector or UpYES-NT-HisScFis1. The transformed yeast strains were grown at 23°C in SC-ULH^−^ medium supplemented with 2% galactose and 50 μM CuSO_4_ for 24 hours, with untreated BY4741 and fis1∆ yeast strains as controls. Cells were fixed and stained with Filipin as described [11]. Briefly, yeast cultures were treated with formaldehyde (3%) for 1 h on ice to fix cells. Then, cells were centrifuged, washed once with 1xPBS, re-suspended in Filipin staining buffer (500 μL 1xPBS and 20 μl of 5 mg/L Filipin solution) and incubated at 4°C in the dark for overnight. After incubation, 1 μl of the cell suspensions were spotted onto poly-lysine microscope slides and subjected to a fluorescence microscope (UV filter set) as described [11].

**TBSV-derived vsiRNA detection in *N. benthamiana***

NbFis1-silenced and control (TBV-MBP) plants were inoculated with TBSV sap 12 d post agroinfiltration. Samples (0.5 g) from the inoculated leaves were collected 2 d post inoculation for plant RNA isolation. The collected samples were ground in liquid nitrogen and mixed with 5 mL TRIzol for total RNA isolation. After thoroughly mixing, 1.2 mL Chloroform was added, then the solution was further vortexed thoroughly and put on ice for 5 min. Then it was centrifuged at 6,600 rpm and 4 °C for 30 min in a swing bucket centrifuge. The supernatant was mixed with equal volume of isopropanol and stored at -20 °C overnight. The mix was then centrifuged at 6,600 rpm and 4 °C for 30 min in a swing bucket centrifuge. The pellet was washed with 70% ethanol and finally dissolved with RNase-free H_2_O. The concentration of the isolated plant total RNA were determined by a NanoDrop One spectrophotometer. Total RNA was firstly loaded onto 0.8% agarose gel to balance the samples based on the ribosomal RNA. Total RNA (~15 µg) was loaded onto 15% denaturing polyacrylamide gel containing 7M Urea. The RNA transfer and hybridization were performed as previously [12]. T7 polymerase made DI72 (-)RNA was used as a ^32^P-labeled probe to detect the vsiRNAs.

**Plasmids described in previous studies.** The references for the plasmids listed in S2 Table are described in the following publications: [3,12-21].

**References:**

1. Snider J, Kittanakom S, Curak J, Stagljar I (2010) Split-ubiquitin based membrane yeast two-hybrid (MYTH) system: a powerful tool for identifying protein-protein interactions. J Vis Exp.

2. Mendu V, Chiu M, Barajas D, Li Z, Nagy PD (2010) Cpr1 cyclophilin and Ess1 parvulin prolyl isomerases interact with the tombusvirus replication protein and inhibit viral replication in yeast model host. Virology 406: 342-351.

3. Li Z, Barajas D, Panavas T, Herbst DA, Nagy PD (2008) Cdc34p ubiquitin-conjugating enzyme is a component of the tombusvirus replicase complex and ubiquitinates p33 replication protein. J Virol 82: 6911-6926.

4. Win J, Kamoun S, Jones AM (2011) Purification of effector-target protein complexes via transient expression in Nicotiana benthamiana. Methods Mol Biol 712: 181-194.

5. Rajendran KS, Nagy PD (2003) Characterization of the RNA-binding domains in the replicase proteins of tomato bushy stunt virus. J Virol 77: 9244-9258.

6. Barajas D, Jiang Y, Nagy PD (2009) A unique role for the host ESCRT proteins in replication of Tomato bushy stunt virus. PLoS Pathog 5: e1000705.

7. Cheng X, Deng P, Cui H, Wang A (2015) Visualizing double-stranded RNA distribution and dynamics in living cells by dsRNA binding-dependent fluorescence complementation. Virology 485: 439-451.

8. Bachan S, Dinesh-Kumar SP (2012) Tobacco rattle virus (TRV)-based virus-induced gene silencing. Methods Mol Biol 894: 83-92.

9. Jaag HM, Nagy PD (2009) Silencing of Nicotiana benthamiana Xrn4p exoribonuclease promotes tombusvirus RNA accumulation and recombination. Virology 386: 344-352.

10. Yoo SD, Cho YH, Sheen J (2007) Arabidopsis mesophyll protoplasts: a versatile cell system for transient gene expression analysis. Nat Protoc 2: 1565-1572.

11. Barajas D, Xu K, de Castro Martin IF, Sasvari Z, Brandizzi F, et al. (2014) Co-opted Oxysterol-Binding ORP and VAP Proteins Channel Sterols to RNA Virus Replication Sites via Membrane Contact Sites. PLoS Pathog 10: e1004388.

12. Kovalev N, Inaba JI, Li Z, Nagy PD (2017) The role of co-opted ESCRT proteins and lipid factors in protection of tombusviral double-stranded RNA replication intermediate against reconstituted RNAi in yeast. PLoS Pathog 13: e1006520.

13. Xu K, Nagy PD (2016) Enrichment of Phosphatidylethanolamine in Viral Replication Compartments via Co-opting the Endosomal Rab5 Small GTPase by a Positive-Strand RNA Virus. PLoS Biol 14: e2000128.

14. Xu K, Lin JY, Nagy PD (2014) The hop-like stress-induced protein 1 cochaperone is a novel cell-intrinsic restriction factor for mitochondrial tombusvirus replication. J Virol 88: 9361-9378.

15. Chuang C, Prasanth KR, Nagy PD (2017) The Glycolytic Pyruvate Kinase Is Recruited Directly into the Viral Replicase Complex to Generate ATP for RNA Synthesis. Cell Host Microbe 22: 639-652 e637.

16. Lin W, Wang L, Yan W, Chen L, Chen H, et al. (2017) Identification and characterization of Bamboo mosaic virus isolates from a naturally occurring coinfection in Bambusa xiashanensis. Arch Virol 162: 1335-1339.

17. Li Z, Pogany J, Panavas T, Xu K, Esposito AM, et al. (2009) Translation elongation factor 1A is a component of the tombusvirus replicase complex and affects the stability of the p33 replication co-factor. Virology 385: 245-260.

18. Barajas D, Li Z, Nagy PD (2009) The Nedd4-type Rsp5p ubiquitin ligase inhibits tombusvirus replication by regulating degradation of the p92 replication protein and decreasing the activity of the tombusvirus replicase. J Virol 83: 11751-11764.

19. Wu CY, Nagy PD (2019) Blocking tombusvirus replication through the antiviral functions of DDX17-like RH30 DEAD-box helicase. PLoS Pathog 15: e1007771.

20. Xu K, Nagy PD (2015) RNA virus replication depends on enrichment of phosphatidylethanolamine at replication sites in subcellular membranes. Proc Natl Acad Sci U S A 112: E1782-E1791.

21. Panavas T, Serviene E, Brasher J, Nagy PD (2005) Yeast genome-wide screen reveals dissimilar sets of host genes affecting replication of RNA viruses. Proc Natl Acad Sci U S A 102: 7326-7331.
