# Supplementary material for: Dynamic interplay between the co-opted Fis1 mitochondrial fission protein and membrane contact site proteins in supporting tombusvirus replication
Source: PLoS Pathog. 2021 Mar 16;17(3):e1009423. doi: 10.1371/journal.ppat.1009423 (PMC7997005; doi:10.1371/journal.ppat.1009423)
Supplement: S1 Table — (DOCX) [file ppat.1009423.s014.docx]

**S1 Table**

| **List of plasmids constructed in this study** | | | | | | |
| --- | --- | --- | --- | --- | --- | --- |
| No. | Plasmid name | insert source | insert digestion sites | primers for insert amplification | vector source | vector digestion sites |
| No. 1 | UpYES-NT-HisScFis1 | Yeast genome DNA | BamHI and XhoI | #7206 and #7207 | UpYES-NT | BamHI and XhoI |
| No. 2 | UpYES-HisScFis1∆C25 | UpYES-NT-HisScFis1 | BamHI and XhoI | #7206 and #7211 | UpYES-NT | BamHI and XhoI |
| No. 3 | UpYES-HisScFis1∆C63 | UpYES-NT-HisScFis1 | BamHI and XhoI | #7206 and #7212 | UpYES-NT | BamHI and XhoI |
| No. 4 | UpYES-HisScFis1∆N18 | UpYES-NT-HisScFis1 | BamHI and XhoI | #7208 and #7207 | UpYES-NT | BamHI and XhoI |
| No. 5 | UpYES-HisScFis1∆N54 | UpYES-NT-HisScFis1 | BamHI and XhoI | #7209 and #7207 | UpYES-NT | BamHI and XhoI |
| No. 6 | UpYES-HisScFis1∆N92 | UpYES-NT-HisScFis1 | BamHI and XhoI | #7210 and #7207 | UpYES-NT | BamHI and XhoI |
| No. 7 | UpYC-ScSac1 | Yeast genome DNA | BamHI and XhoI | #2508 and #2509 | UpYC-NT | BamHI and XhoI |
| No. 8 | UpESC-ScDnm1 | Yeast genome DNA | BamHI and XhoI | #7189 and #7190 | UpESC-empty | BamHI and XhoI |
| No. 9 | LpGAD-HisScFis1 | UpYES-NT-HisScFis1 | BamHI and XhoI | #7206 and #7207 | LpGAD-His92 | BamHI and XhoI |
| No. 10 | pPR-N-RE-AtFis1A | *A. thaliana* cDNA | BamHI and EcoRI | #7200 and #7228 | pPR-N-RE | BamHI and EcoRI |
| No. 11 | pPR-N-RE-AtFis1B | *A. thaliana* cDNA | BamHI and EcoRI | #7202 and #7229 | pPR-N-RE | BamHI and EcoRI |
| No. 12 | pPR-N-RE-ScFis1 | UpYES-NT-HisScFis1 | BamHI and XhoI | #7206 and #7207 | pPR-N-RE | BamHI and SalI |
| No. 13 | pPR-N-RE-ScFis1∆N18 | UpYES-NT-HisScFis1 | BamHI and NcoI | #7208 and #7231 | pPR-N-RE | BamHI and NcoI |
| No. 14 | pPR-N-RE-ScFis1∆N54 | UpYES-NT-HisScFis1 | BamHI and NcoI | #7209 and #7231 | pPR-N-RE | BamHI and NcoI |
| No. 15 | pPR-N-RE-ScFis1∆N92 | UpYES-NT-HisScFis1 | BamHI and NcoI | #7210 and #7231 | pPR-N-RE | BamHI and NcoI |
| No. 16 | pPR-N-RE-ScFis1∆C25 | UpYES-NT-HisScFis1 | BamHI and NcoI | #7206 and #7234 | pPR-N-RE | BamHI and NcoI |
| No. 17 | pPR-N-RE-ScFis1∆C63 | UpYES-NT-HisScFis1 | BamHI and NcoI | #7206 and #7233 | pPR-N-RE | BamHI and NcoI |
| No. 18 | pPR-N-RE-ScSac1 | Yeast genome DNA | BamHI and XhoI | #2508 and #2509 | pPR-N-RE | BamHI and XhoI |
| No. 19 | UpYES-HisAtFis1A | *A. thaliana* cDNA | BamHI and XhoI | #7200 and #7201 | UpYES-NT | BamHI and XhoI |
| No. 20 | UpYES-HisAtFis1B | *A. thaliana* cDNA | BamHI and XhoI | #7202 and #7203 | UpYES-NT | BamHI and XhoI |
| No. 21 | pGD-35S-T33-cHA | HpGBK-Gal-HisT33/Gal-DI-72 | BglII and XhoI | #4000 and #7833 | pGD-35S | BamHI and XhoI |
| No. 22 | pGD-35S-GFP-cHA | pGD-35S-GFP-SKL | BamHI and XhoI | #6511 and #3712 | pGD-35S | BamHI and XhoI |
| No. 23 | pGD-35S-Flag-AtFis1A | *A. thaliana* cDNA | BamHI and XhoI | #7200 and #7201 | pGD-2x35S-N-Flag | BamHI and SalI |
| No. 24 | pGD-35S-Flag-AtFis1B | *A. thaliana* cDNA | BamHI and XhoI | #7202 and #7203 | pGD-2x35S-N-Flag | BamHI and SalI |
| No. 25 | pGEX-His-RE-ScFis1-∆TM | UpYES-NT-HisScFis1 | BamHI and XhoI | #7206 and #7211 | pGEX-His-RE | BamHI and XhoI |
| No. 26 | pGEX-His-RE-AtFis1A-∆TM | UpYES-HisAtFis1A | BamHI and SalI | #7200 and #7349 | pGEX-His-RE | BamHI and XhoI |
| No. 27 | pGEX-His-RE-AtFis1B-∆TM | UpYES-HisAtFis1B | BamHI and SalI | #7202 and #7350 | pGEX-His-RE | BamHI and XhoI |
| No. 28 | UpYC-ScFis1 | Yeast genome DNA | BamHI and XhoI | #7206 and #7207 | UpYC-NT | BamHI and XhoI |
| No. 29 | UpYC-YFP-ScFis1 | UpESC-YFP-p33 | HindIII and BamHI | #1292 and #1295 | UpYC-ScFis1 | HindIII and BamHI |
| No. 30 | pGD-35S-BFP-AtFis1A | UpYES-HisAtFis1A | BamHI and XhoI | #7200 and #7201 | pGD-2x35S-BFP-nFlag | BamHI and SalI |
| No. 31 | pGD-35S-BFP-AtFis1B | UpYES-HisAtFis1B | BamHI and XhoI | #7202 and #7203 | pGD-2x35S-BFP-nFlag | BamHI and SalI |
| No. 32 | pGD-35S-nYFP-AtFis1A | UpYES-HisAtFis1A | BamHI and XhoI | #7200 and #7201 | pGD-nYFP-MBP | BamHI and SalI |
| No. 33 | pGD-35S-nYFP-AtFis1B | UpYES-HisAtFis1B | BamHI and XhoI | #7202 and #7203 | pGD-nYFP-MBP | BamHI and SalI |
| No. 34 | pGD-35S-cYFP-NT | pGD-T33-cYFP | BglII and SpeI | #5908 and #5909 | pGD-35S | BamHI and XbaI |
| No. 35 | pGD-35S-cYFP-AtFis1A | UpYES-HisAtFis1A | XhoI | #7642 and #7201 | pGD-35S-cYFP-NT | SalI |
| No. 36 | pGD-35S-cYFP-AtFis1B | UpYES-HisAtFis1B | XhoI | #7643 and #7203 | pGD-35S-cYFP-NT | SalI |
| No. 37 | pGD-35S-nYFP-AtVAP27-1 | *A. thaliana* cDNA | BamHI and XhoI | #3458 and #3459 | pGD-nYFP-MBP | BamHI and SalI |
| No. 38 | pGD-35S-nYFP-AtPVA12 | *A. thaliana* cDNA | BamHI and XhoI | #4252 and #4253 | pGD-nYFP-MBP | BamHI and SalI |
| No. 39 | pGD-35S-nYFP-AtORP3A | *A. thaliana* cDNA | BamHI and XhoI | #5477 and #5478 | pGD-nYFP-MBP | BamHI and SalI |
| No. 40 | pGD-35S-AtSacI-cYFP | *A. thaliana* cDNA | BamHI and PstI | #7644 and #7645 | pGD-C-cYFP | BamHI and PstI |
| No. 41 | TRV2-NbFis1-S1 | *N.benthamiana* cDNA | BamHI and MluI | #5812 and #5813 | pTRV2 | BamHI and MluI |
| No. 42 | TRV2-NbFis1-S2 | *N.benthamiana* cDNA | BamHI and MluI | #5814 and #5815 | pTRV2 | BamHI and MluI |
| No. 43 | pGD-35S-GFP-AtFis1B | UpYES-HisAtFis1B | BamHI and XhoI | #7202 and #7203 | pGD-2x35S-GFP-nFlag | BamHI and SalI |
| No.44 | pBT3-N-ScFis1 | UpYES-NT-HisScFis1 | PstI and NcoI | #7230 and #7231 | pBT3-N | PstI and NcoI |
|  | pGD-AtVAP27-1-GFP | AtVAP27-1 | BamHI and PstI | #3458 and #8419 | pGD-C-GFP | BamHI and PstI |
|  | pGD-GFP-AtPVA12 | AtPVA12 | BamHI&XhoI | #4252 and #4253 | pGD-C-GFP | BamHI&PstI |
|  | pGD-2xFlag-AtFis1B | AtFis1B | BamHI&SalI | #8373 and #8374 | pGD-C-GFP | BamHI&SalI |
|  | pRS315-Flag-Fis1-pex15 | Fis1 full length and Pex15 | BamHI&XhoI | #7206, #8468, #8469, #8437 (overlap PCR) | pRS315-Flag | BamHI&SalI |
|  | pRS315-Flag-p36mts-Fis1 | CIRV p36 fragment and Fis1 full length | BamHI&XhoI | #3461, #8471, #8470, #7207 (overlap PCR) | pRS315-Flag | BamHI&SalI |
