# Supplementary material for: Dynamic interplay between the co-opted Fis1 mitochondrial fission protein and membrane contact site proteins in supporting tombusvirus replication
Source: PLoS Pathog. 2021 Mar 16;17(3):e1009423. doi: 10.1371/journal.ppat.1009423 (PMC7997005; doi:10.1371/journal.ppat.1009423)
Supplement: S2 Table — (DOCX) [file ppat.1009423.s015.docx]

**S2 Table**

| **List of plasmids described in previous studies** | | |
| --- | --- | --- |
| No. | Plasmid name | Source |
| No. 45 | HpGBK-Gal-HisT33/Gal-DI-72 | Dr. Kai Xu (University of Kentucky) |
| No. 46 | LpGAD-Gal-HisT92 | Dr. Kai Xu (University of Kentucky) |
| No. 47 | HpGBK-CUP1-Hisp33/Gal-DI-72 | [17] Barajas et al., 2009 |
| No. 48 | LpGAD-CUP1-Hisp92 | [17] Barajas et al., 2009 |
| No. 49 | HpESC-CUP1-Hisp36/Gal-DI-72 | Dr. J. Pogany (University of Kentucky) |
| No. 50 | LpESC-CUP1-Hisp95 | Dr. J. Pogany (University of Kentucky) |
| No. 51 | pESC-His/Gal/FHV/RNA1/Frameshift/TRSVRRZ | Dr. J. Pogany (University of Kentucky) |
| No. 52 | pGAD-Leu/Cup/FHV/Protein-A/C-term/HA/FLAG | Dr. J. Pogany (University of Kentucky) |
| No. 53 | UpYC-ScOsh6 | [11] Barajas et al., 2014 |
| No. 54 | UpYC-ScScs2 | [11] Barajas et al., 2014 |
| No. 55 | pESC-Ura-Gal10-HisDcr1 | [18] Kovalev et al., 2017 |
| No. 56 | pESC-Ura-Gal1-HisAgo1-Gal10-HisDcr1 | [18] Kovalev et al., 2017 |
| No. 57 | UpBG1805-ScDnm1-ZZ | openbiosystems |
| No. 58 | LpGAD-His92 | [21] Panavas et al., 2005 |
| No. 59 | pGAD-BT2-N-His33 | [3] Li et al., 2008 |
| No. 60 | pPR-N-RE-ssa1 | [16] Li et al., 2009 |
| No. 61 | pPRN-P33 | [11] Barajas et al., 2014 |
| No. 62 | pPRN-Scs2p | [11] Barajas et al., 2014 |
| No. 63 | pPRN-AtVAP27-1 | [11] Barajas et al., 2014 |
| No. 64 | pPRN-AtVAP27-2 | [11] Barajas et al., 2014 |
| No. 65 | pPRN-Osh6p | [11] Barajas et al., 2014 |
| No. 66 | HpGBK-CUP1-Flagp33/Gal-DI-72 | [17] Barajas et al., 2009 |
| No. 67 | LpGAD-CUP1-Flag92 | [17] Barajas et al., 2009 |
| No. 68 | HpGBK-CUP1-Flagp36/Gal-DI-72 | Dr. J. Pogany (University of Kentucky) |
| No. 69 | LpGAD-CUP1-Flag95 | Dr. J. Pogany (University of Kentucky) |
| No. 70 | pGD-35S-p19 | [12] Xu and Nagy, 2016 |
| No. 71 | pMALc-2X-T33C | [12] Xu and Nagy, 2016 |
| No. 72 | pMALc-2X-C36C | [12] Xu and Nagy, 2016 |
| No. 73 | LpRS315-pex13-RFP | [20] Xu and Nagy, 2015 |
| No. 74 | HpEsc-BFP-T33/GAL-DI-72 | [12] Xu and Nagy, 2016 |
| No. 75 | pGD-35S-T33-RFP | [12] Xu and Nagy, 2016 |
| No. 76 | pGD-35S- C36-RFP | [12] Xu and Nagy, 2016 |
| No. 77 | pGD-35S-GFP-SKL | [12] Xu and Nagy, 2016 |
| No. 78 | pGD-35S-GFP-AtTim21 | [12] Xu and Nagy, 2016 |
| No. 79 | pGD-35S-T33-cYFP | [12] Xu and Nagy, 2016 |
| No. 80 | pGD-35S-C36-cYFP | [12] Xu and Nagy, 2016 |
| No. 81 | pGD-35S-C-cYFP | [12] Xu and Nagy, 2016 |
| No. 82 | pGD-35S-nYFP-MBP | [12] Xu and Nagy, 2016 |
| No. 83 | pGD-35S-RFP-SKL | [12] Xu and Nagy, 2016 |
| No. 84 | pGD-35S-RFP-AtTim21 | [12] Xu and Nagy, 2016 |
| No. 85 | YN-B2 | [7] Cheng et al., 2015 |
| No. 86 | YC-VP35 | [7] Cheng et al., 2015 |
| No. 87 | TRV-cGFP | [13 ]Xu et al., 2014 |
| No. 88 | pGD-MS2CP-RFP | [19]Wu and Nagy, 2019 |
| No. 89 | pGD-(+)DI72-MS2hp | [19]Wu and Nagy, 2019 |
| No. 90 | pGD-(-)DI72-MS2hp | [19]Wu and Nagy, 2019 |
| No.91 | pESC-LEU/Cup/NOV/ProteinA/C-Term/HA/FLAG | Dr. J. Pogany (University of Kentucky) |
| No.92 | pESC-HIS/Cup/NOV/RNA1/Framshift/TRSV-RZ | Dr. J. Pogany (University of Kentucky) |
| No. 93 | pYes-6xHis-p92 | [12] Xu and Nagy, 2016 |
| No. 94 | pEsc-6xHis-p33-Gal-DI72 | [12] Xu and Nagy, 2016 |
| No. 95 | pGD-GFP-AtSac1 | Dr. Z. Sasvari (University of Kentucky) |
