# Supplementary material for: Dynamic interplay between the co-opted Fis1 mitochondrial fission protein and membrane contact site proteins in supporting tombusvirus replication
Source: PLoS Pathog. 2021 Mar 16;17(3):e1009423. doi: 10.1371/journal.ppat.1009423 (PMC7997005; doi:10.1371/journal.ppat.1009423)
Supplement: S3 Table — (DOCX) [file ppat.1009423.s016.docx]

**S3 Table**

| **List of primers used in this study** | | |
| --- | --- | --- |
| No. | Name | Sequence(5' to 3') |
| 1 | #7206 | CGCGGATCCATGACCAAAGTAGATTTTTGGCC |
| 2 | #7207 | CCGCTCGAGTTACCTTCTCTTGTTTCTTAAGAAG |
| 3 | #7211 | CCGCTCGAGTTACTTGAGTGTTTCCTTCTGGATC |
| 4 | #7212 | CCGCTCGAGTTATTCACCGAGTTTGTAGCAACC |
| 5 | #7208 | CGCGGATCCATGCCGCAGCAGCTGGAGATT |
| 6 | #7209 | CGCGGATCCATGGTGAATGACGAAAGGCTTGG |
| 7 | #7210 | CGCGGATCCATGTACTCTATGGCGAAGAGATATGTAG |
| 8 | #2508 | CGCGGATCCATGACAGGTCCAATAGTGTACGTTC |
| 9 | #2509 | CGCCTCGAGTTAATCTCTTTTTAAAGGATCCGG |
| 10 | #7189 | CGCGGATCCATGGCTAGTTTAGAAGATCTTATTCC |
| 11 | #7190 | CCGCTCGAGTTACAGAATATTACTAATAAGGGTTGCAG |
| 12 | #7200 | CGCGGATCCATGGATGCTAAGATCGGACAATTC |
| 13 | #7201 | CCGCTCGAGTCATTTCTTGCGAGACATCGCTG |
| 14 | #7202 | CGCGGATCCATGGACGCGGCGATAGGGAA |
| 15 | #7203 | CCGCTCGAGTTAGCTGCGTAATATGGCTGCAGC |
| 16 | #4000 | CCAGAGATCTATGGAGACCATCAAGAGAATG |
| 17 | #7833 | ACCGCTCGAGCTAAGCGTAATCTGGAACATCGTATGGGTATTTGACACCCAGGGACTCCT |
| 18 | #6511 | CGGGATCCATGGGTAAAGGAGAAGAACTTTTCACTGG |
| 19 | #3712 | CGGCCTCGAGTTACGCATAGTCAGGAACATCGTATGGGTAGAGTCCGGACTTGTATAGTT |
| 20 | #7349 | ACGCGTCGACTCAAACACCATCCTTTGTGATTTTGTC |
| 21 | #7350 | ACGCGTCGACTCACACACCATCTTTCACAATACGGTC |
| 22 | #1292 | CGGCAAGCTTACCATGGTGAGCAAGGGCGAGGAGCTGT |
| 23 | #1295 | CGGCGGATCCCTTGTACAGCTCGTCCATGCCGA |
| 24 | #5908 | GGAAGATCTATGGGCAGCGTGCAGCTC |
| 25 | #5909 | GGACTAGTCTTGTACAGCTCGTCCATGCC |
| 26 | #7642 | ACCGCTCGAGATGGATGCTAAGATCGGACAATTC |
| 27 | #7643 | ACCGCTCGAGATGGACGCGGCGATAGGGAA |
| 28 | #3458 | GCCGGATCCATGAGTAACATCGATCTGATTGGG |
| 29 | #3459 | CGGCTCGAGTTAGCTAGCTGTCCTCTTCATAATGTATCC |
| 30 | #7206 | CGCGGATCCATGACCAAAGTAGATTTTTGGCC |
| 31 | #7207 | CCGCTCGAGTTACCTTCTCTTGTTTCTTAAGAAG |
| 32 | #7211 | CCGCTCGAGTTACTTGAGTGTTTCCTTCTGGATC |
| 33 | #7212 | CCGCTCGAGTTATTCACCGAGTTTGTAGCAACC |
| 34 | #7208 | CGCGGATCCATGCCGCAGCAGCTGGAGATT |
| 35 | #7209 | CGCGGATCCATGGTGAATGACGAAAGGCTTGG |
| 36 | #7210 | CGCGGATCCATGTACTCTATGGCGAAGAGATATGTAG |
| 37 | #2508 | CGCGGATCCATGACAGGTCCAATAGTGTACGTTC |
| 38 | #2509 | CGCCTCGAGTTAATCTCTTTTTAAAGGATCCGG |
| 39 | #7189 | CGCGGATCCATGGCTAGTTTAGAAGATCTTATTCC |
| 40 | #7190 | CCGCTCGAGTTACAGAATATTACTAATAAGGGTTGCAG |
| 41 | #7200 | CGCGGATCCATGGATGCTAAGATCGGACAATTC |
| 42 | #7230 | CAACTGCAGGGCCATGACCAAAGTAGATTTTTGGCC |
| 43 | #7231 | CATGCCATGGTTAATCGATCCTTCTCTTGTTTCTTAAGAAG |
| 44 | #7233 | CATGCCATGGTTAATCGATTTCACCGAGTTTGTAGCAACC |
| 45 | #7234 | CATGCCATGGTTAATCGATCTTGAGTGTTTCCTTCTGGATC |
| 46 | #3458 | GCCGGATCCATGAGTAACATCGATCTGATTGGG |
| 47 | #3461 | GCCGGATCCATGGAGGGTTTGAAGGCTG |
| 48 | #4252 | GCCGGATCCATGAGTAACGAGCTTCTCAC |
| 49 | #4253 | CGGCTCGAGTCATGTCCTCTTCATAATG |
| 50 | #7206 | CGCGGATCCATGACCAAAGTAGATTTTTGGCC |
| 51 | #7207 | CCGCTCGAGTTACCTTCTCTTGTTTCTTAAGAAG |
| 52 | #8373 | CGGGATCCGATTACAAGGATGACGACGATAAGATGGACGCGGCGATAGGGA |
| 53 | #8374 | CCGACGTCGACTTAGCTGCGTAATATGGCTGCAG |
| 54 | #8419 | ATTGGCTGCAGTGTCCTCTTCATAATGTATCCCAAAAT |
| 55 | #8437 | CCGCTCGAGTCATATACTCGCTAGAAGTTTTAGCAA |
| 56 | #8468 | CCTTCTCTTGTTTCTTAAGAAGAAACTA |
| 57 | #8469 | TAGTTTCTTCTTAAGAAACAAGAGAAGGCATGTTCCAGCGGCCTTTC |
| 58 | #8470 | GTTGGTTGAGCCTGCTCGGATGACCAAAGTAGATTTTTGGCC |
| 59 | #8471 | CCGAGCAGGCTCAACCAAC |
